# Supplementary material for: Impacts of climate change on high priority fruit fly species in Australia
Source: PLoS One. 2020 Feb 13;15(2):e0213820. doi: 10.1371/journal.pone.0213820 (PMC7018044; doi:10.1371/journal.pone.0213820)
Supplement: S1 File — (DOCX) [file pone.0213820.s001.docx]

**Impacts of climate change on high priority fruit fly species in Australia**

**Sabira Sultana**^1,2^**, John B. Baumgartner**^1^**, Bernard C. Dominiak**^3^**, Jane E. Royer**^4^ **& Linda J. Beaumont**^1^

^1 Department of Biological Sciences, Macquarie University, North Ryde, New South Wales, 2109, Australia.^

^2 Department of Zoology, Jahangirnagar University, Savar, Dhaka 1342, Bangladesh^

^3 New South Wales Department of Primary Industries, Locked Bag 21, Orange, New South Wales, 2800, Australia.^

^4 Queensland Department of Agriculture and Fisheries, Biosecurity Queensland, GPO Box 267, Brisbane, Queensland, 4001, Australia.^

**Supporting Information**

**S1-11 Figs. Climatic habitat suitability for 11 tephritid fruit flies under various future climate scenarios, when novel environments are included.** (1) *Bactrocera aquilonis,* (2) *Bactrocera bryoniae,* (3) *Bactrocera frauenfeldi,* (4) *Bactrocera halfordiae,* (5) *Bactrocera jarvisi,* (6) *Bactrocera kraussi,* (7) *Bactrocera musae,* (8) *Bactrocera neohumeralis,* (9) *Bactrocera tryoni,* (10) *Ceratitis capitata,* (11) *Zeugodacus cucumis*. (A) baseline (1960-1990) habitat suitability modelled using Maxent – values close to zero represent areas with low climatic suitability while values closer to one indicate higher climatic suitability; (B) areas considered “suitable” (i.e., with habitat suitability values above the 10th percentile at training presence sites, shown in red); (C, D, E) agreement about the suitability of habitat for the species across six climate scenarios for 2030, 2050 and 2070, respectively; (F) the location of Australian occurrence records of the species, which were used to calibrate models, based on specimens from natural history collections, literature and State Government trapping programs, and major commercial horticultural hosts, according to the Australian Horticulture Statistics Handbook (HSHB; [www.horticulture.com.au](http://www.horticulture.com.au)).

1. *Bactrocera aquilonis*


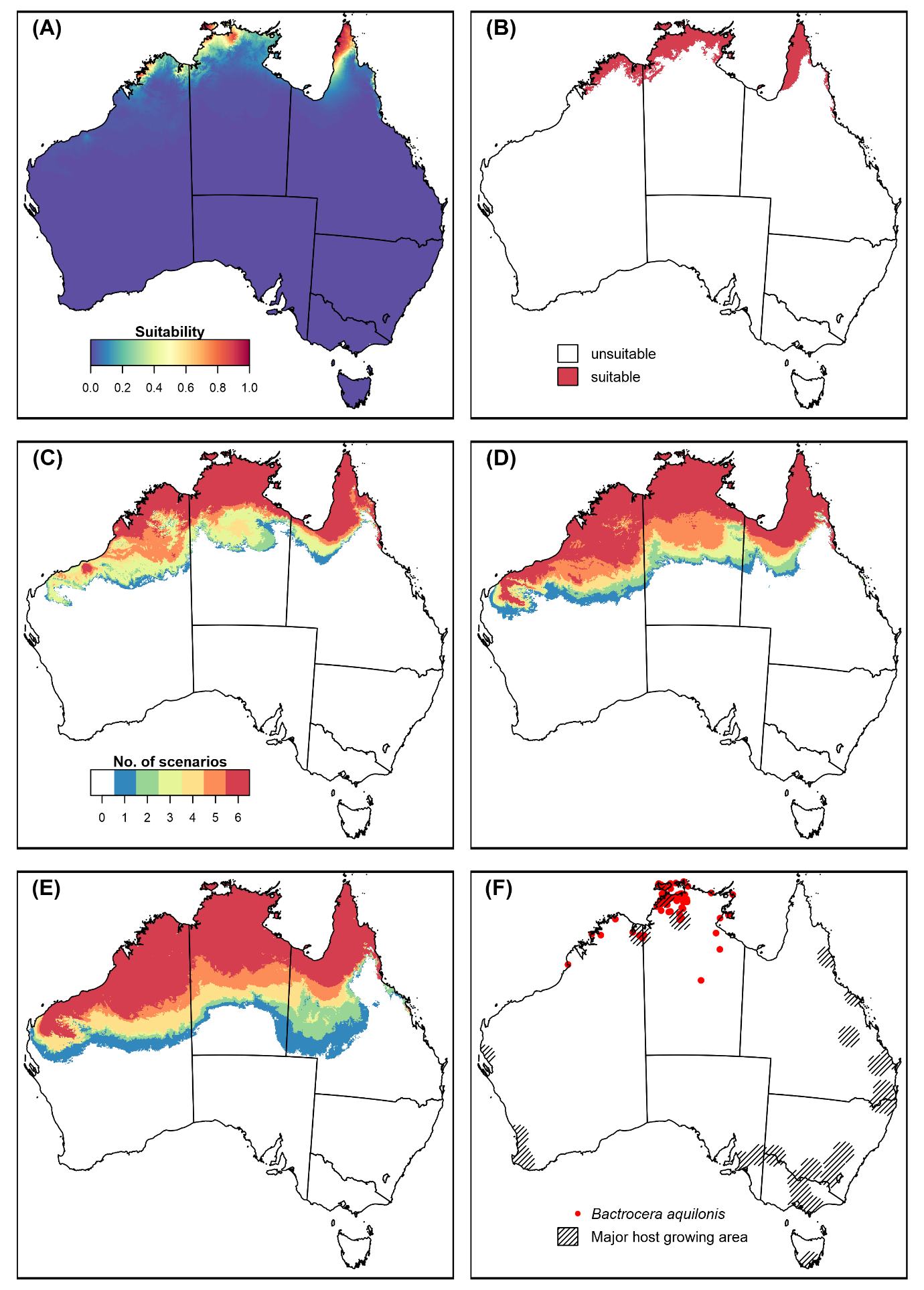


1.
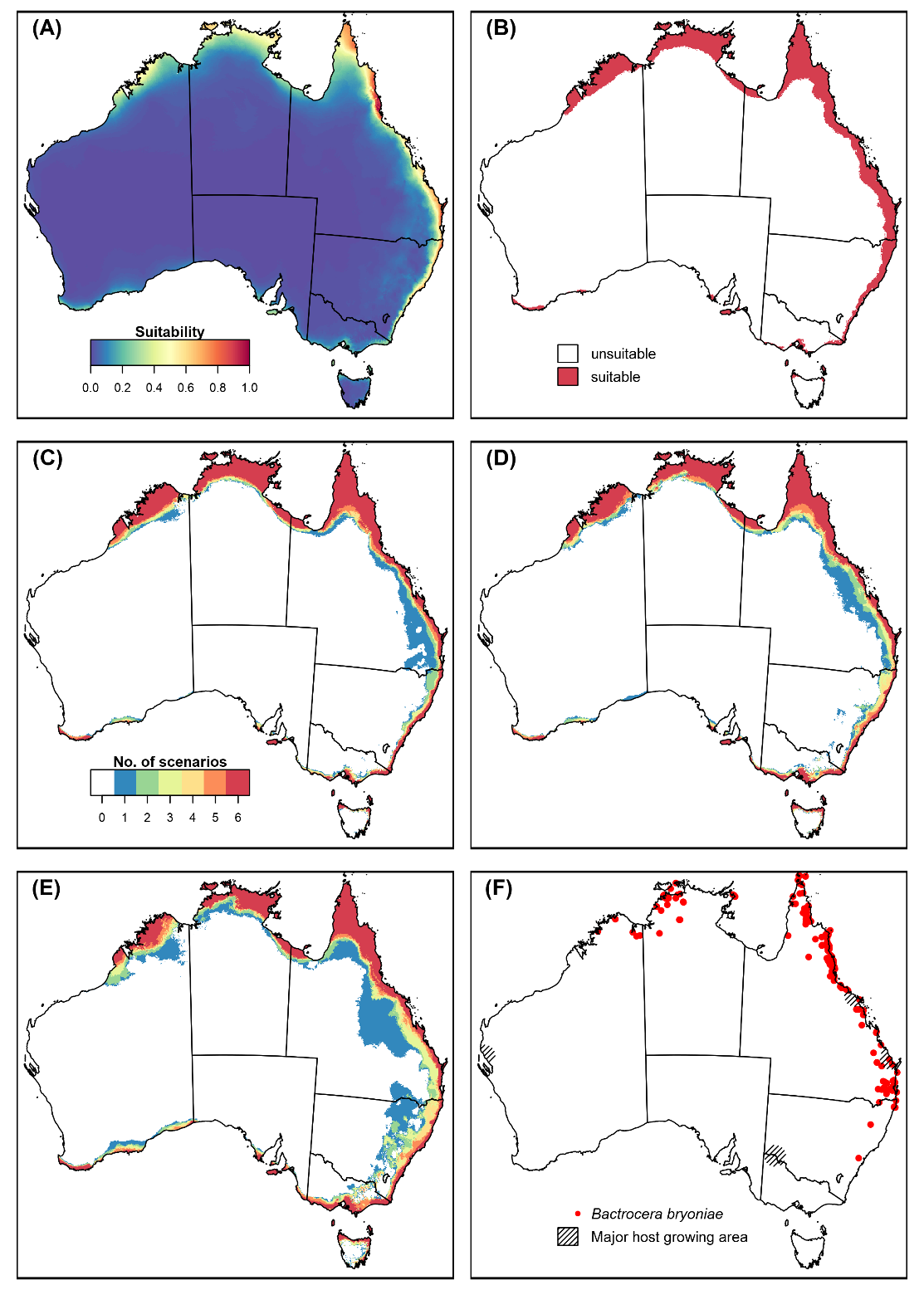
*Bactrocera bryoniae*

1. *Bactrocera frauenfeldi*


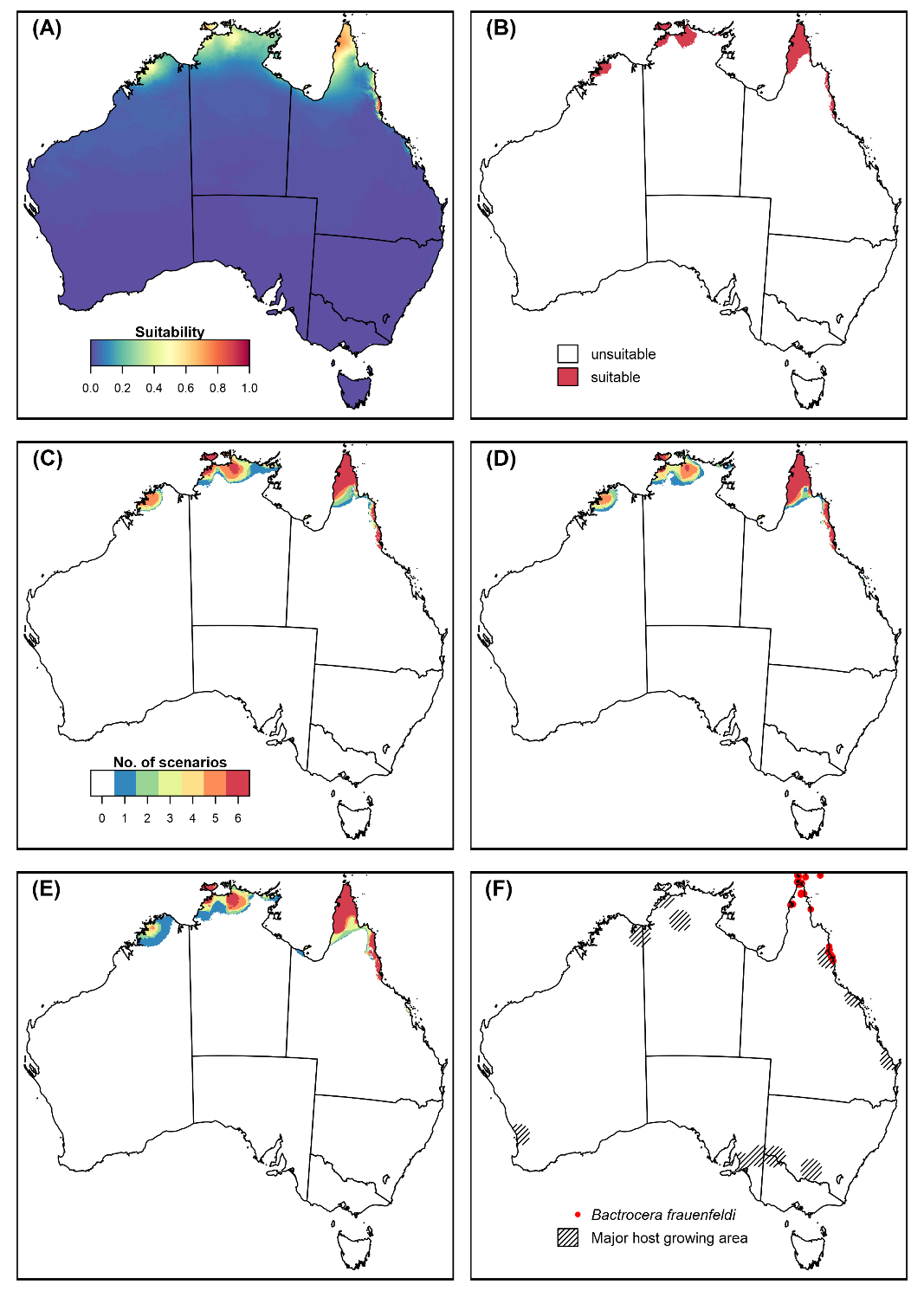


1. *Bactrocera halfordiae*


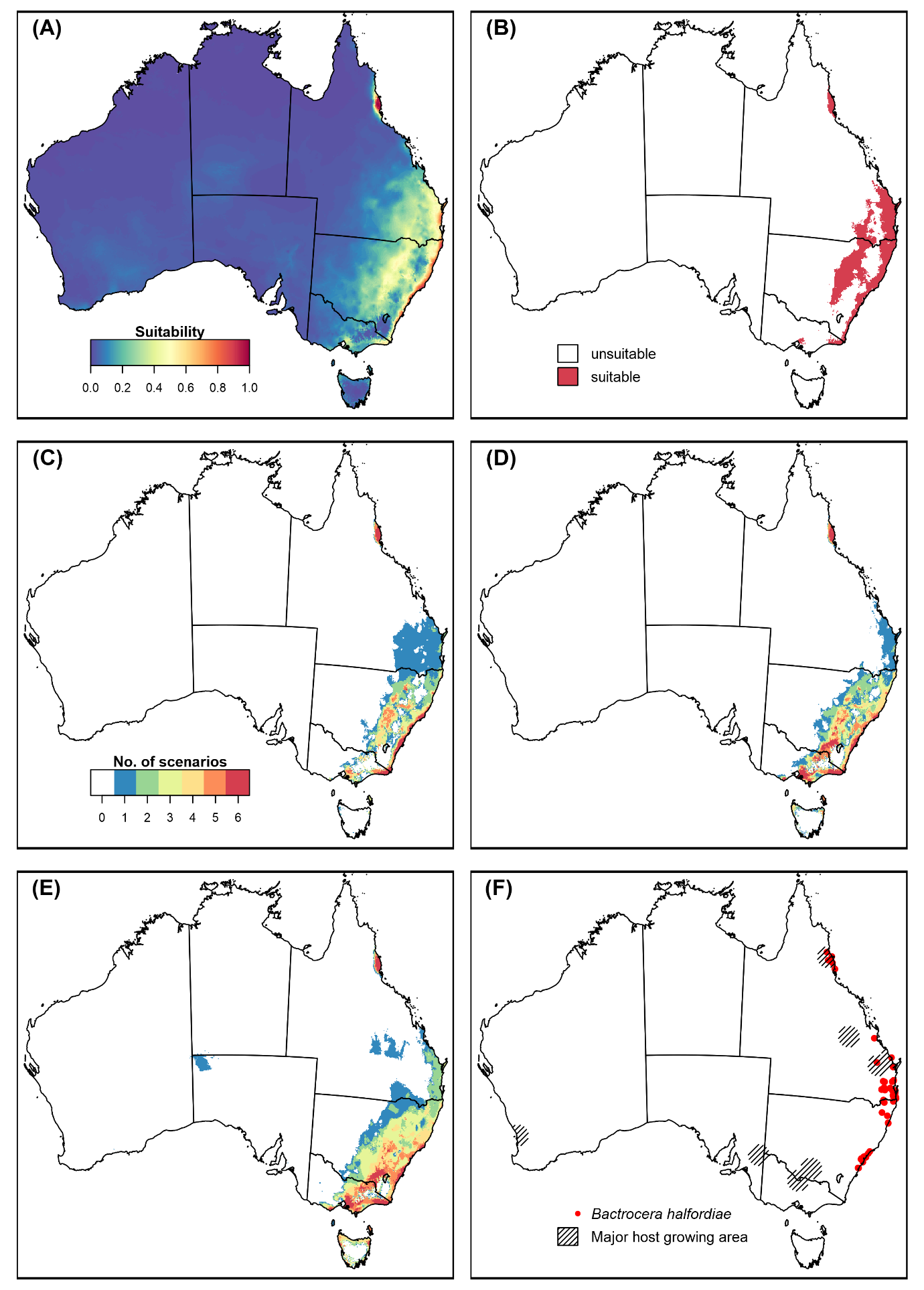


1. *Bactrocera jarvisi*


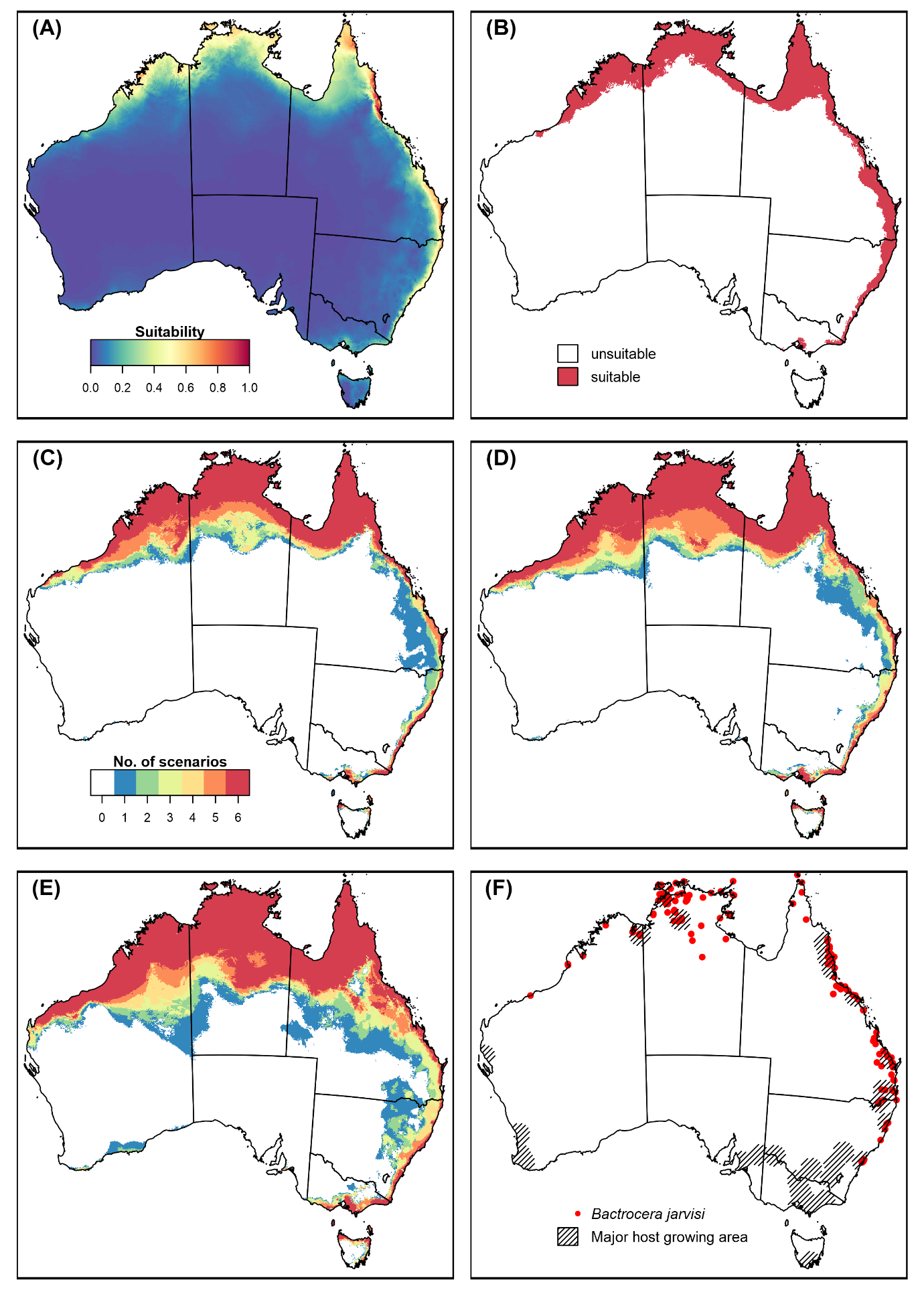


1. *Bactrocera kraussi*


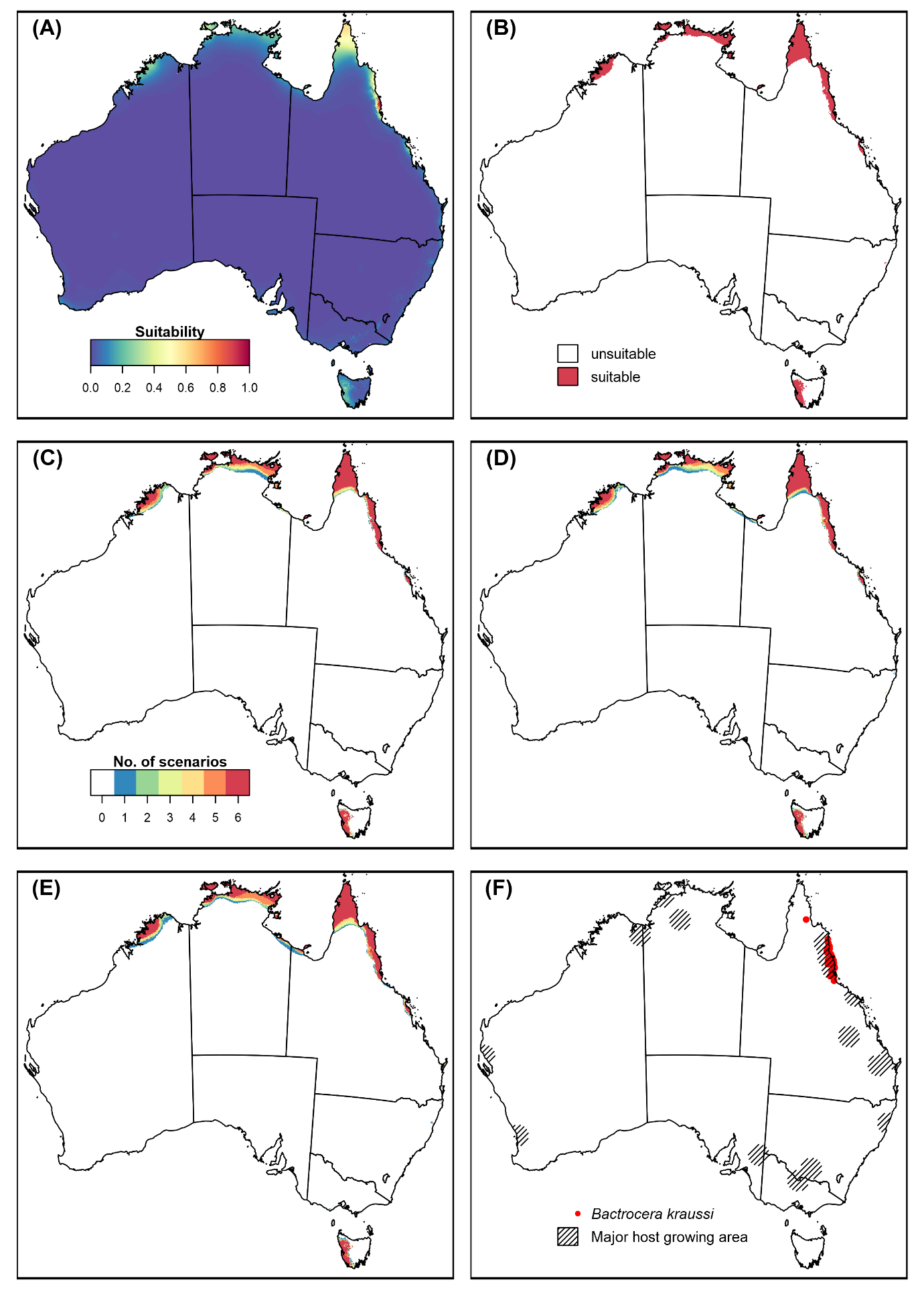


1. *Bactrocera musae*


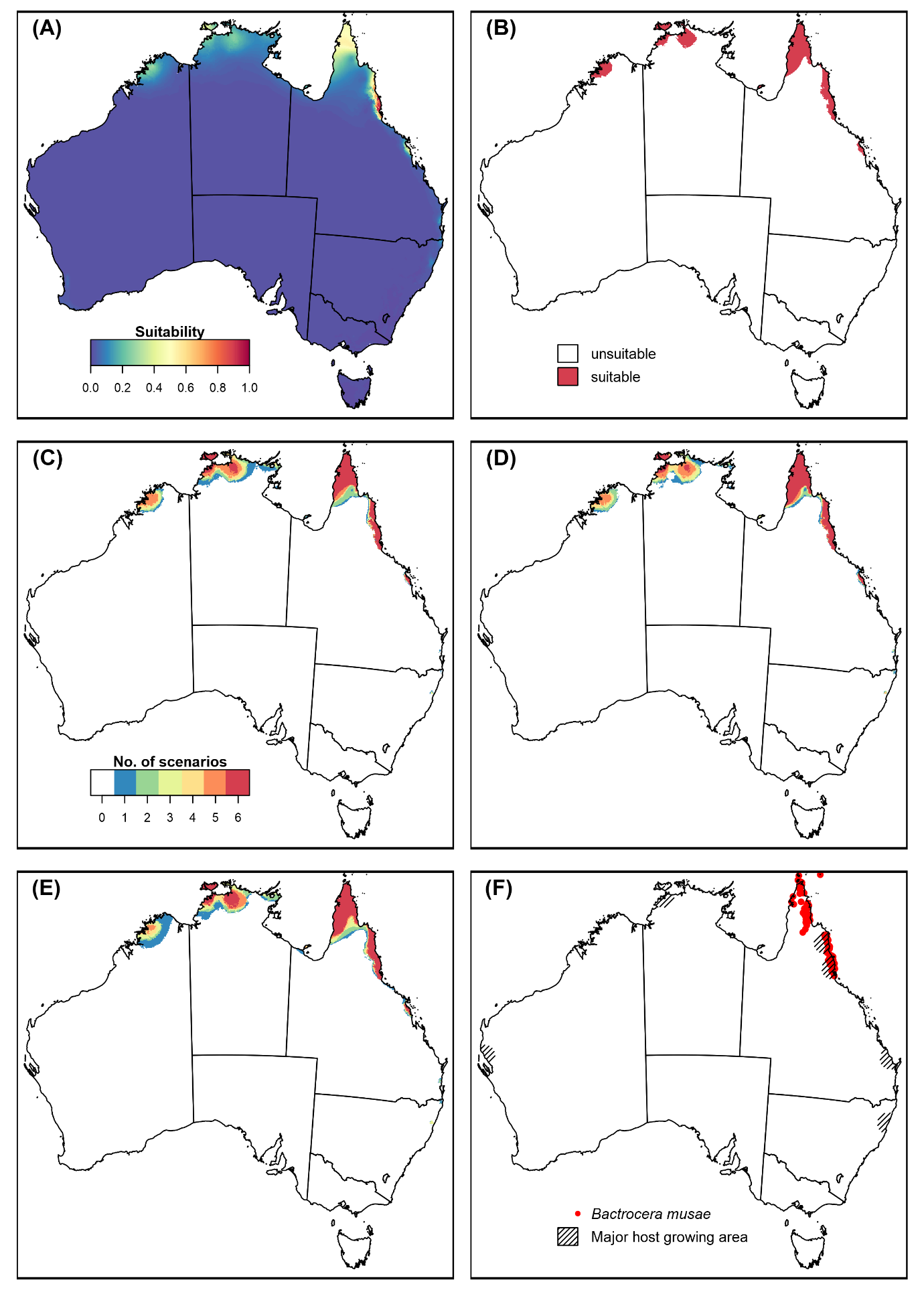


1. *Bactrocera neohumeralis*


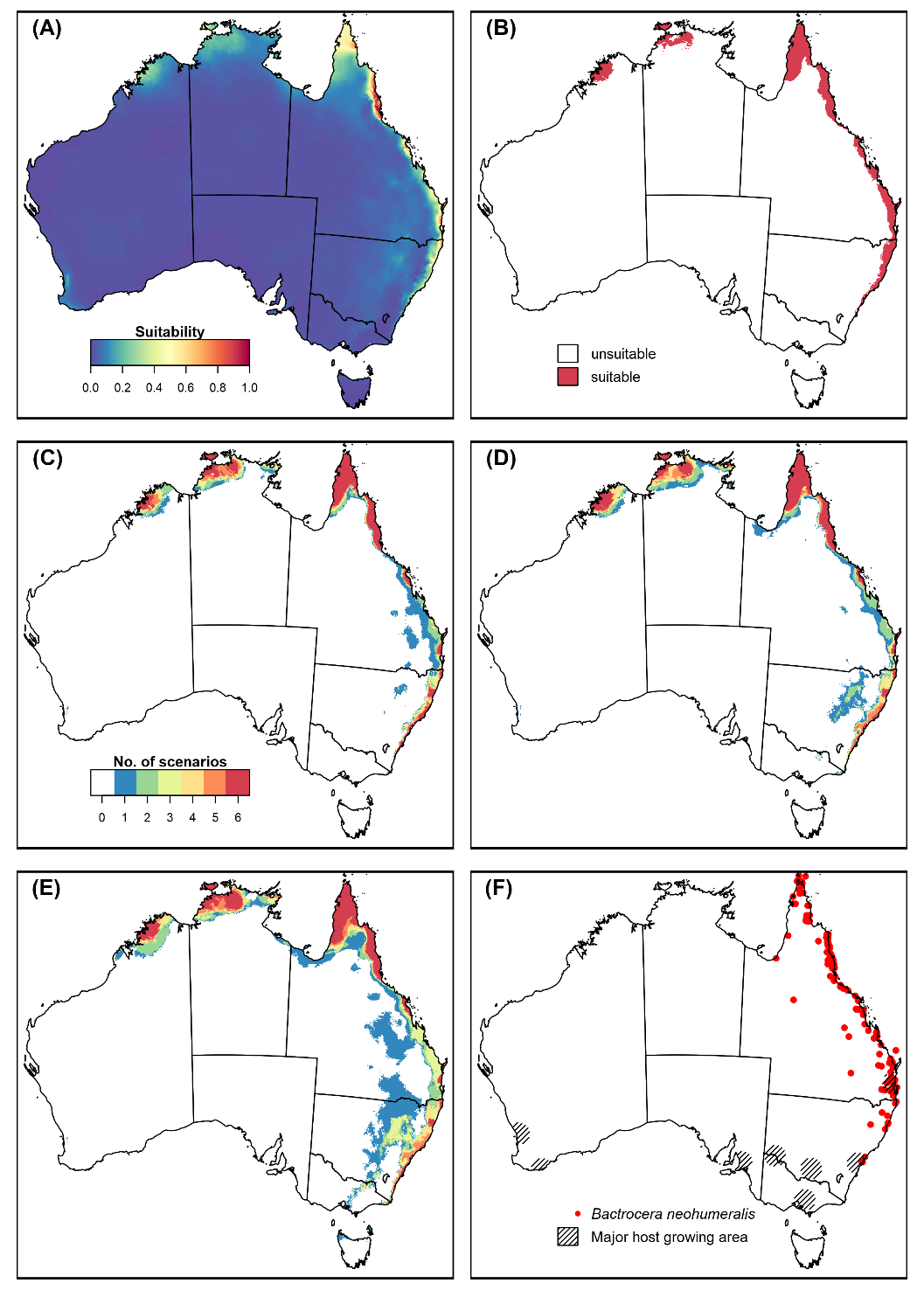


1. *Bactrocera tryoni*


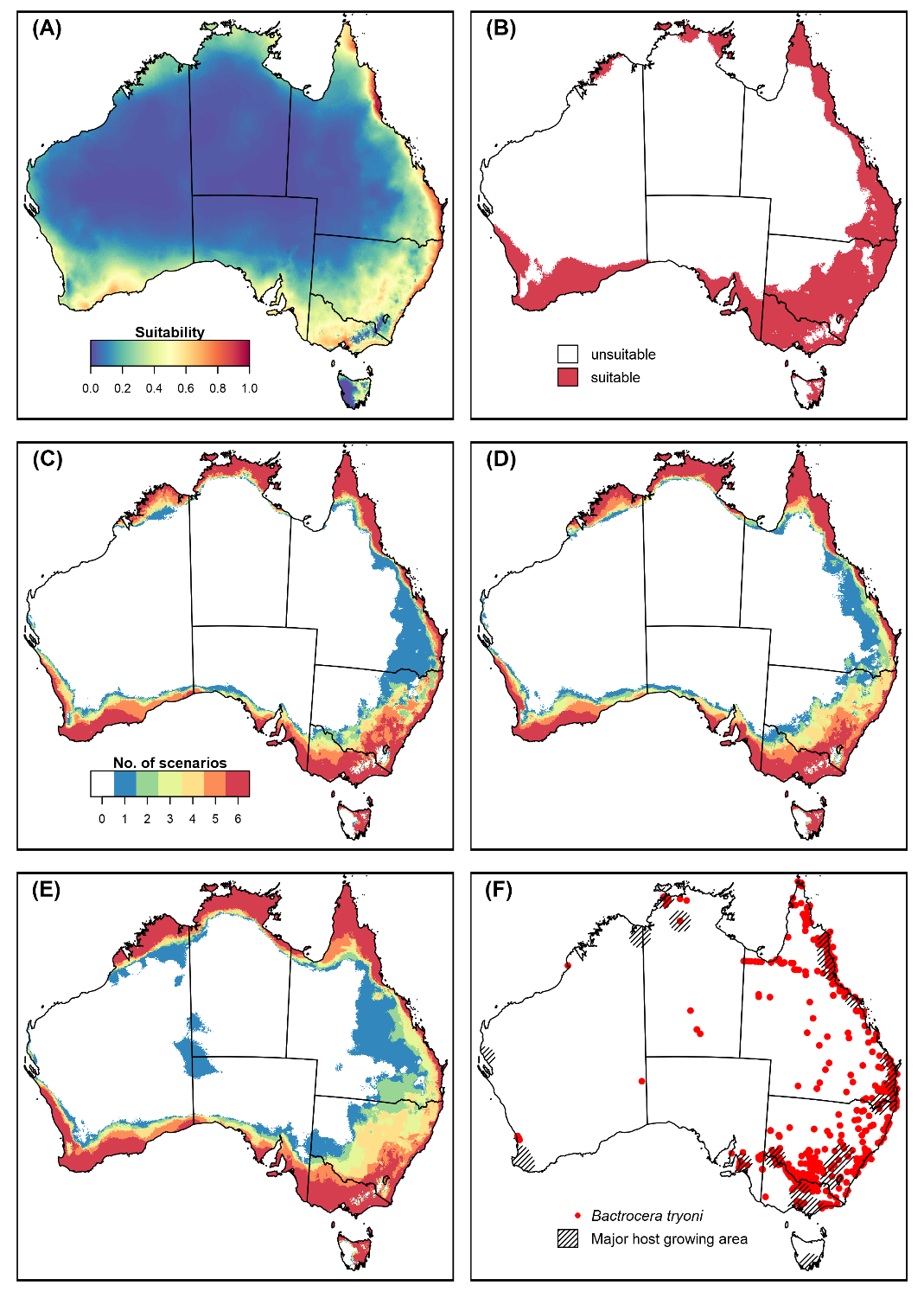


1. *Ceratitis capitata*

**
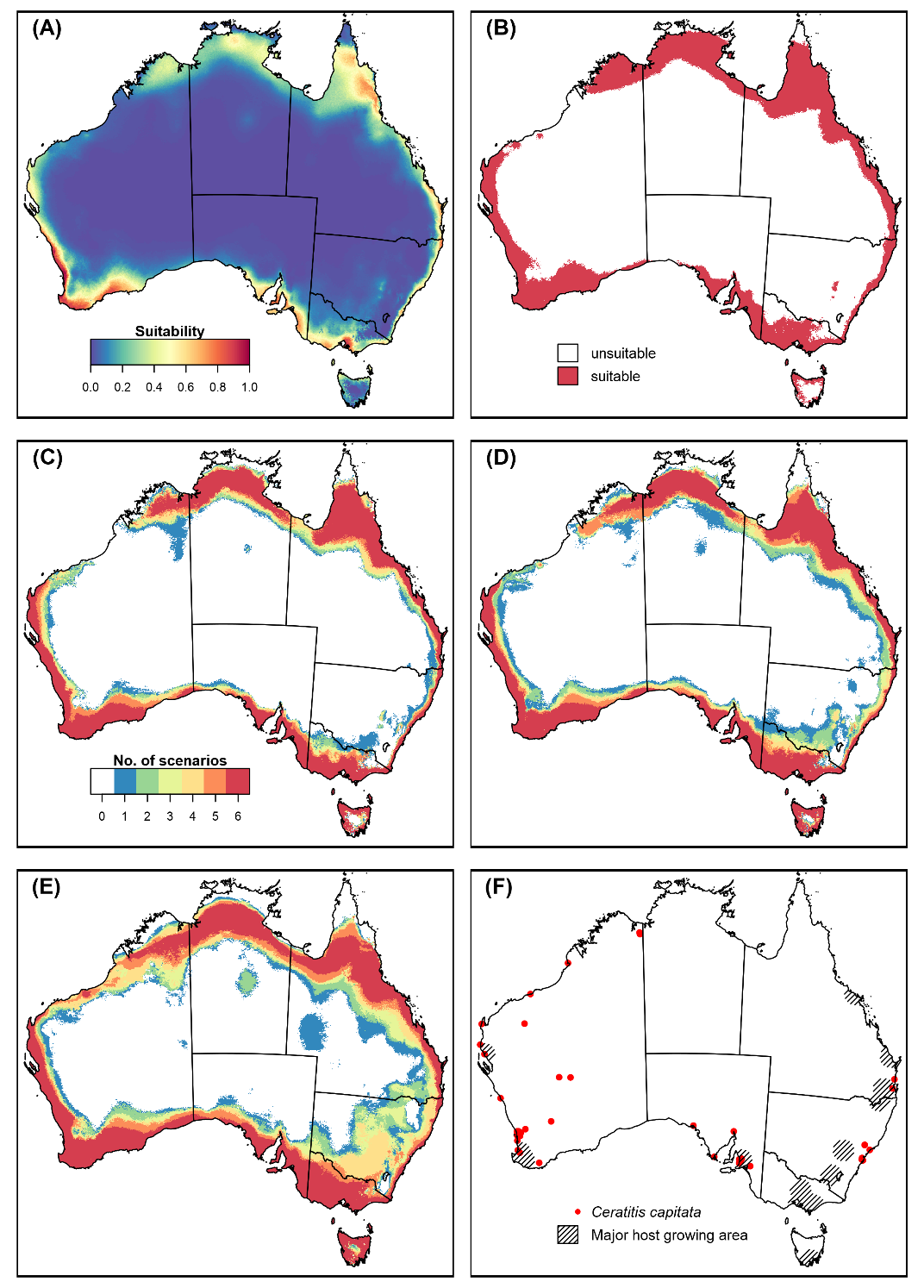
**

1. *Zeugodacus cucumis*


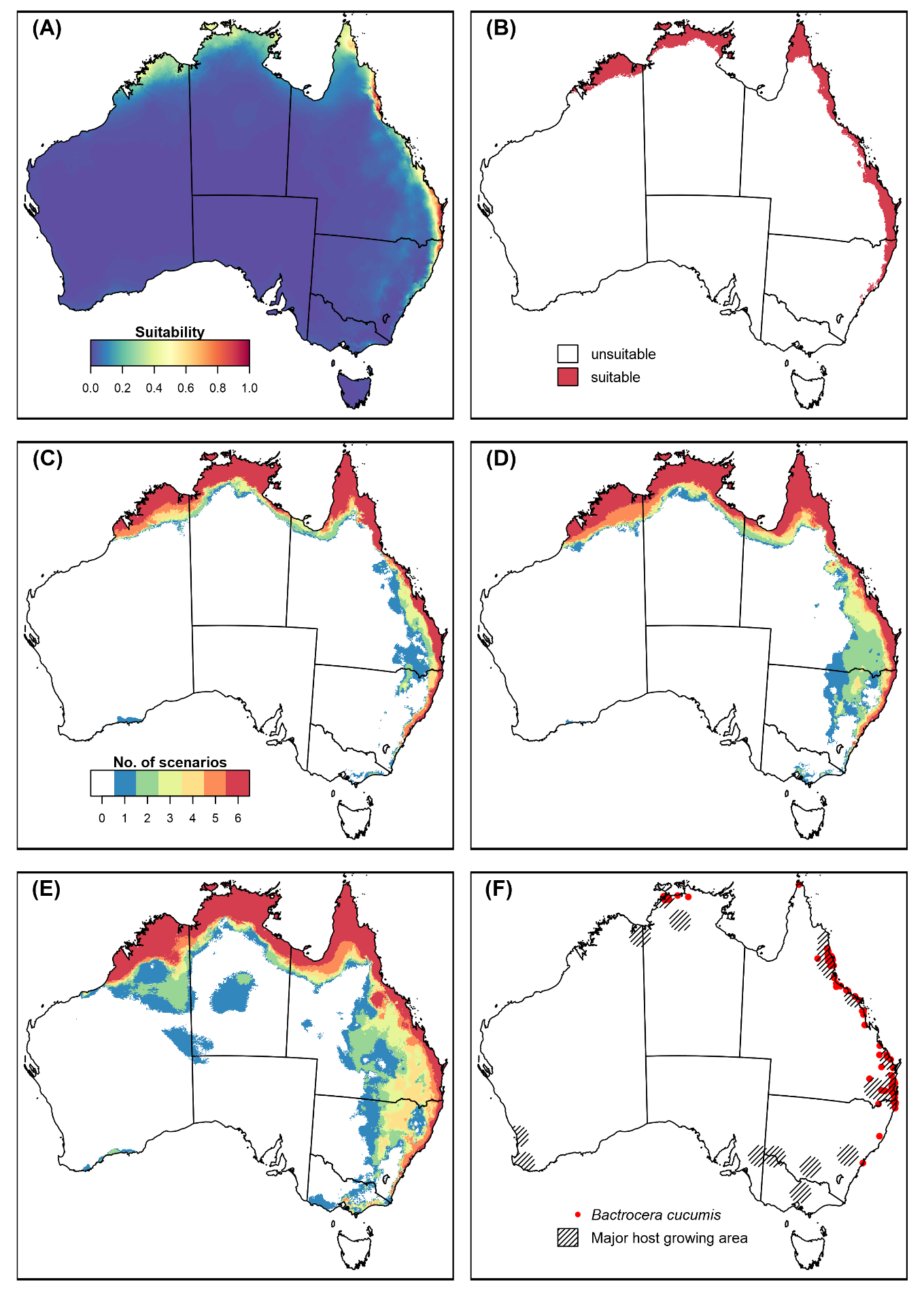


**S12-22 Figs. Climatic habitat suitability for 11 tephritid fruit flies under various future climate scenarios, when novel environments are excluded.** (12) *Bactrocera aquilonis,* (13) *Bactrocera bryoniae,* (14) *Bactrocera frauenfeldi,* (15) *Bactrocera halfordiae,* (16) *Bactrocera jarvisi,* (17) *Bactrocera kraussi,* (18) *Bactrocera musae,* (19) *Bactrocera neohumeralis,* (20) *Bactrocera tryoni,* (21) *Ceratitis capitata,* (22) *Zeugodacus cucumis*. (A) baseline (1960-1990) habitat suitability modelled using Maxent – values close to zero represent areas with low climatic suitability while values closer to one indicate higher climatic suitability; (B) areas considered “suitable” (i.e., with habitat suitability values above the 10th percentile at training presence sites, shown in red); (C, D, E) agreement about the suitability of habitat for the species across six climate scenarios for 2030, 2050 and 2070, respectively; (F) the location of Australian occurrence records of the species, which were used to calibrate models, based on specimens from natural history collections, literature and State Government trapping programs, and major commercial horticultural hosts, according to the Australian Horticulture Statistics Handbook (HSHB; [www.horticulture.com.au](http://www.horticulture.com.au)).

(12) *Bactrocera aquilonis*


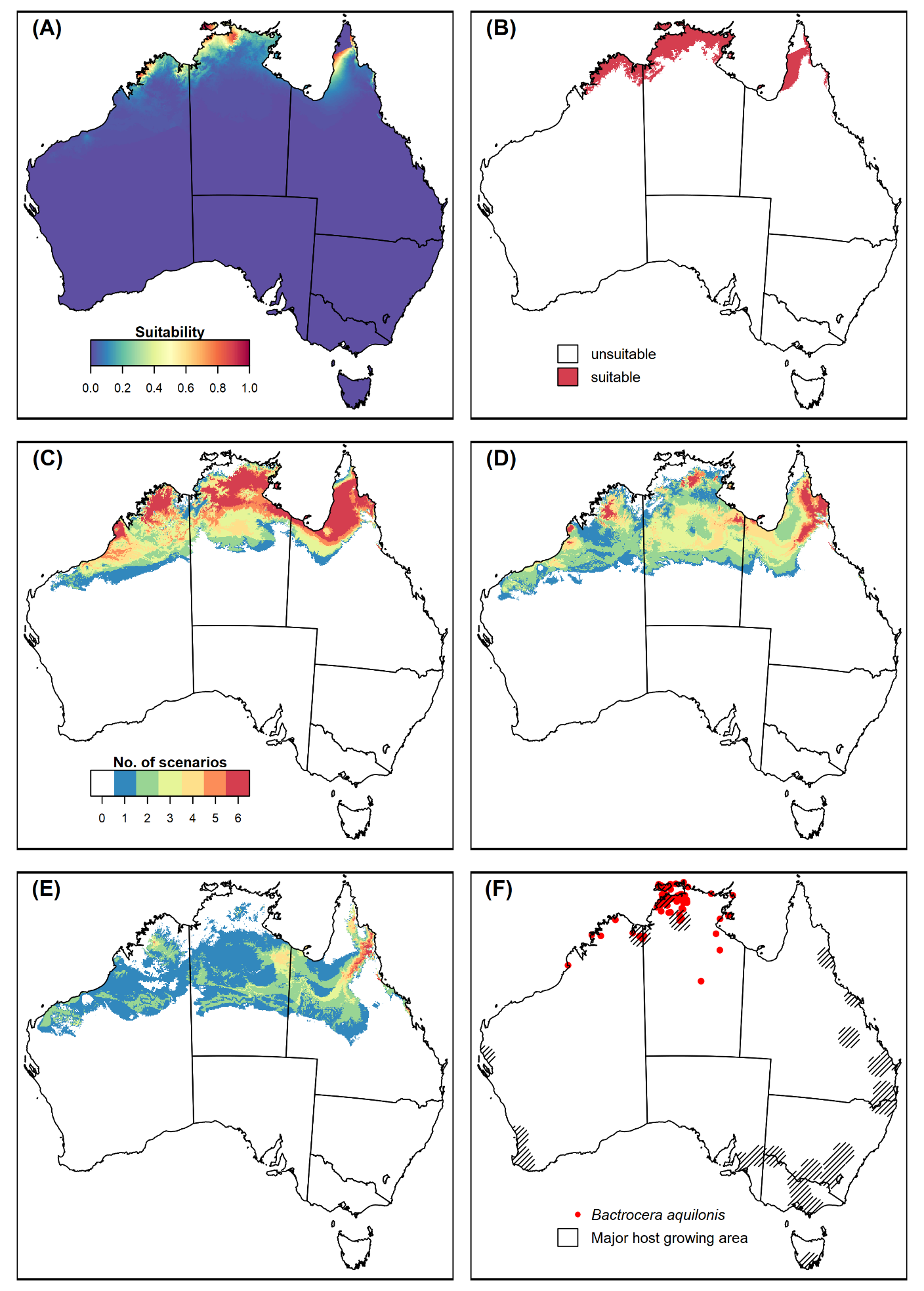


(13) *Bactrocera bryoniae*


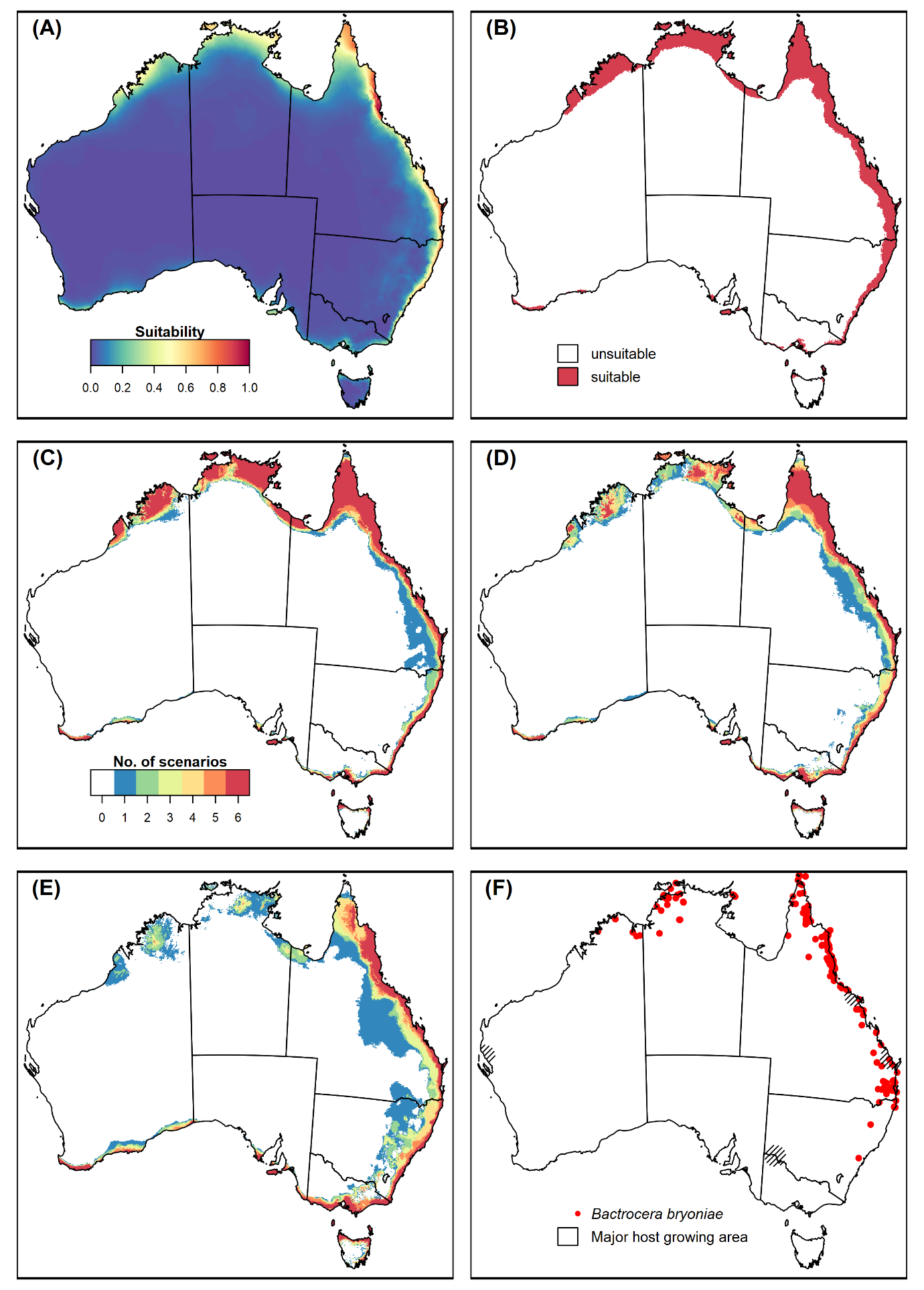


(14) *Bactrocera frauenfeldi*


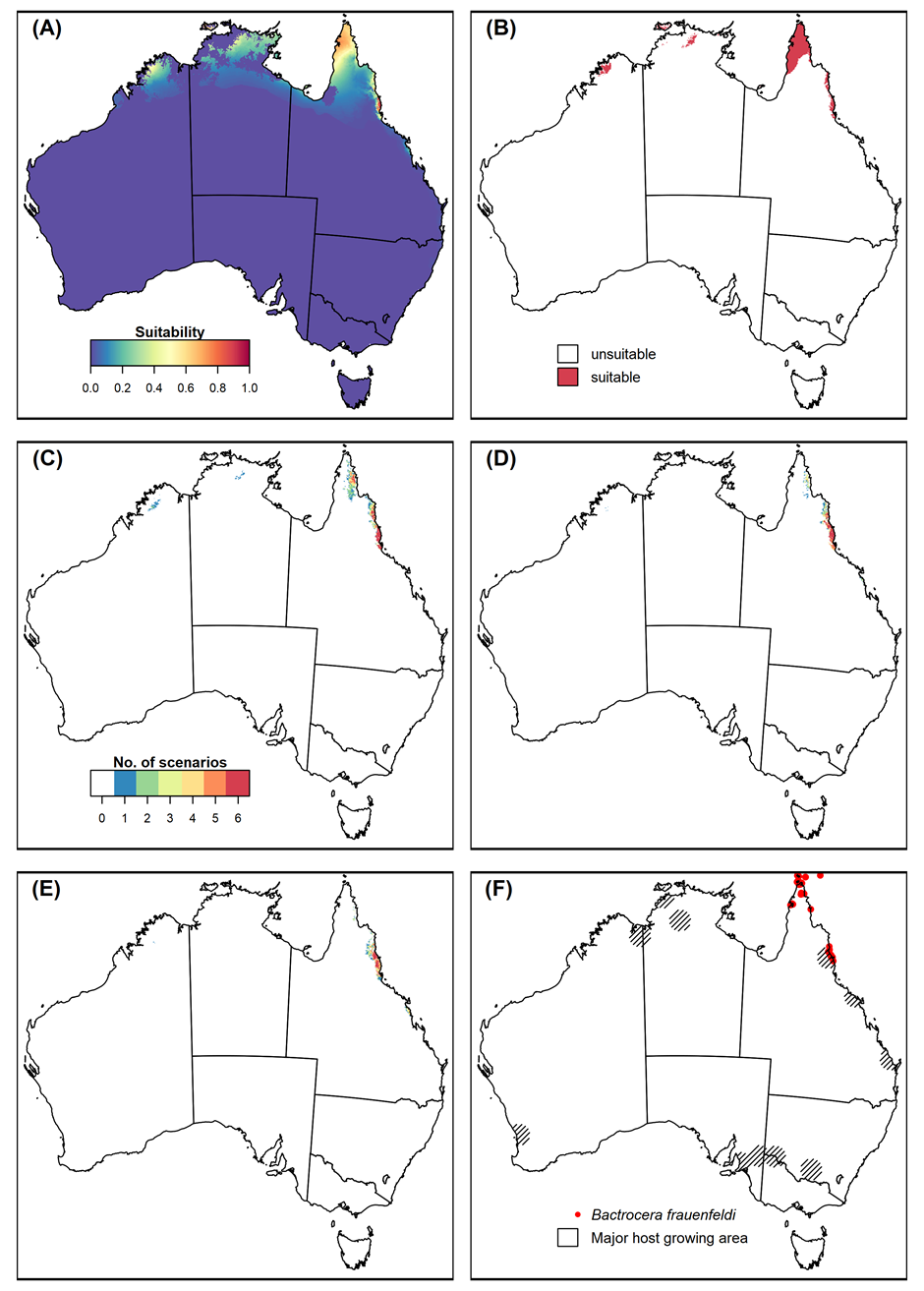


(15) *Bactrocera halfordiae*


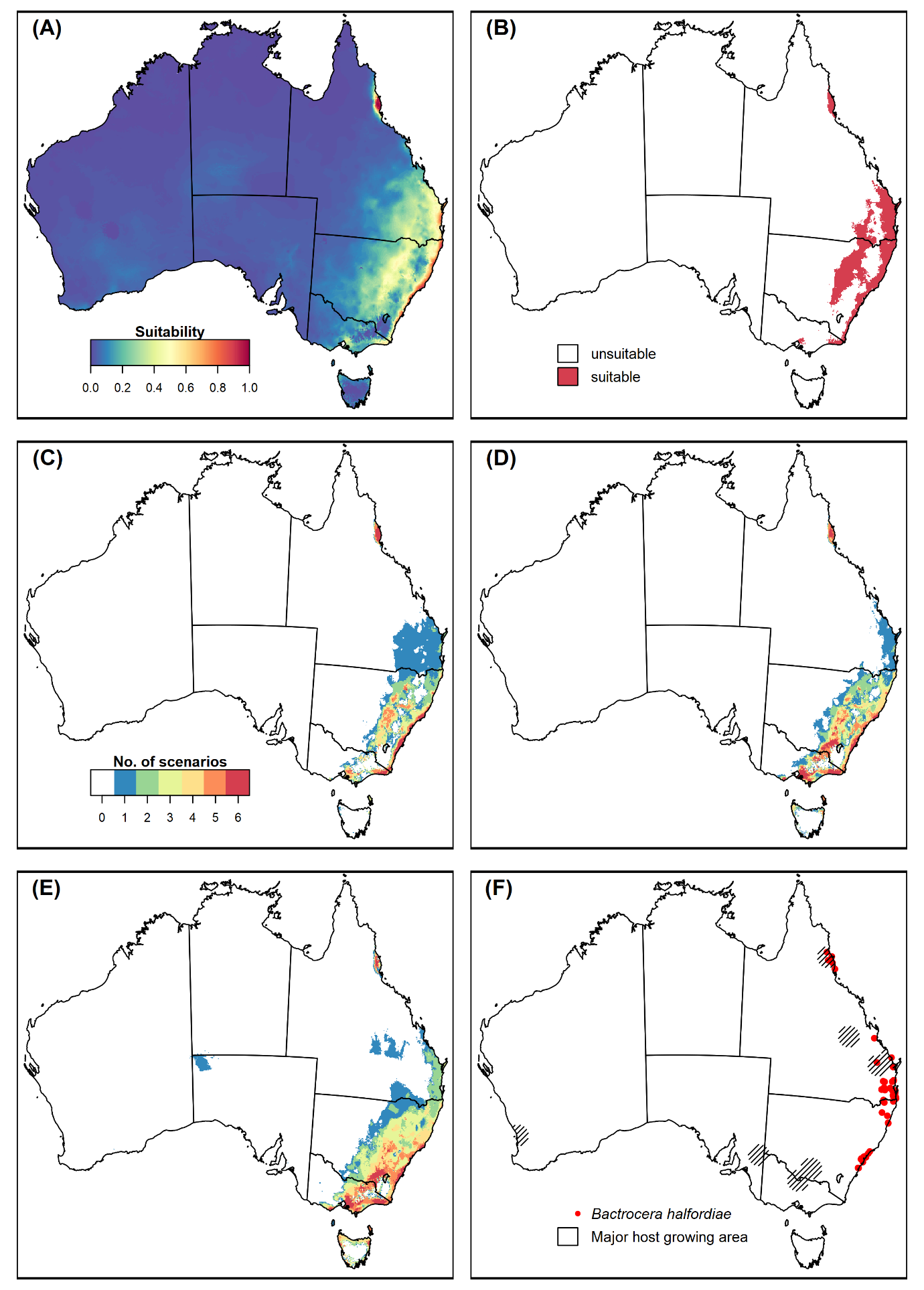


(16) *Bactrocera jarvisi*


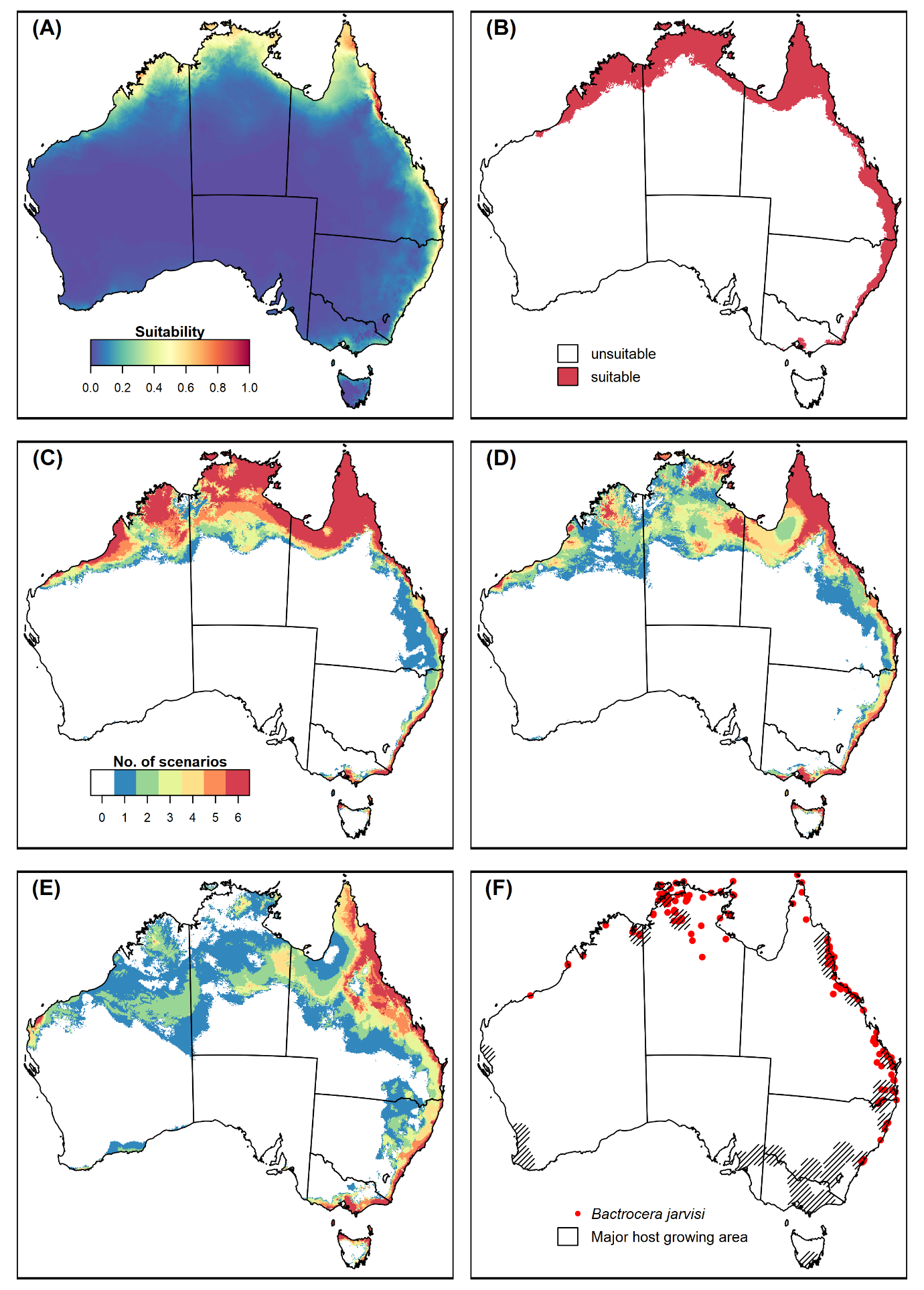


(17) *Bactrocera kraussi*


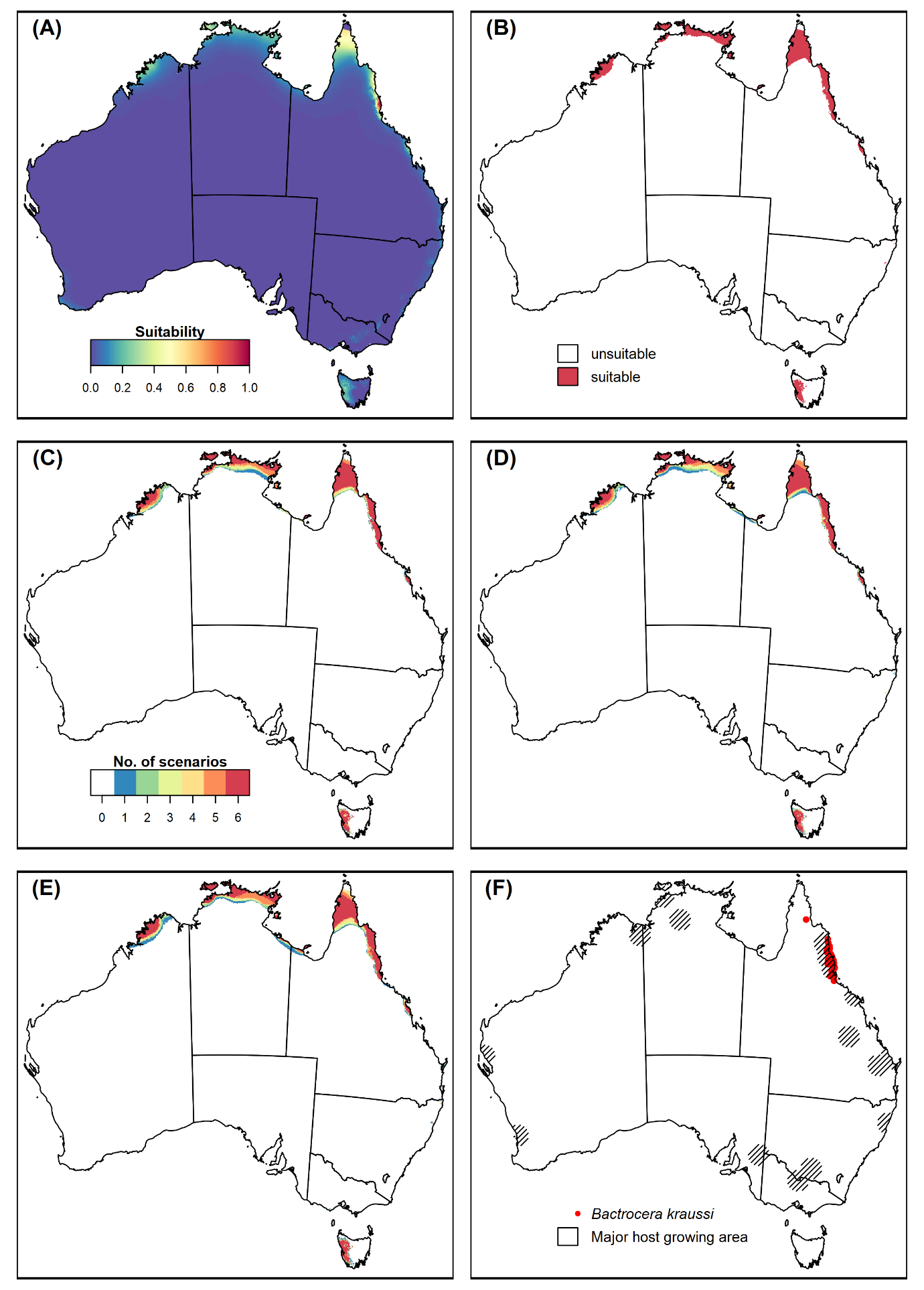


(18) *Bactrocera musae*


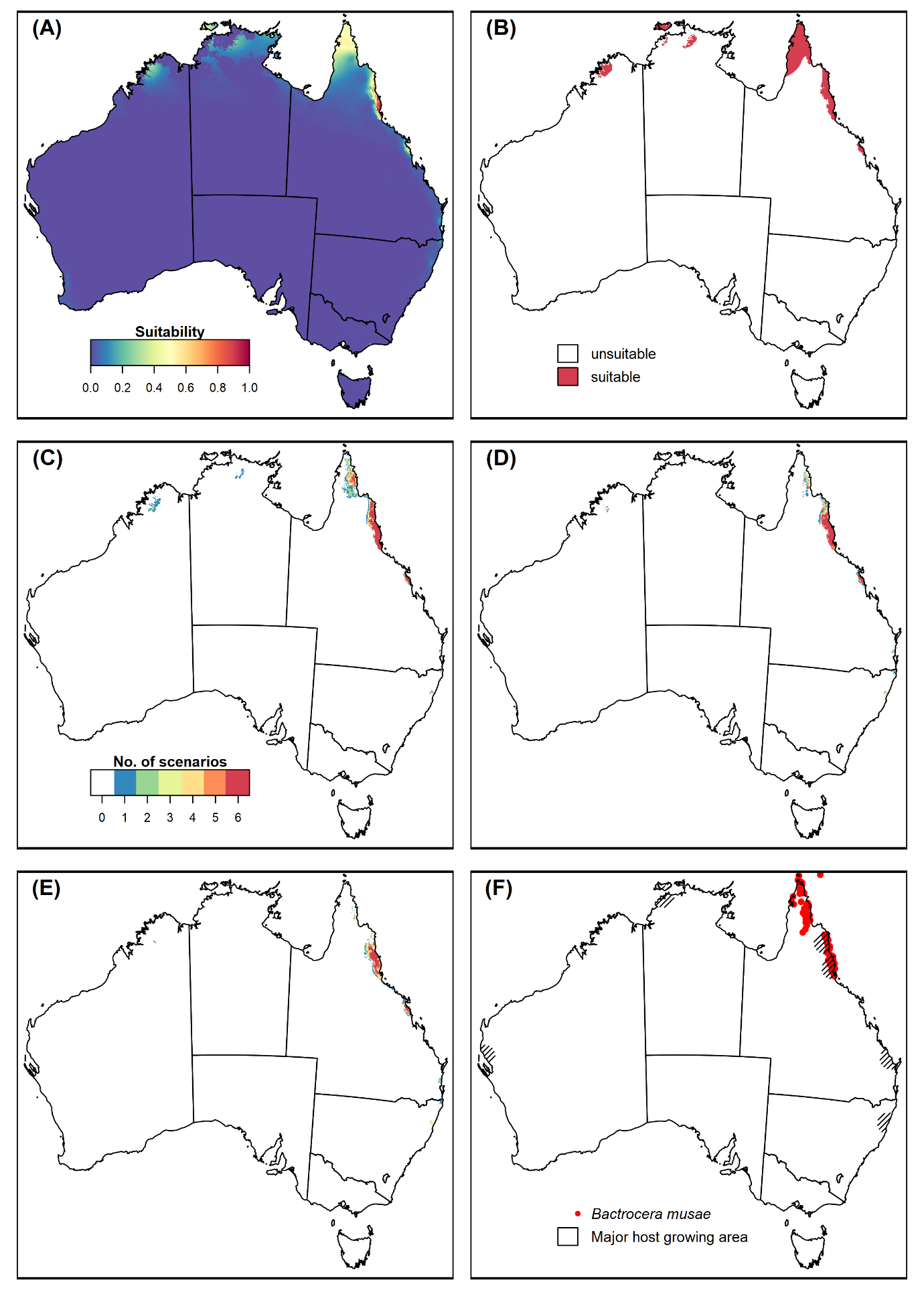


(19) *Bactrocera neohumeralis*


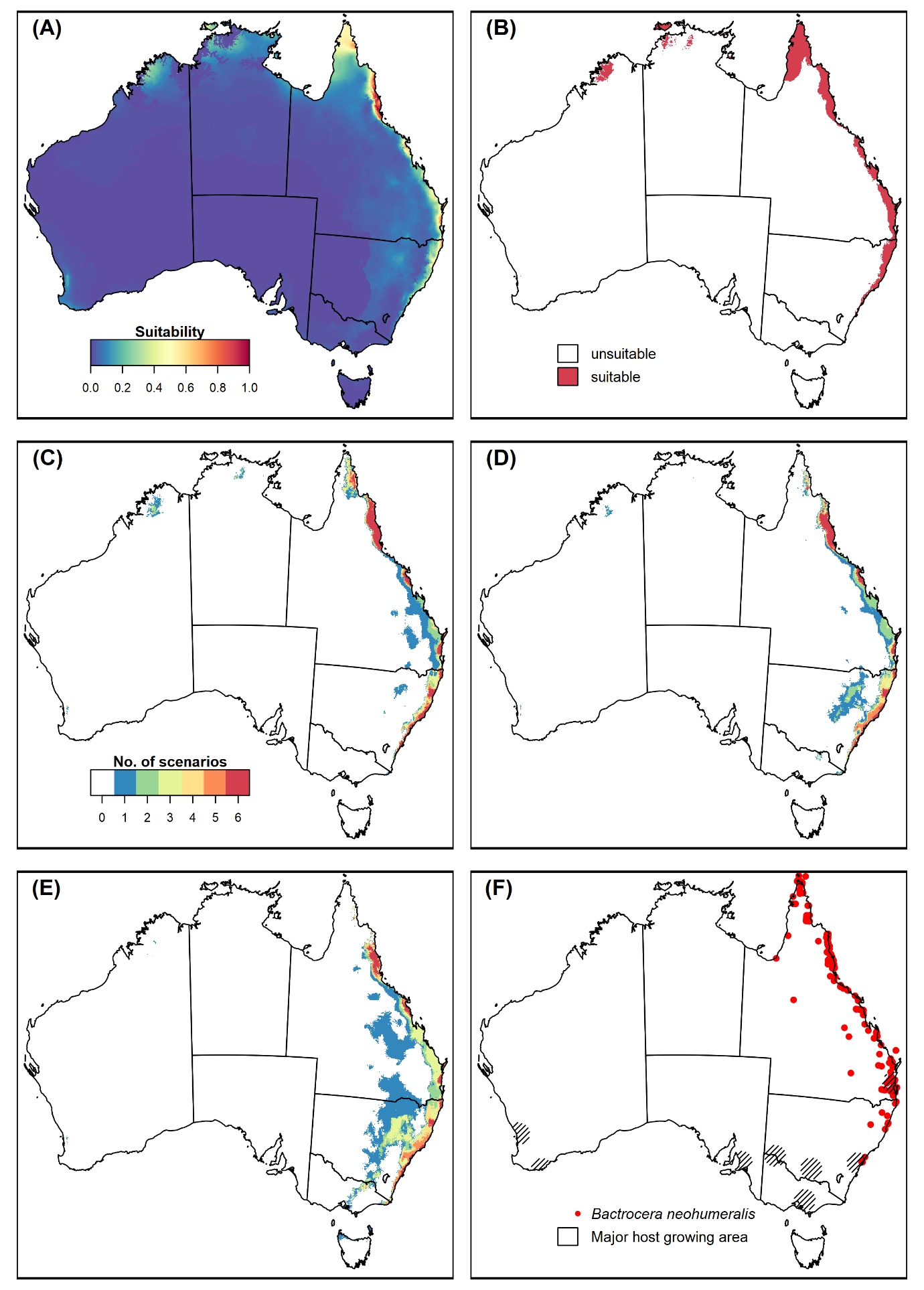


(20) *Bactrocera tryoni*


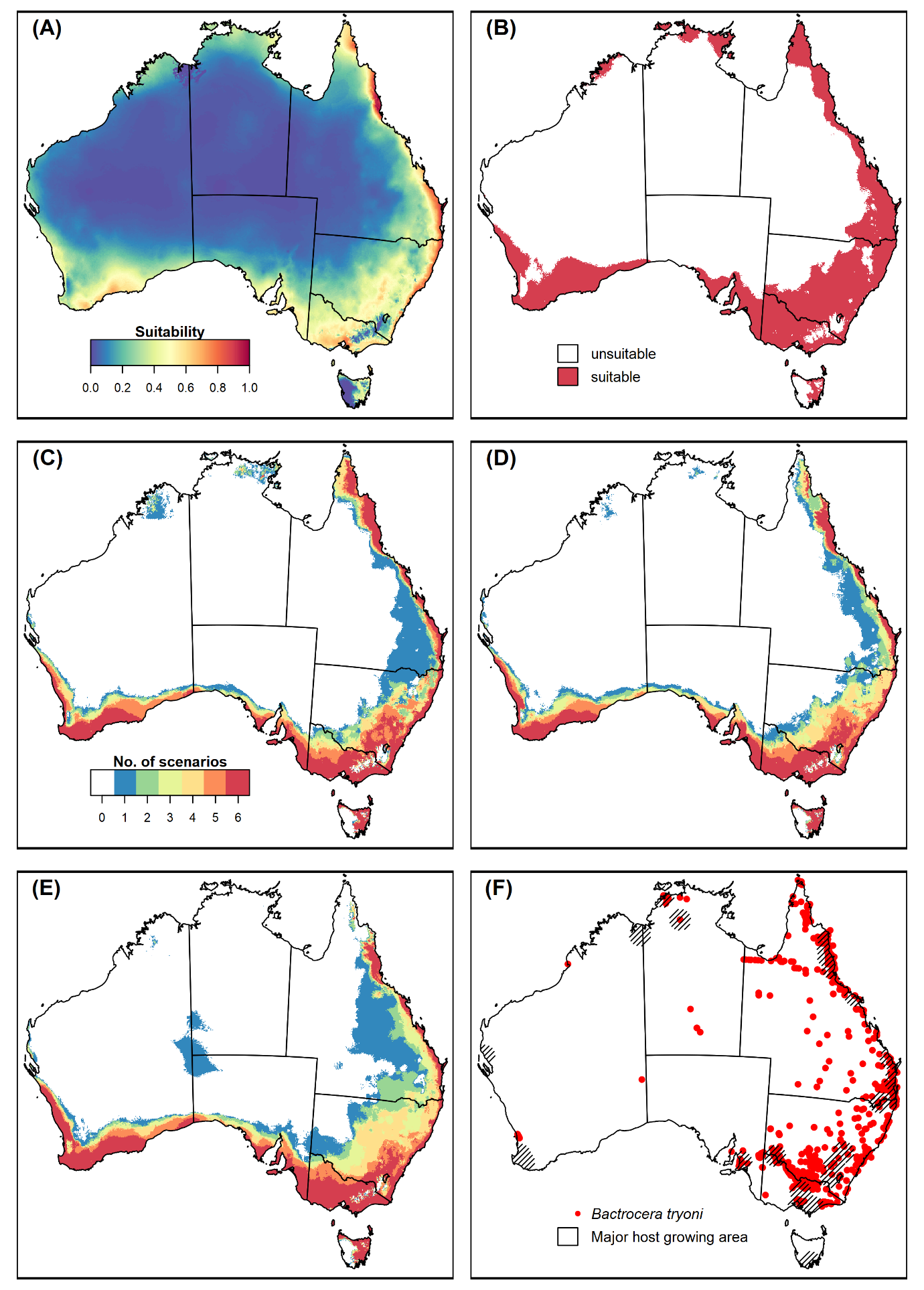


(21) *Ceratitis capitata*


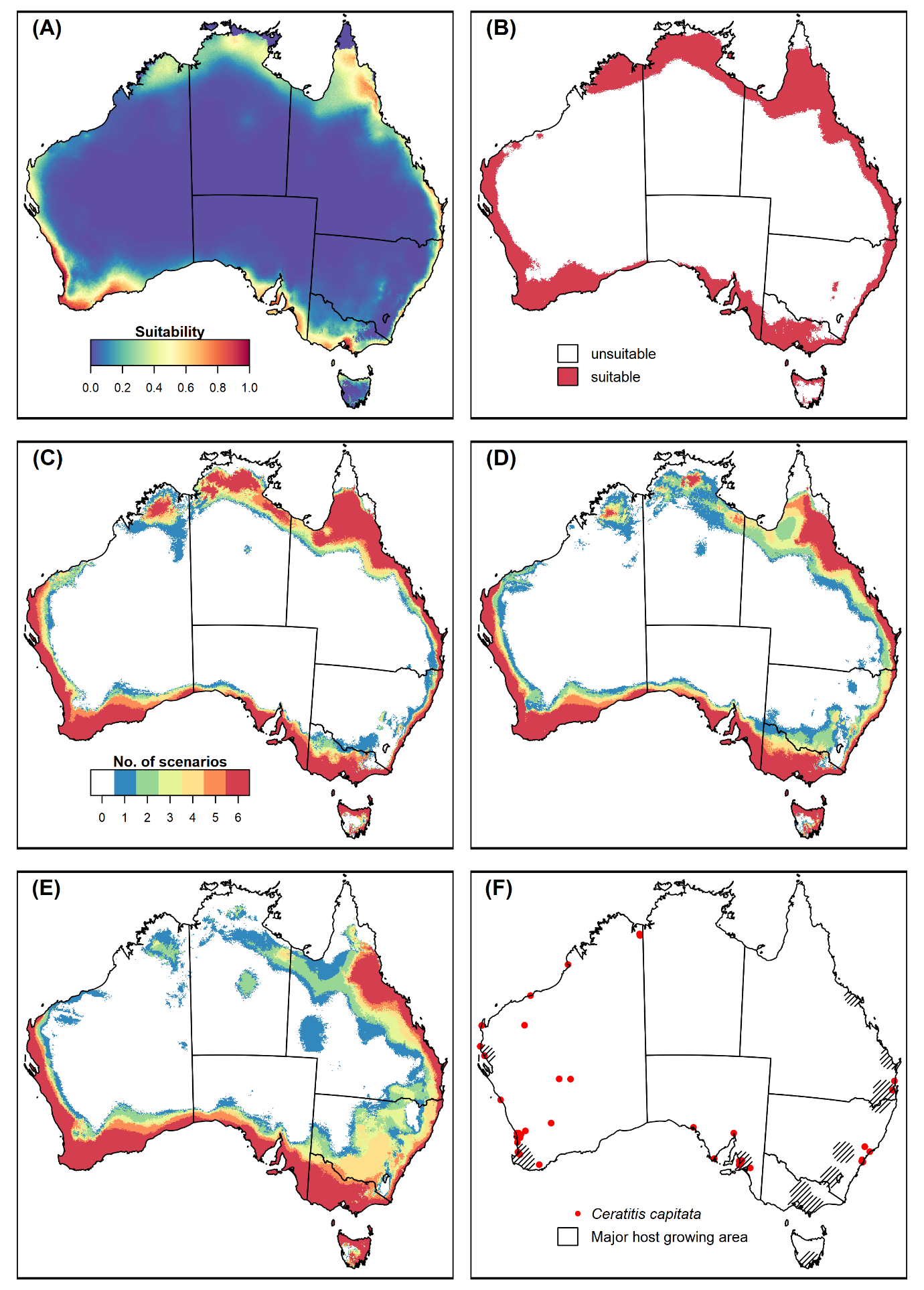


(22) *Zeugodacus cucumis*


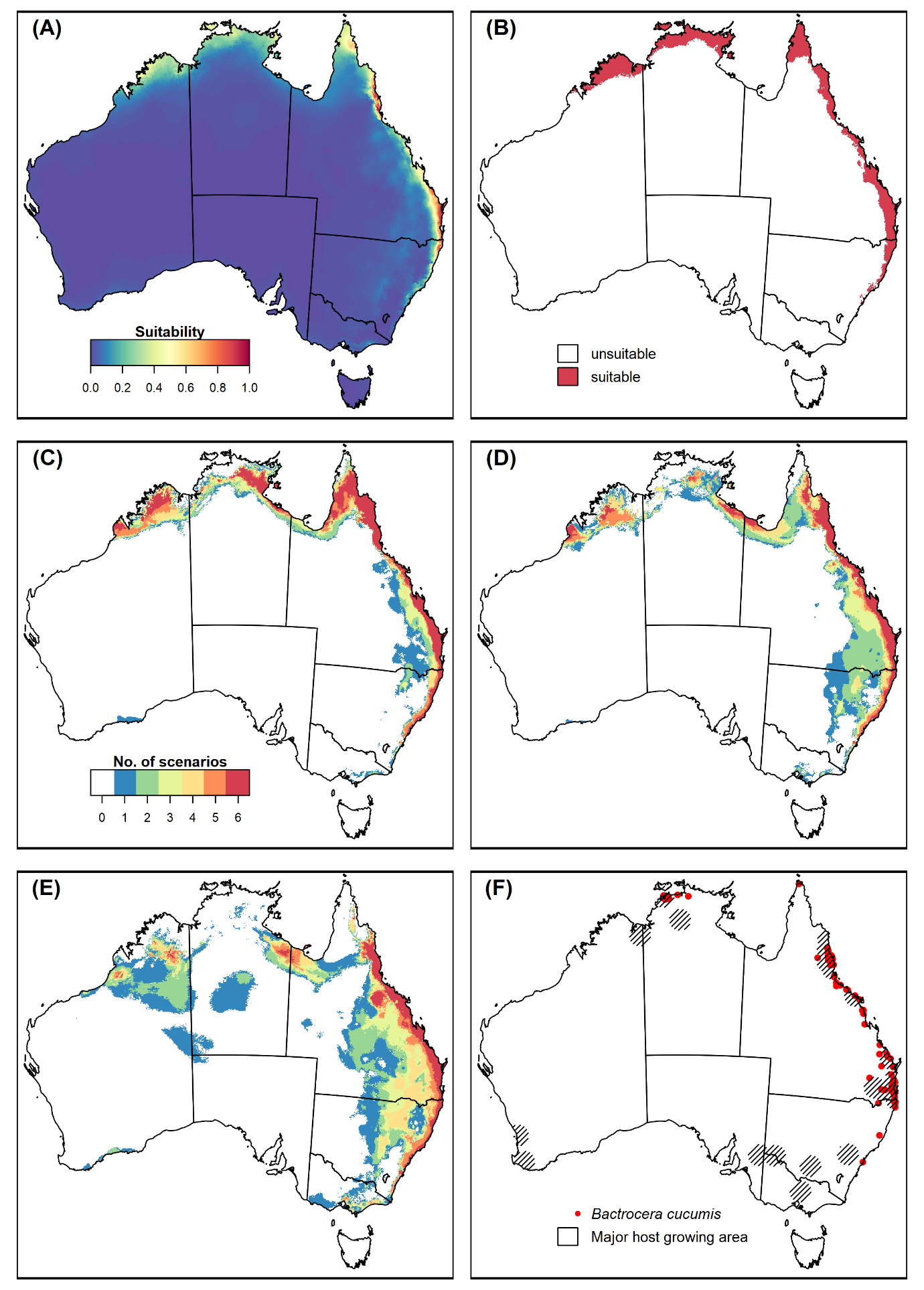


**S23-33 Figs. Projected changes of suitable habitat for all 11 fruit fly species, under six future climate scenarios, relative to the baseline period (1960-1990).** (23) *Bactrocera aquilonis,* (24) *Bactrocera bryoniae,* (25) *Bactrocera frauenfeldi,* (26) *Bactrocera halfordiae,* (27) *Bactrocera jarvisi,* (28) *Bactrocera kraussi,* (29) *Bactrocera musae,* (30) *Bactrocera neohumeralis,* (31) *Bactrocera tryoni,* (32) *Ceratitis capitata,* (33) *Zeugodacus cucumis.* Colours indicate projected changes of suitable habitat of species under future climate scenarios, where blue colour indicates suitability with novel environments, red colour indicates suitability without novel environments and gray colour indicates unsuitability.

(23) *Bactrocera aquilonis*


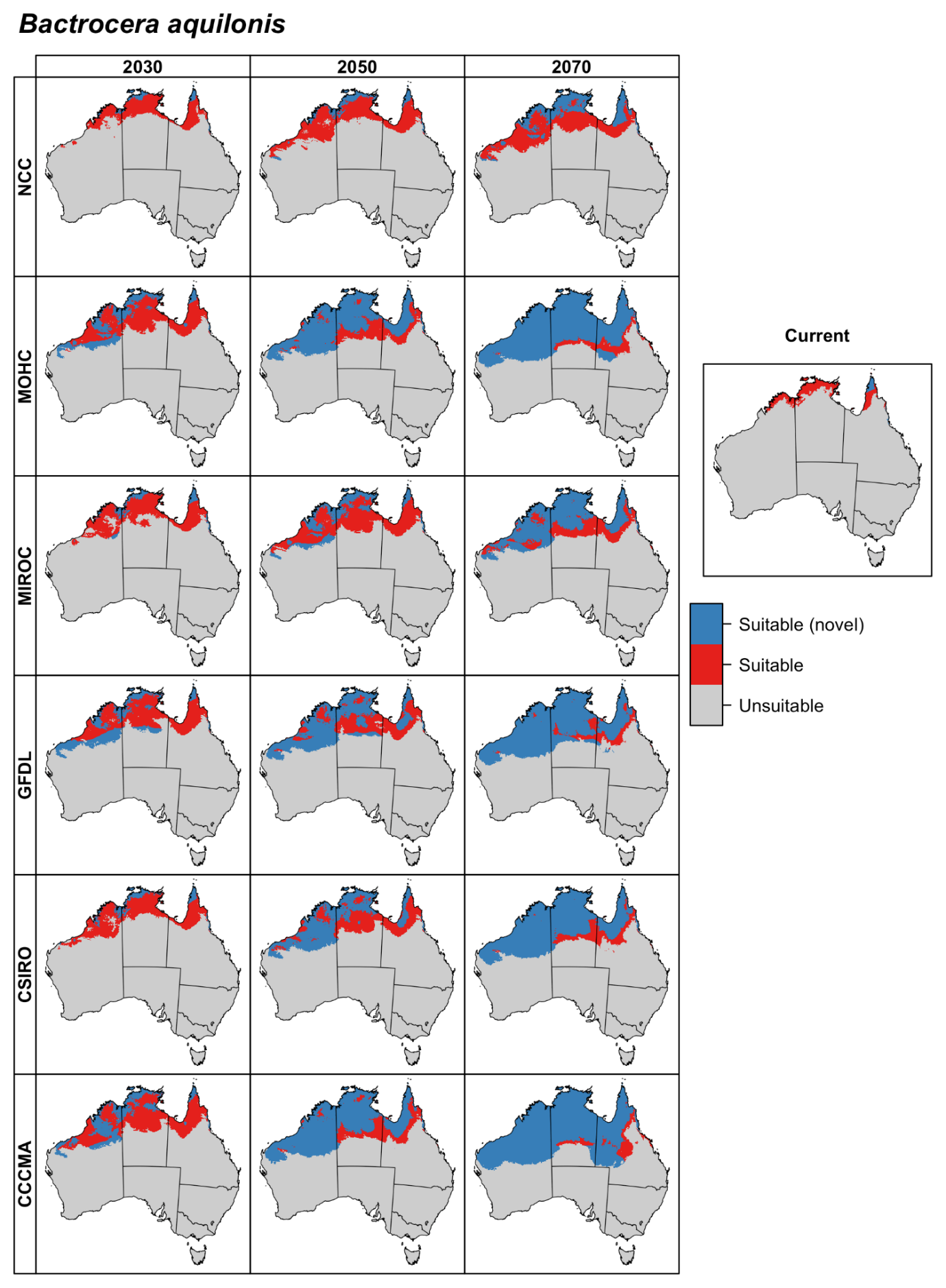


(24) *Bactrocera bryoniae*


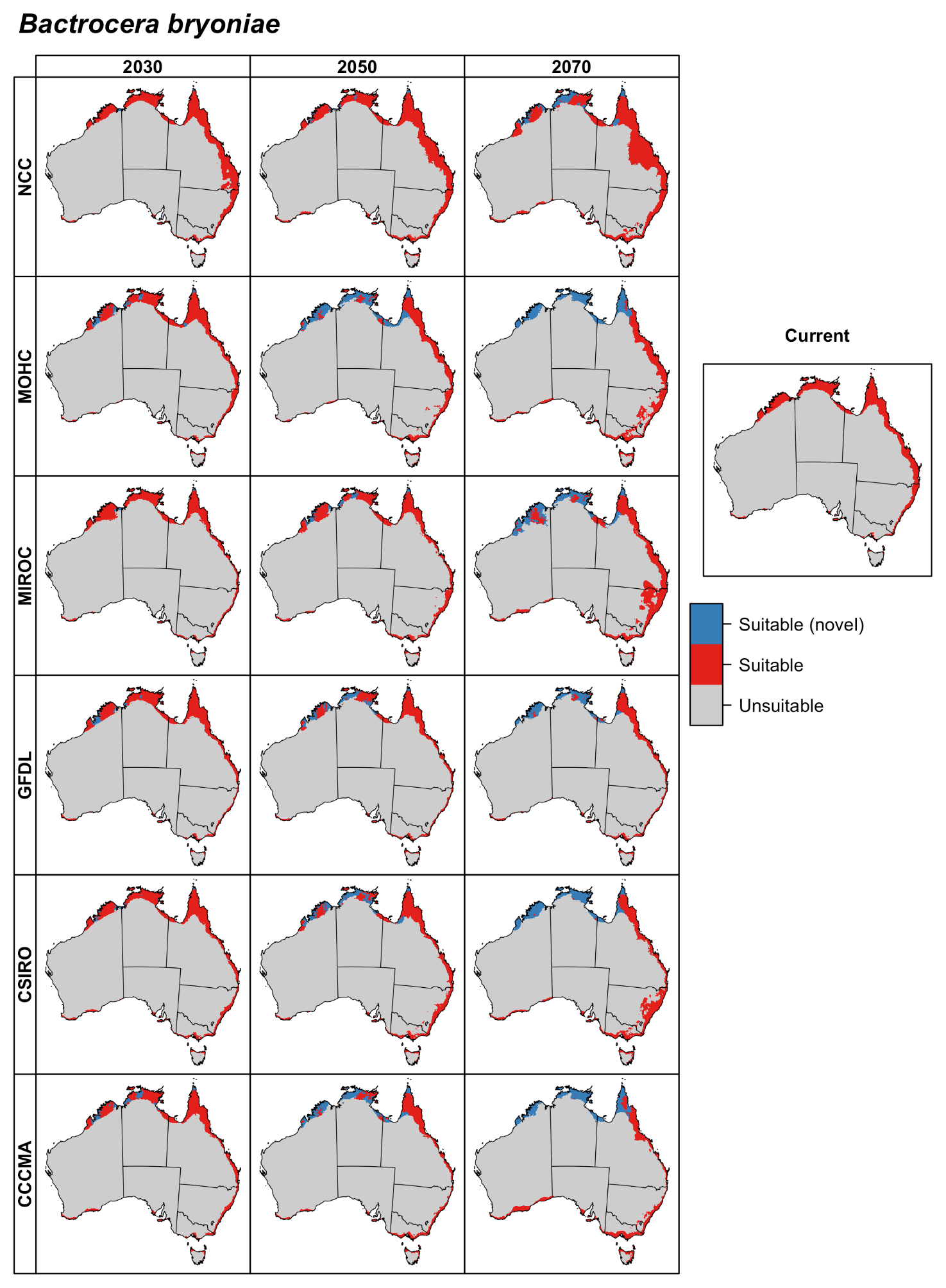


(25) *Bactrocera frauenfeldi*


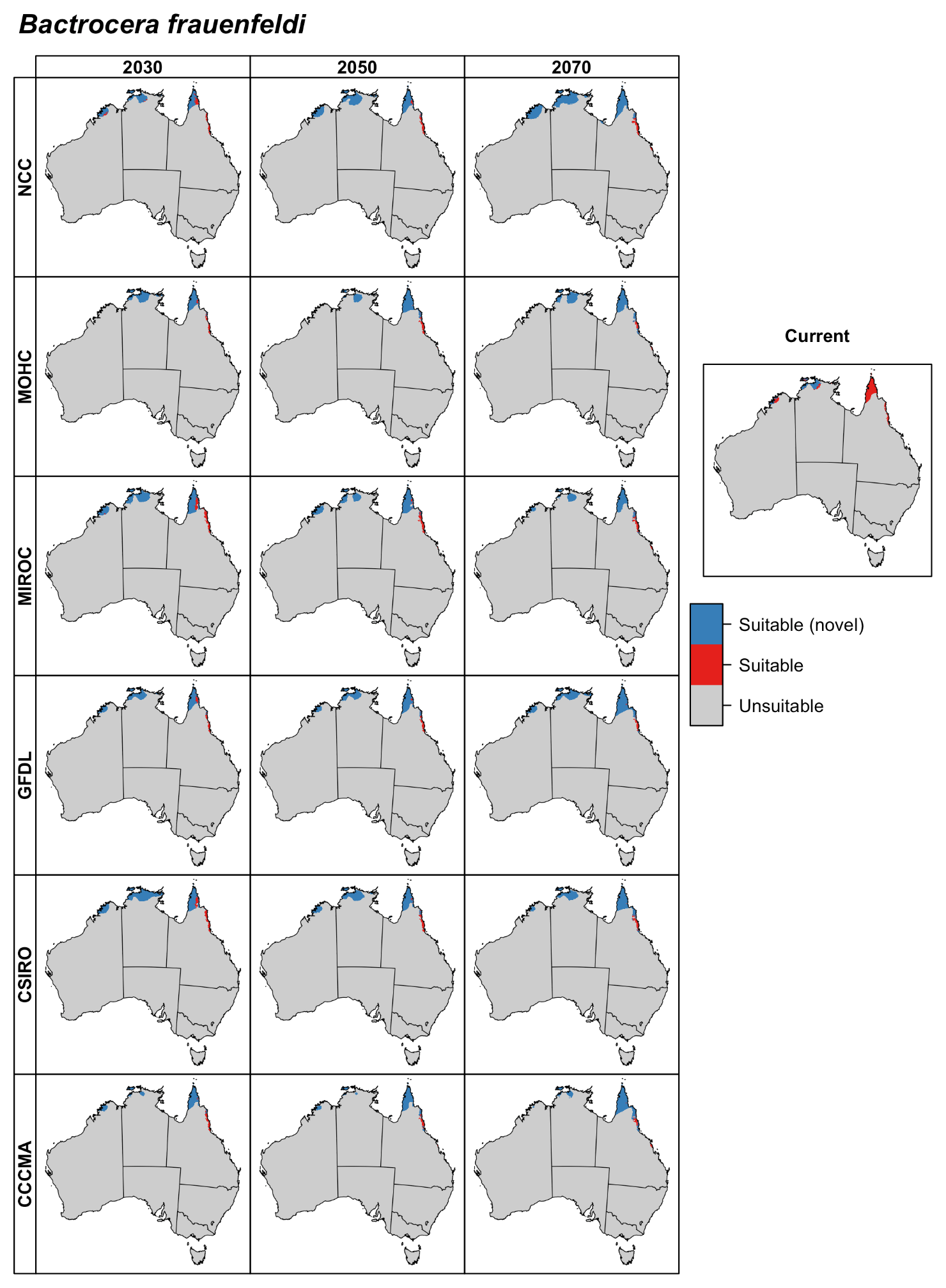


(26) *Bactrocera halfordiae*


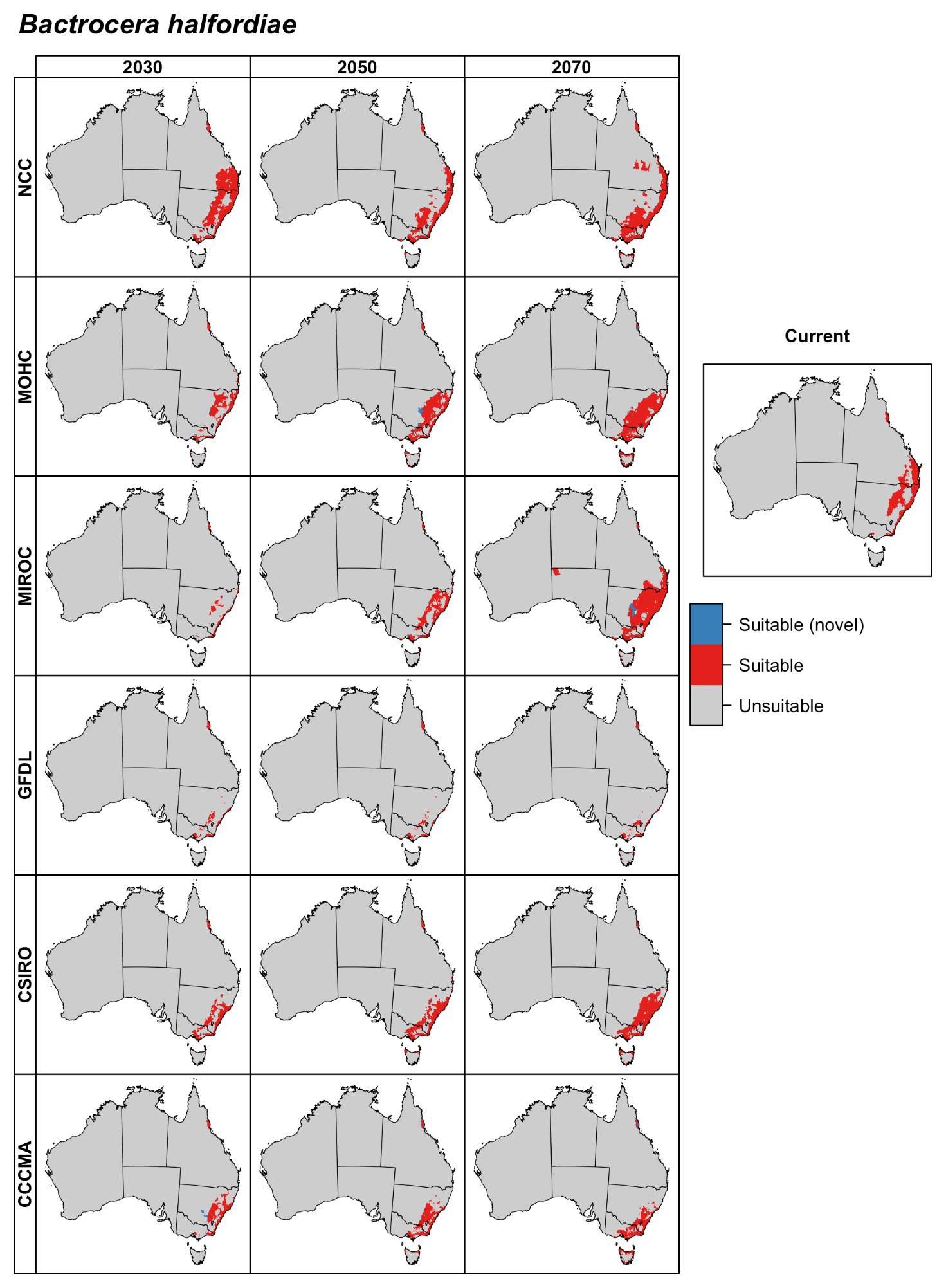


(27) *Bactrocera jarvisi*


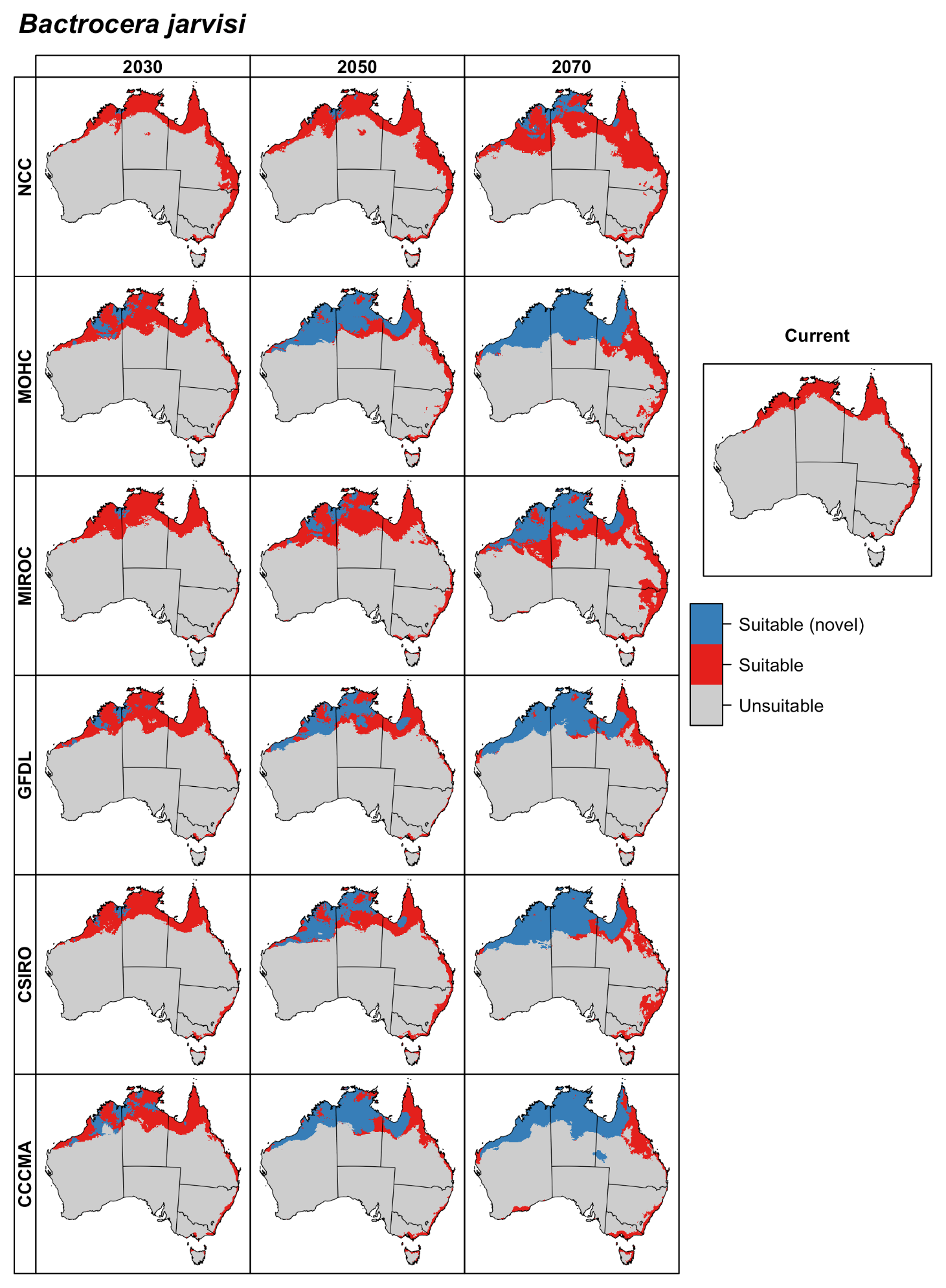


(28) *Bactrocera kraussi*


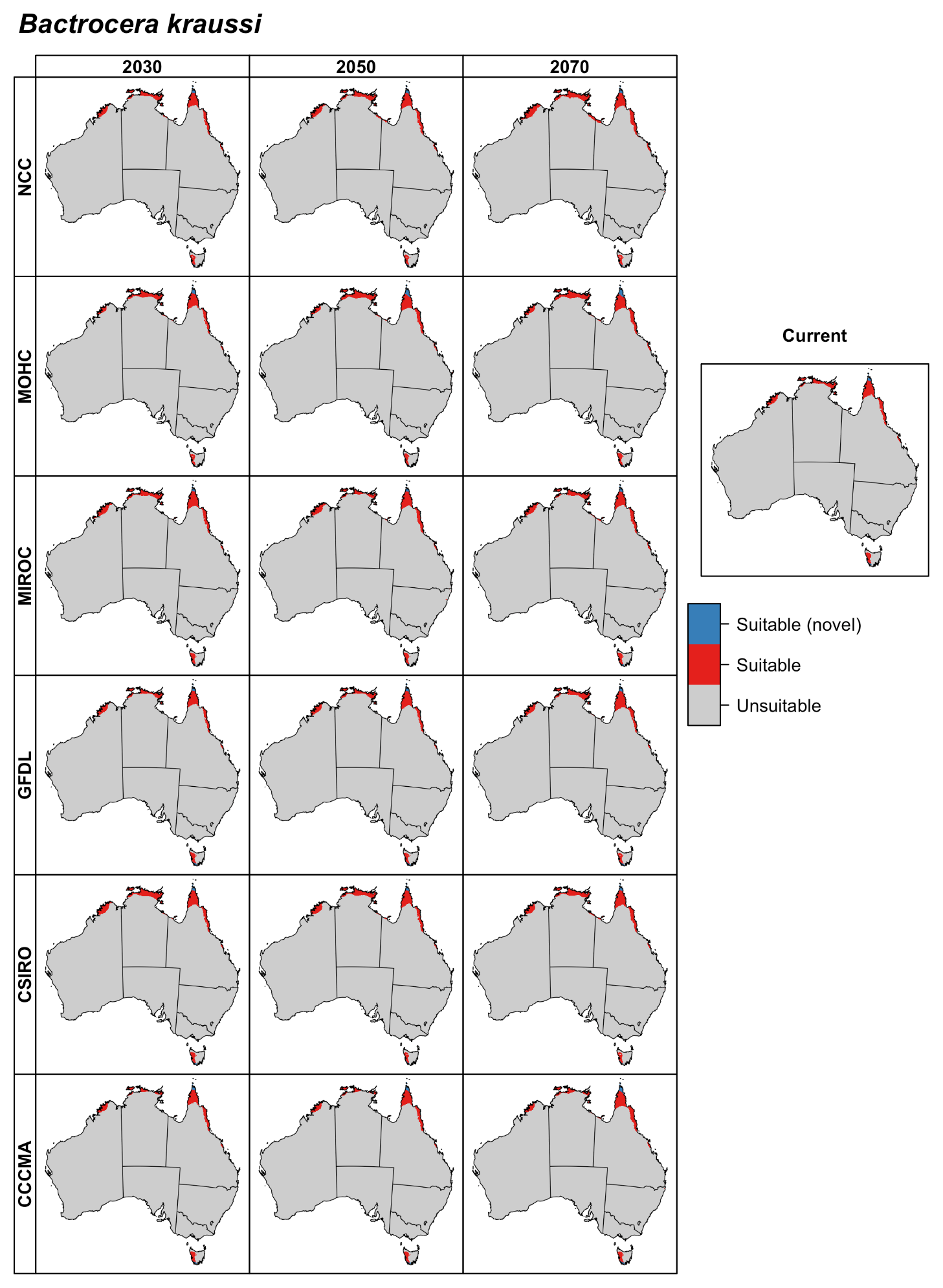


(29) *Bactrocera musae*


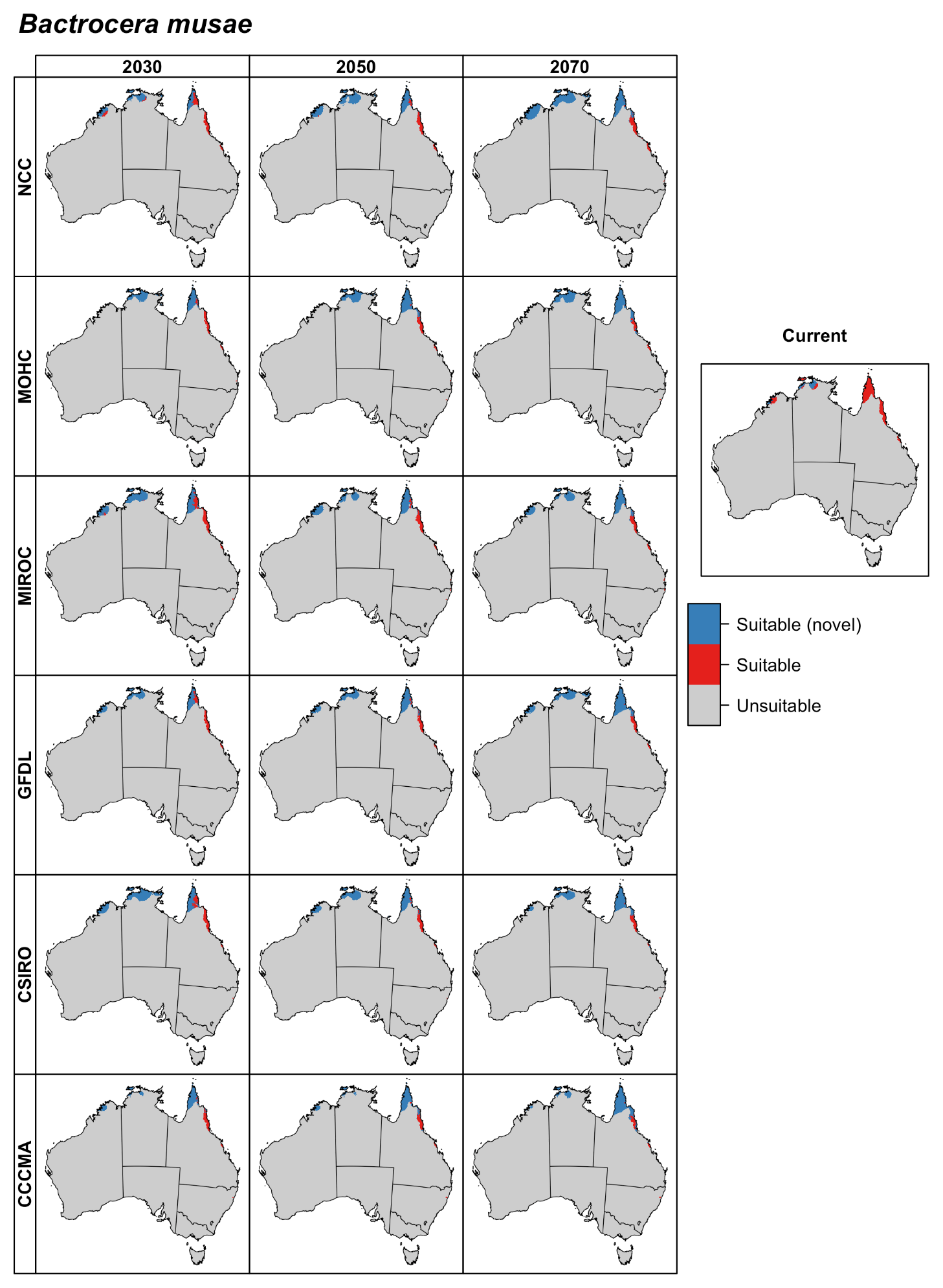


(30) *Bactrocera neohumeralis*


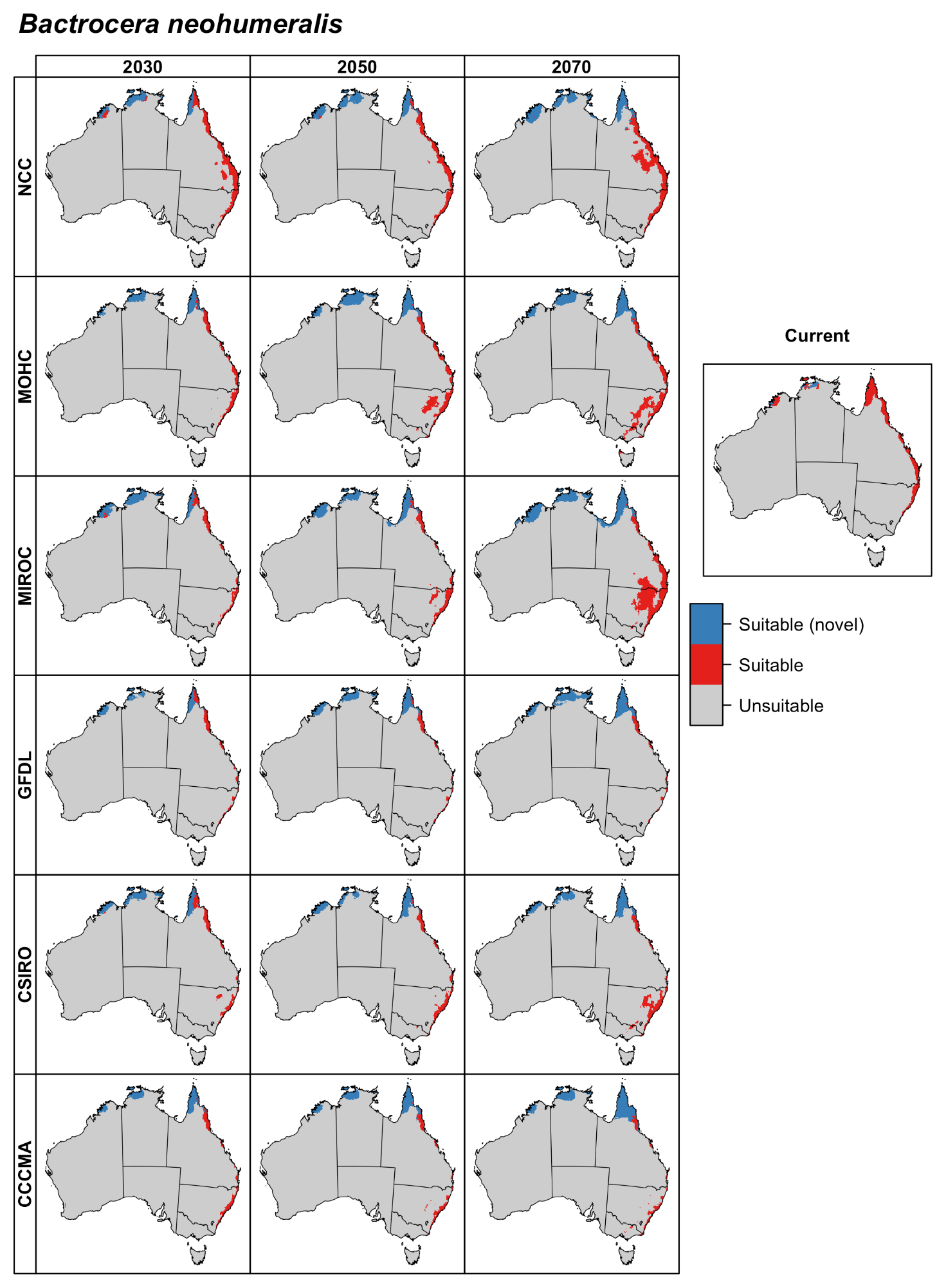


(31) *Bactrocera tryoni*


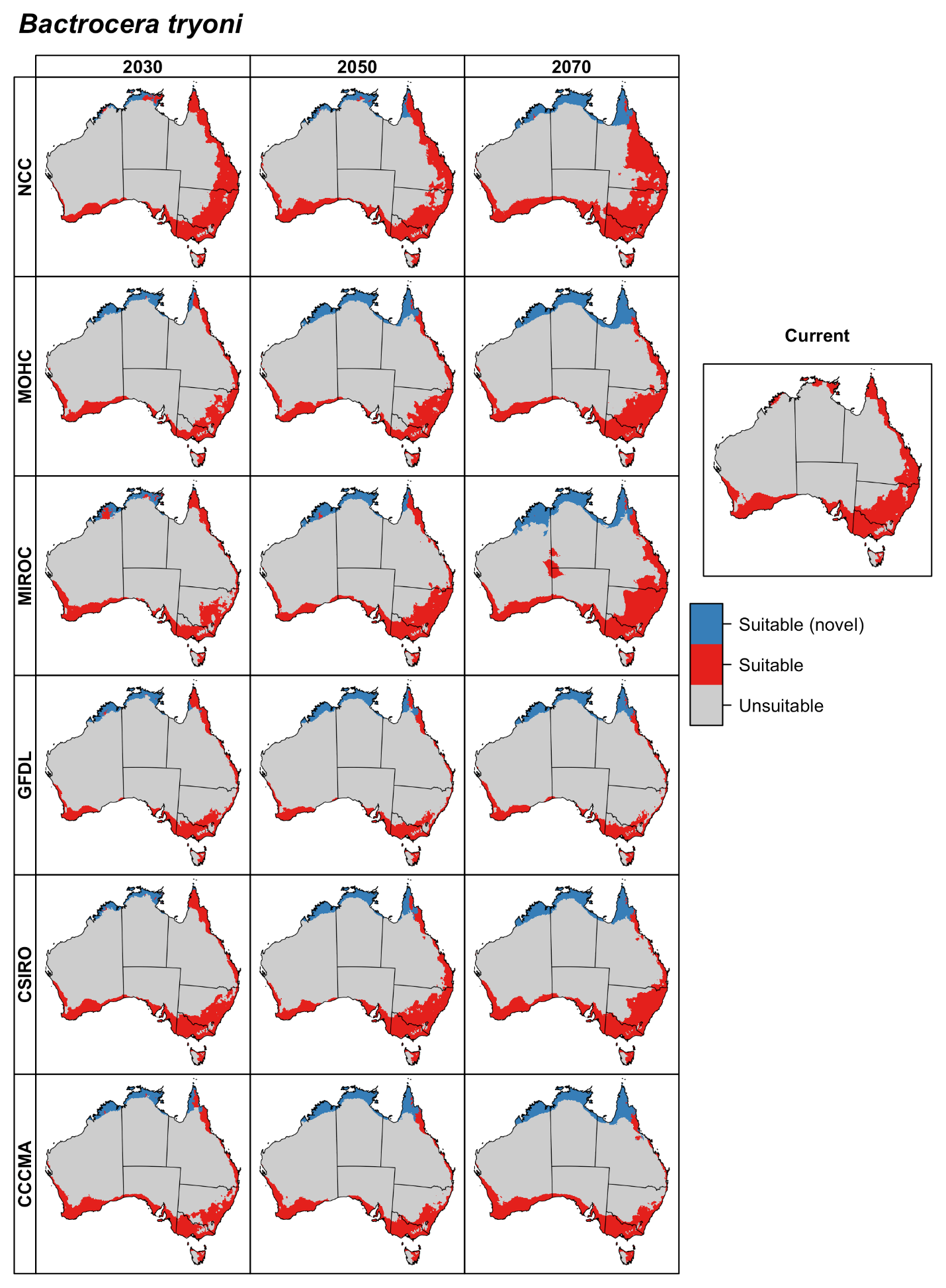


(32) *Ceratitis capitata*


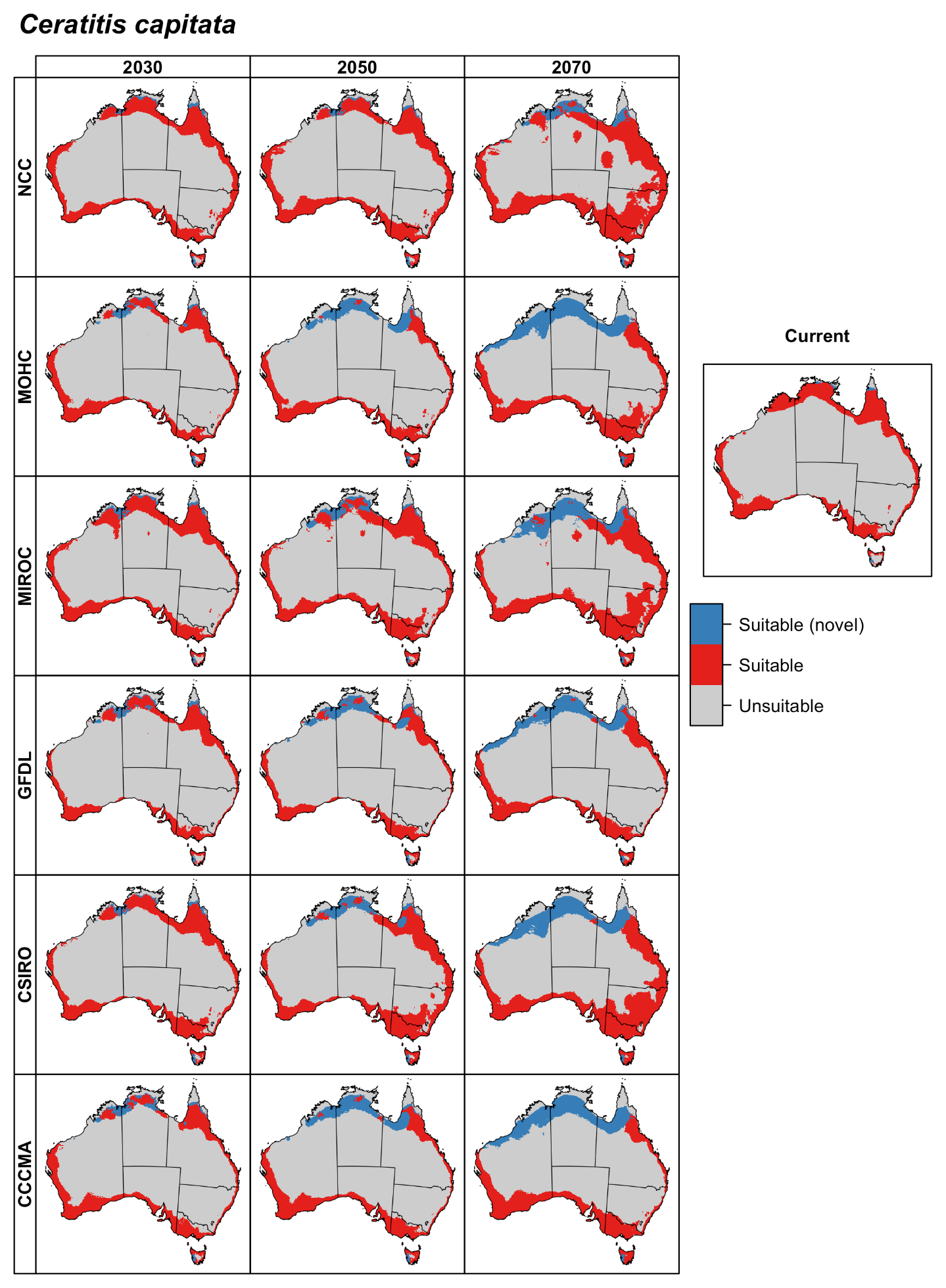


(33) *Zeugodacus cucumis*


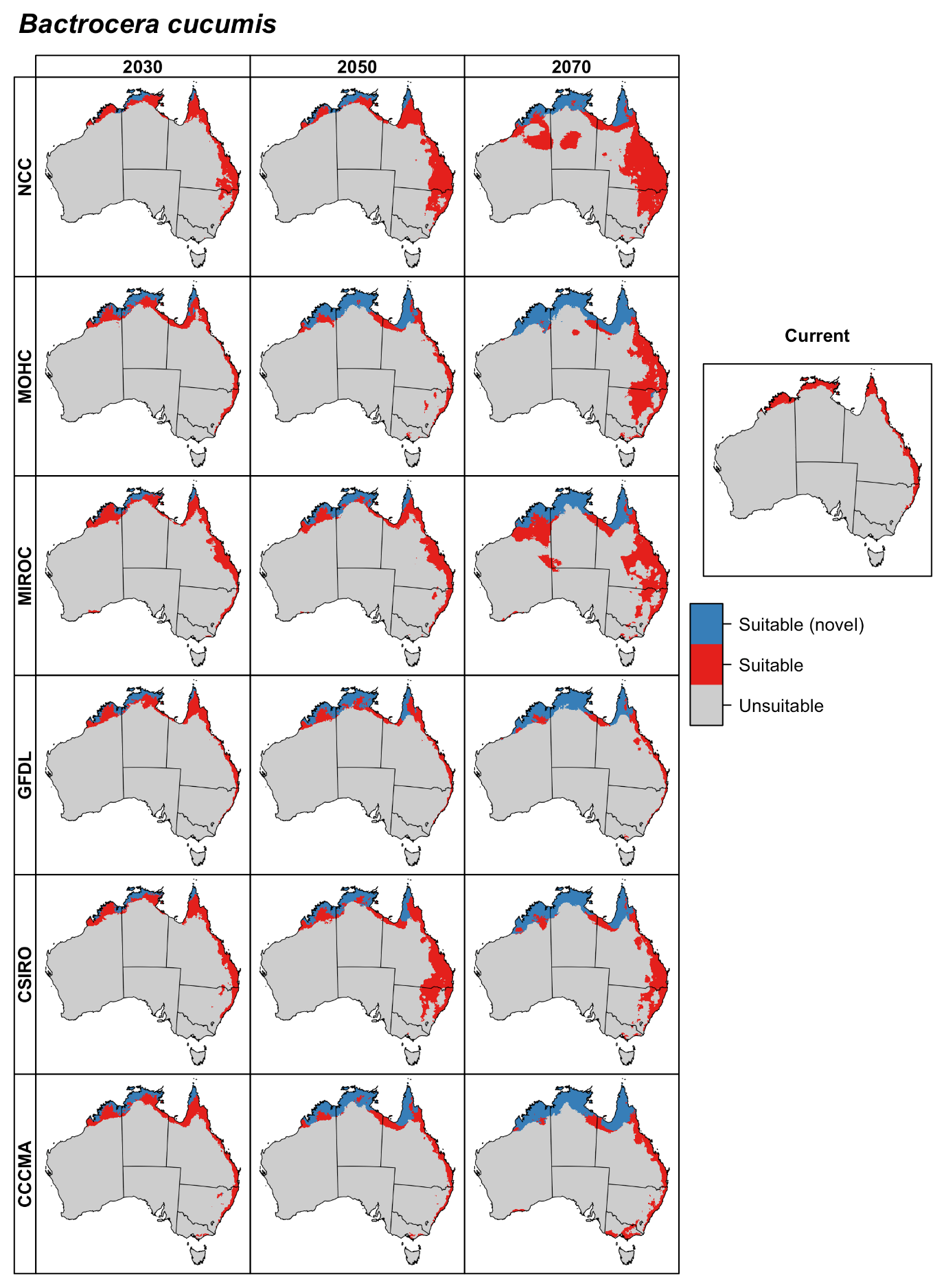


**S1 Table. Model performance and bioclimatic variables used to investigate the suitability of habitat for tephritid fruit fly species.**

| Species | AUC | SD | HPI (Highest Permutation Importance, %) |
| --- | --- | --- | --- |
| *Bactrocera aquilonis* | 0.896 | 0.02 | BIO16: 68.9, BIO01: 28.9, BIO11: 2.5 |
| *Bactrocera bryoniae* | 0.853 | 0.03 | BIO01: 24.3, BIO06: 6.1, BIO07: 42.2, BIO14: 27.4 |
| *Bactrocera frauenfeldi* | 0.815 | 0.05 | BIO01: 21.7, BIO13: 3.2, BIO16: 75.4 |
| *Bactrocera halfordiae* | 0.846 | 0.03 | BIO01: 32.3, BIO03: 0.9, BIO14: 66.8 |
| *Bactrocera jarvisi* | 0.817 | 0.04 | BIO01: 37.9, BIO07: 24.7, BIO14: 37.2 |
| *Bactrocera kraussi* | 0.904 | 0.05 | BIO02: 24.7, BIO13: 0.1, BIO16: 75.2 |
| *Bactrocera musae* | 0.861 | 0.02 | BIO01: 2.5, BIO13: 4.4, BIO14: 14.4, BIO16:78.7 |
| *Bactrocera neohumeralis* | 0.907 | 0.02 | BIO01: 21.9, BIO03: 1.3, BIO13: 47.4, BIO14: 29.3 |
| *Bactrocera tryoni* | 0.841 | 0.01 | BIO01: 33.1, BIO06:32.4, BIO07: 3.93, BIO14: 26.7, BIO19: 3.9 |
| *Ceratitis capitata* | 0.885 | 0.02 | BIO01: 47.1, BIO06: 46.1, BIO07: 2.6, BIO14: 3.9, BIO17: 0.2 |
| *Zeugodacus cucumis* | 0.886 | 0.03 | BIO02: 2.4, BIO11: 36.2, BIO14: 7.1, BIO17: 54.3 |

AUC value indicates the area under the receiver operating characteristic curve (average of 5 cross-validated replicates), which was used to evaluate model performance; SD (standard deviation); and HPI (highest permutation importance, %) of bioclimatic variables contributing to the model where BIO01: annual mean temperature, BIO02: mean diurnal range; BIO03: isothermality; BIO06: minimum temperature of the coldest month; BIO07: temperature annual range; BIO11: mean temperature of the coldest quarter; BIO13: precipitation of the wettest month; BIO14: precipitation of the driest month;  BIO16: precipitation of the wettest quarter; BIO17: precipitation of the driest quarter and BIO19: precipitation of the coldest quarter.

**S2 Table.** **Projected changes in the area of suitable habitat for all 11 fruit fly species, under six future climate scenarios, relative to the baseline period (1960-1990).** (1) *Bactrocera aquilonis* (2) *Bactrocera bryoniae* (3) *Bactrocera frauenfeldi* (4) *Bactrocera halfordiae* (5) *Bactrocera jarvisi* (6) *Bactrocera kraussi* (7) *Bactrocera musae* (8) *Bactrocera neohumeralis* (9) *Bactrocera tryoni* (10) *Ceratitis capitata* (11) *Zeugodacus cucumis*. For each species, the first column indicates the GCM (Global Climate Model) for three time periods 2030, 2050 and 2070. Other columns: % Lost refers to the percentage of suitable habitat (baseline period) projected to become unsuitable in the future; % Gained refers to the percentage of future suitable habitat that is in areas unsuitable in the baseline period; Range Change refers to the change (%) between the size of baseline and future suitable habitat (positive numbers indicate an increase in range size, negative numbers indicate a decrease).

(1) *Bactrocera aquilonis:*

| GCM_Time period | % Lost | % Gained | % Range Change |
| --- | --- | --- | --- |
| CanESM _30 | 0 | 80.13 | 403.32 |
| CanESM _50 | 0 | 85.45 | 587.39 |
| CanESM _70 | 0 | 89.37 | 840.48 |
| ACCESS_30 | 0 | 68.39 | 216.34 |
| ACCESS_50 | 0 | 81.25 | 433.38 |
| ACCESS_70 | 0 | 86.08 | 618.19 |
| GFDL_30 | 0 | 78.01 | 354.69 |
| GFDL_50 | 0 | 82.61 | 474.93 |
| GFDL_70 | 0 | 86.05 | 616.86 |
| MIROC_30 | 0 | 67.55 | 208.18 |
| MIROC_50 | 0 | 78.99 | 375.94 |
| MIROC_70 | 0 | 83.03 | 489.30 |
| HadGEM2_30 | 0 | 78.18 | 358.34 |
| HadGEM2_50 | 0 | 84.06 | 527.50 |
| HadGEM2_70 | 0 | 87.71 | 714.05 |
| NorEsm_30 | 0 | 53.92 | 117.01 |
| NorEsm_50 | 0 | 71.97 | 256.81 |
| NorEsm_70 | 0 | 79.62 | 390.06 |

(2) *Bactorcera bryoniae:*

| GCM Time period | % Lost | % Gained | % Range Changed |
| --- | --- | --- | --- |
| CanESM_30 | 12.95 | 14.19 | 01.43 |
| CanESM_50 | 18.76 | 15.16 | -04.23 |
| CanESM_70 | 26.79 | 29.71 | 04.15 |
| ACCESS_30 | 12.12 | 14.83 | 03.18 |
| ACCESS_50 | 10.13 | 19.41 | 11.51 |
| ACCESS_70 | 15.94 | 30.64 | 21.19 |
| GFDL_30 | 14.37 | 15.87 | 01.79 |
| GFDL_50 | 19.09 | 10.07 | -10.03 |
| GFDL_70 | 23.45 | 10.06 | -14.88 |
| MIROC_30 | 17.29 | 12.12 | -05.88 |
| MIROC_50 | 14.99 | 17.60 | 03.17 |
| MIROC_70 | 03.59 | 39.19 | 58.52 |
| HadGEM2_30 | 07.35 | 19.49 | 15.07 |
| HadGEM2_50 | 06.12 | 20.96 | 18.78 |
| HadGEM2_70 | 12.99 | 30.05 | 24.39 |
| NorEsm_30 | 01.55 | 23.09 | 28.00 |
| NorEsm_50 | 00.62 | 29.37 | 40.69 |
| NorEsm_70 | 00.84 | 45.36 | 81.49 |

(4) *Bactrocera frauenfeldi:*

| GCM Time period | % Lost | % Gained | % Range Changed |
| --- | --- | --- | --- |
| CanESM_30 | 21.92 | 10.81 | -12.46 |
| CanESM_50 | 30.60 | 18.78 | -14.56 |
| CanESM_70 | 24.29 | 36.68 | 19.55 |
| ACCESS_30 | 00.09 | 43.62 | 77.19 |
| ACCESS_50 | 02.94 | 29.48 | 37.64 |
| ACCESS_70 | 09.09 | 32.47 | 34.60 |
| GFDL_30 | 02.41 | 14.56 | 14.22 |
| GFDL_50 | 02.67 | 21.66 | 24.23 |
| GFDL_70 | 00.00 | 39.45 | 65.13 |
| MIROC_30 | 01.89 | 40.14 | 63.88 |
| MIROC_50 | 09.87 | 34.67 | 37.95 |
| MIROC_70 | 21.08 | 21.52 | 00.56 |
| HadGEM_30 | 13.81 | 20.99 | 09.09 |
| HadGEM_50 | 27.66 | 30.31 | 03.80 |
| HadGEM_70 | 16.27 | 30.25 | 20.04 |
| NorEsm_30 | 10.50 | 18.47 | 09.77 |
| NorEsm_50 | 01.36 | 33.74 | 48.87 |
| NorEsm_70 | 00.00 | 51.98 | 108.25 |

(5) *Bactrocera halfordiae:*

| GCM Time period | % Lost | % Gained | % Range Changed |
| --- | --- | --- | --- |
| CanESM_30 | 65.13 | 35.11 | -46.27 |
| CanESM_50 | 73.22 | 56.79 | -38.03 |
| CanESM_70 | 81.39 | 73.69 | -29.26 |
| ACCESS_30 | 70.09 | 45.93 | -44.69 |
| ACCESS_50 | 66.38 | 56.48 | -22.76 |
| ACCESS_70 | 52.41 | 58.67 | 15.14 |
| GFDL_30 | 86.21 | 35.98 | -78.47 |
| GFDL_50 | 89.27 | 44.40 | -80.69 |
| GFDL_70 | 89.66 | 51.29 | -78.76 |
| MIROC_30 | 79.19 | 07.92 | -77.41 |
| MIROC_50 | 53.56 | 38.73 | -24.20 |
| MIROC_70 | 15.09 | 56.39 | 94.68 |
| HadGEM2_30 | 51.42 | 28.35 | -32.19 |
| HadGEM2_50 | 48.04 | 51.83 | 07.85 |
| HadGEM2_70 | 47.19 | 65.21 | 51.75 |
| NorEsm_30 | 05.57 | 40.84 | 59.61 |
| NorEsm_50 | 39.38 | 43.86 | 07.99 |
| NorEsm_70 | 39.27 | 62.21 | 60.74 |

(6) *Bactrocera jarvisi:*

| GCM Time period | % Lost | % Gained | % Range Changed |
| --- | --- | --- | --- |
| CanESM_30 | 08.96 | 45.53 | 67.14 |
| CanESM_50 | 10.62 | 45.53 | 82.82 |
| CanESM_70 | 10.31 | 58.77 | 117.54 |
| ACCESS_30 | 11.35 | 35.79 | 38.08 |
| ACCESS_50 | 05.02 | 49.72 | 88.88 |
| ACCESS_70 | 07.19 | 62.46 | 147.19 |
| GFDL_30 | 11.09 | 44.18 | 59.26 |
| GFDL_50 | 12.64 | 49.32 | 72.39 |
| GFDL_70 | 10.72 | 55.06 | 98.68 |
| MIROC_30 | 13.79 | 41.39 | 47.08 |
| MIROC_50 | 09.70 | 54.00 | 96.32 |
| MIROC_70 | 00.55 | 66.26 | 194.74 |
| HadGEM_30 | 04.87 | 46.93 | 79.26 |
| HadGEM_50 | 02.40 | 55.42 | 118.91 |
| HadGEM_70 | 00.69 | 65.52 | 188.06 |
| NorEsm_30 | 00.12 | 31.01 | 44.78 |
| NorEsm_50 | 1.63E-05 | 44.55 | 80.34 |
| NorEsm_70 | 00.01 | 64.58 | 182.29 |

(7) *Bactrocera kraussi:*

| GCM Time period | % Lost | % Gained | % Range Changed |
| --- | --- | --- | --- |
| CanESM_30 | 14.86 | 01.48 | -13.58 |
| CanESM_50 | 13.59 | 02.95 | -10.96 |
| CanESM_70 | 09.37 | 16.42 | 08.43 |
| ACCESS_30 | 00.79 | 21.96 | 27.11 |
| ACCESS_50 | 01.17 | 14.20 | 15.18 |
| ACCESS_70 | 02.41 | 16.87 | 17.39 |
| GFDL_30 | 04.95 | 05.22 | 00.29 |
| GFDL_50 | 02.91 | 08.59 | 06.22 |
| GFDL_70 | 02.27 | 18.89 | 20.51 |
| MIROC_30 | 00.90 | 16.79 | 19.09 |
| MIROC_50 | 04.43 | 13.08 | 09.96 |
| MIROC_70 | 00.28 | 15.35 | 17.79 |
| HadGEM2_30 | 07.03 | 11.66 | 05.23 |
| HadGEM2_50 | 04.45 | 22.59 | 23.43 |
| HadGEM2_70 | 01.65 | 22.25 | 26.50 |
| NorEsm_30 | 06.52 | 07.89 | 01.49 |
| NorEsm_50 | 02.43 | 17.23 | 17.88 |
| NorEsm_70 | 01.41 | 29.61 | 40.06 |

(8) *Bactrocera musae:*

| GCM Time period | % Lost | % Gained | % Range Changed |
| --- | --- | --- | --- |
| CanESM_30 | 22.26 | 00.32 | -22.01 |
| CanESM_50 | 22.01 | 03.89 | -18.85 |
| CanESM_70 | 15.03 | 22.59 | 09.77 |
| ACCESS_30 | 01.83 | 32.98 | 46.49 |
| ACCESS_50 | 03.35 | 14.55 | 13.11 |
| ACCESS_70 | 07.00 | 16.46 | 11.32 |
| GFDL_30 | 11.73 | 07.68 | -04.38 |
| GFDL_50 | 03.41 | 11.99 | 09.75 |
| GFDL_70 | 01.46 | 29.76 | 40.30 |
| MIROC_30 | 01.37 | 31.25 | 43.47 |
| MIROC_50 | 05.11 | 21.77 | 21.29 |
| MIROC_70 | 04.39 | 15.73 | 13.45 |
| HadGEM_30 | 20.89 | 17.25 | -04.41 |
| HadGEM_50 | 11.81 | 24.58 | 16.93 |
| HadGEM_70 | 11.46 | 24.61 | 17.45 |
| NorEsm_30 | 13.95 | 13.75 | -00.23 |
| NorEsm_50 | 03.26 | 26.72 | 32.01 |
| NorEsm_70 | 00.16 | 43.55 | 76.87 |

(9) *Bactrocera neohumeralis:*

| GCM Time period | % Lost | % Gained | % Range Changed |
| --- | --- | --- | --- |
| CanESM_30 | 24.16 | 03.68 | -12.14 |
| CanESM_50 | 24.12 | 19.95 | -05.21 |
| CanESM_70 | 25.39 | 39.59 | 23.50 |
| ACCESS_30 | 25.19 | 31.29 | 08.88 |
| ACCESS_50 | 22.88 | 27.91 | 06.98 |
| ACCESS_70 | 22.27 | 41.41 | 32.66 |
| GFDL_30 | 32.49 | 08.34 | -26.34 |
| GFDL_50 | 27.87 | 21.08 | -08.60 |
| GFDL_70 | 26.68 | 34.80 | 12.45 |
| MIROC_30 | 23.26 | 32.47 | 13.64 |
| MIROC_50 | 16.50 | 37.67 | 33.95 |
| MIROC_70 | 02.51 | 63.15 | 164.59 |
| HadGEM_30 | 14.33 | 17.85 | 04.28 |
| HadGEM_50 | 06.11 | 42.29 | 62.69 |
| HadGEM_70 | 03.43 | 47.89 | 85.33 |
| NorEsm_30 | 04.71 | 36.27 | 49.52 |
| NorEsm_50 | 02.44 | 35.39 | 50.99 |
| NorEsm_70 | 02.84 | 55.22 | 116.97 |

(10) *Bactrocera tryoni:*

| GCM Time period | % Lost | % Gained | % Range Changed |
| --- | --- | --- | --- |
| CanESM_30 | 30.18 | 17.38 | -15.49 |
| CanESM_50 | 37.32 | 23.10 | -18.49 |
| CanESM_70 | 33.68 | 31.54 | -03.13 |
| ACCESS_30 | 26.09 | 11.10 | -16.87 |
| ACCESS_50 | 20.25 | 16.71 | -04.25 |
| ACCESS_70 | 17.18 | 26.14 | 12.13 |
| GFDL_30 | 47.89 | 15.71 | -38.18 |
| GFDL_50 | 52.52 | 20.29 | -40.43 |
| GFDL_70 | 51.22 | 24.18 | -35.67 |
| MIROC_30 | 36.04 | 16.22 | -23.66 |
| MIROC_50 | 27.59 | 19.56 | -09.98 |
| MIROC_70 | 09.15 | 36.86 | 43.89 |
| HadGEM_30 | 36.71 | 16.73 | -23.99 |
| HadGEM_50 | 29.87 | 21.65 | -10.50 |
| HadGEM_70 | 14.39 | 27.37 | 17.87 |
| NorEsm_30 | 16.01 | 16.44 | 00.51 |
| NorEsm_50 | 15.13 | 20.22 | 06.38 |
| NorEsm_70 | 06.13 | 40.12 | 56.76 |

(11) *Ceratitis capitata:*

| GCM Time period | % Lost | % Gained | % Range Changed |
| --- | --- | --- | --- |
| CanESM_30 | 16.51 | 15.76 | -00.89 |
| CanESM_50 | 17.52 | 23.16 | 07.34 |
| CanESM_70 | 17.58 | 40.29 | 38.02 |
| ACCESS_30 | 10.29 | 17.26 | 08.41 |
| ACCESS_50 | 15.95 | 35.04 | 29.39 |
| ACCESS_70 | 17.54 | 52.96 | 75.29 |
| GFDL_30 | 20.85 | 07.78 | -14.17 |
| GFDL_50 | 22.37 | 11.52 | -12.26 |
| GFDL_70 | 17.76 | 24.83 | 09.41 |
| MIROC_30 | 09.37 | 20.46 | 13.95 |
| MIROC_50 | 11.91 | 32.26 | 30.05 |
| MIROC_70 | 17.75 | 53.61 | 77.30 |
| HadGEM_30 | 19.64 | 09.69 | -11.02 |
| HadGEM_50 | 20.14 | 15.62 | -05.35 |
| HadGEM_70 | 18.41 | 43.79 | 45.18 |
| NorEsm_30 | 09.71 | 17.67 | 09.67 |
| NorEsm_50 | 10.73 | 25.38 | 19.64 |
| NorEsm_70 | 15.19 | 55.48 | 90.52 |

(3) *Zeugodacus cucumis:*

| GCM Time period | % Lost | % Gained | % Range Changed |
| --- | --- | --- | --- |
| CanESM_30 | 04.47 | 52.09 | 99.41 |
| CanESM_50 | 03.43 | 58.08 | 130.34 |
| CanESM_70 | 01.72 | 67.63 | 203.59 |
| ACCESS_30 | 00.00 | 43.33 | 76.45 |
| ACCESS_50 | 00.00 | 68.73 | 219.79 |
| ACCESS_70 | 00.06 | 68.22 | 214.43 |
| GFDL_30 | 08.26 | 44.66 | 65.77 |
| GFDL_50 | 09.75 | 52.92 | 91.71 |
| GFDL_70 | 10.32 | 57.65 | 111.77 |
| MIROC_30 | 04.20 | 51.81 | 98.80 |
| MIROC_50 | 00.02 | 57.86 | 137.23 |
| MIROC_70 | 00.01 | 78.26 | 359.92 |
| HadGEM2_30 | 05.42 | 51.22 | 93.88 |
| HadGEM2_50 | 01.67 | 58.42 | 136.46 |
| HadGEM2_70 | 00.00 | 76.09 | 318.29 |
| NorEsm_30 | 00.97 | 47.51 | 88.66 |
| NorEsm_50 | 00.60 | 62.77 | 166.97 |
| NorEsm_70 | 00.08 | 80.39 | 409.58 |

**S3 Table. Area (km²) and percentage of Australia projected to be suitable for 11 fruit flies under six future climate scenarios.**  In the column ‘Climate scenarios’, 0 refers to the area projected to be unsuitable across all six scenarios; 1 refers to the area projected to be suitable under any one of the six scenarios…6 refers to the area projected to be suitable under all six scenarios.

| Species | Climate scenarios | 2030  1000 km² | 2030 (%) | 2050  1000 km² | 2050 (%) | 2070  1000 km² | 2070 (%) |
| --- | --- | --- | --- | --- | --- | --- | --- |
| *Bactrocera aquilonis* | 0 | 6,027 | 78.5 | 5,433 | 70.8 | 4,606 | 60 |
|  | 1 | 106 | 1.4 | 189 | 2.5 | 419 | 5.5 |
|  | 2 | 109 | 1.4 | 178 | 2.3 | 281 | 3.7 |
|  | 3 | 350 | 4.6 | 142 | 1.9 | 58 | 0.8 |
|  | 4 | 129 | 1.7 | 179 | 2.3 | 387 | 5 |
|  | 5 | 248 | 3.2 | 393 | 5.1 | 331 | 4.3 |
|  | 6 | 704 | 9.2 | 1,159 | 15.1 | 1,592 | 20.7 |
| *Bactrocera bryoniae* | 0 | 6,635 | 86.5 | 6,596 | 86 | 6,093 | 79.4 |
|  | 1 | 220 | 2.9 | 205 | 2.7 | 523 | 6.8 |
|  | 2 | 83 | 1.1 | 104 | 1.4 | 180 | 2.4 |
|  | 3 | 58 | 0.8 | 75 | 1 | 156 | 2 |
|  | 4 | 50 | 0.7 | 77 | 1 | 101 | 1.3 |
|  | 5 | 82 | 1.1 | 87 | 1.1 | 120 | 1.6 |
|  | 6 | 546 | 7.1 | 529 | 6.9 | 498 | 6.5 |
| *Bactrocera frauenfeldi* | 0 | 7,423 | 96.7 | 7,434 | 96.9 | 7,366 | 96 |
|  | 1 | 43 | 0.6 | 41 | 0.5 | 80 | 1 |
|  | 2 | 31 | 0.4 | 27 | 0.3 | 37 | 0.5 |
|  | 3 | 32 | 0.4 | 26 | 0.3 | 42 | 0.5 |
|  | 4 | 16 | 0.2 | 26 | 0.3 | 23 | 0.3 |
|  | 5 | 46 | 0.6 | 32 | 0.4 | 24 | 0.3 |
|  | 6 | 83 | 1.1 | 87 | 1.1 | 102 | 1.3 |
| *Bactrocera halfordiae* | 0 | 7,146 | 93.1 | 7,191 | 93.7 | 6,917 | 90.1 |
|  | 1 | 277 | 3.6 | 165 | 2.2 | 213 | 2.8 |
|  | 2 | 92 | 1.2 | 89 | 1.2 | 147 | 1.9 |
|  | 3 | 53 | 0.7 | 68 | 0.9 | 163 | 2.1 |
|  | 4 | 45 | 0.6 | 60 | 0.8 | 101 | 1.3 |
|  | 5 | 34 | 0.4 | 53 | 0.7 | 80 | 1 |
|  | 6 | 26 | 0.3 | 46 | 0.6 | 52 | 0.7 |
| *Bactrocera jarvisi* | 0 | 5,688 | 74.1 | 5,374 | 70 | 4,274 | 55.7 |
|  | 1 | 323 | 4.2 | 270 | 3.5 | 677 | 8.8 |
|  | 2 | 172 | 2.2 | 196 | 2.6 | 314 | 4.1 |
|  | 3 | 171 | 2.2 | 170 | 2.2 | 288 | 3.7 |
|  | 4 | 129 | 1.7 | 186 | 2.4 | 280 | 3.7 |
|  | 5 | 242 | 3.1 | 308 | 4 | 292 | 3.8 |
|  | 6 | 948 | 12.4 | 1,169 | 15.2 | 1,549 | 20.2 |
| *Bactrocera kraussi* | 0 | 7,432 | 96.9 | 7,416 | 96.7 | 7,386 | 96.3 |
|  | 1 | 20 | 0.3 | 29 | 0.4 | 38 | 0.5 |
|  | 2 | 18 | 0.2 | 18 | 0.2 | 21 | 0.3 |
|  | 3 | 16 | 0.2 | 21 | 0.3 | 19 | 0.3 |
|  | 4 | 17 | 0.2 | 16 | 0.2 | 16 | 0.2 |
|  | 5 | 30 | 0.4 | 28 | 0.4 | 31 | 0.4 |
|  | 6 | 140 | 1.8 | 145 | 1.9 | 162 | 2.1 |
| *Bactrocera musae* | 0 | 7,406 | 96.5 | 7,418 | 96.7 | 7,347 | 95.8 |
|  | 1 | 46 | 0.6 | 33 | 0.4 | 74 | 1 |
|  | 2 | 36 | 0.5 | 27 | 0.4 | 36 | 0.5 |
|  | 3 | 22 | 0.3 | 23 | 0.3 | 36 | 0.5 |
|  | 4 | 22 | 0.3 | 25 | 0.3 | 25 | 0.3 |
|  | 5 | 45 | 0.6 | 39 | 0.5 | 25 | 0.3 |
|  | 6 | 97 | 1.3 | 109 | 1.4 | 130 | 1.7 |
| *Bactrocera neohumeralis* | 0 | 7,140 | 93 | 7,031 | 91.6 | 6,598 | 86 |
|  | 1 | 158 | 2.1 | 193 | 2.5 | 440 | 5.7 |
|  | 2 | 74 | 1 | 112 | 1.5 | 159 | 2.1 |
|  | 3 | 44 | 0.6 | 41 | 0.5 | 136 | 1.8 |
|  | 4 | 38 | 0.5 | 53 | 0.7 | 61 | 0.8 |
|  | 5 | 50 | 0.7 | 51 | 0.7 | 70 | 0.9 |
|  | 6 | 170 | 2.2 | 192 | 2.5 | 210 | 2.7 |
| *Bactrocera tryoni* | 0 | 5,673 | 73.9 | 5,567 | 72.5 | 4,638 | 60.4 |
|  | 1 | 471 | 6.1 | 399 | 5.2 | 772 | 10.1 |
|  | 2 | 175 | 2.3 | 189 | 2.5 | 291 | 3.8 |
|  | 3 | 144 | 1.9 | 207 | 2.7 | 292 | 3.8 |
|  | 4 | 174 | 2.3 | 245 | 3.2 | 350 | 4.6 |
|  | 5 | 305 | 4 | 256 | 3.3 | 336 | 4.4 |
|  | 6 | 731 | 9.5 | 810 | 10.6 | 994 | 13 |
| *Ceratitis capitata* | 0 | 5,501 | 71.7 | 5,104 | 66.5 | 4,056 | 52.9 |
|  | 1 | 336 | 4.4 | 475 | 6.2 | 554 | 7.2 |
|  | 2 | 180 | 2.3 | 321 | 4.2 | 406 | 5.3 |
|  | 3 | 159 | 2.1 | 194 | 2.5 | 402 | 5.2 |
|  | 4 | 206 | 2.7 | 205 | 2.7 | 474 | 6.2 |
|  | 5 | 220 | 2.9 | 250 | 3.3 | 447 | 5.8 |
|  | 6 | 1,070 | 13.9 | 1,123 | 14.6 | 1,334 | 17.4 |
| *Zeugodacus cucumis* | 0 | 6,536 | 85.2 | 6,117 | 79.7 | 4,958 | 64.6 |
|  | 1 | 218 | 2.8 | 249 | 3.3 | 779 | 10.2 |
|  | 2 | 123 | 1.6 | 276 | 3.6 | 424 | 5.5 |
|  | 3 | 109 | 1.4 | 157 | 2 | 268 | 3.5 |
|  | 4 | 76 | 1 | 99 | 1.3 | 252 | 3.3 |
|  | 5 | 107 | 1.4 | 172 | 2.2 | 202 | 2.6 |
|  | 6 | 504 | 6.6 | 602 | 7.9 | 790 | 10.3 |

**S4 Table. Major commercial fruits and vegetables host species to the Australian Horticulture Statistics Handbook (HSHB;** [**www.horticulture.com.au**](http://www.horticulture.com.au/)**).** Pest status is based on Hancock et al^3^, where “major” indicates that there have been many records of the fly infesting that host.

| **Fruit fly species** | **Scientific name** | **Common name** | **Key region** | **Latitude** | **Longitude** | **State** | **Reference** | **Pest status** |
| --- | --- | --- | --- | --- | --- | --- | --- | --- |
| *Bactrocera aquilonis* | *Capsicum annuum* | bell pepper | Bowen | -20.014 | 148.248 | Queensland | 1 | major |
| *Bactrocera aquilonis* | *Capsicum annuum* | bell pepper | Bundaberg | -24.866 | 152.348 | Queensland | 1 | major |
| *Bactrocera aquilonis* | *Capsicum annuum* | bell pepper | Carnarvon | -24.881 | 113.659 | Western Australia | 1 | major |
| *Bactrocera aquilonis* | *Solanum lycopersicum* | tomato | Bowen | -20.014 | 148.248 | Queensland | 1 | major |
| *Bactrocera aquilonis* | *Solanum lycopersicum* | tomato | Bundaberg | -24.866 | 152.348 | Queensland | 1 | major |
| *Bactrocera aquilonis* | *Solanum lycopersicum* | tomato | Lockyer Valley | -27.628 | 152.169 | Queensland | 1 | major |
| *Bactrocera aquilonis* | *Solanum lycopersicum* | tomato | Goulburn Valley | -37.034 | 145.125 | Victoria | 1 | major |
| *Bactrocera aquilonis* | *Citrus × limon* | lemon | Mareeba | -16.995 | 145.423 | Queensland | 1 | major |
| *Bactrocera aquilonis* | *Citrus × limon* | lemon | Burnett | -24.767 | 152.4 | Queensland | 1 | major |
| *Bactrocera aquilonis* | *Citrus × limon* | lemon | Bundaberg | -24.866 | 152.348 | Queensland | 1 | major |
| *Bactrocera aquilonis* | *Citrus × limon* | lemon | Lismore | -28.814 | 153.277 | New South Wales | 1 | major |
| *Bactrocera aquilonis* | *Citrus × limon* | lemon | Riverland | -34.25 | 140.467 | South Australia | 1 | major |
| *Bactrocera aquilonis* | *Citrus × limon* | lemon | Darwin | -12.463 | 130.842 | Northern Territory | 1 | major |
| *Bactrocera aquilonis* | *Citrus reticulata* | mandarin | Mareeba | -16.995 | 145.423 | Queensland | 1 | major |
| *Bactrocera aquilonis* | *Citrus reticulata* | mandarin | Emerald | -23.523 | 148.158 | Queensland | 1 | major |
| *Bactrocera aquilonis* | *Citrus reticulata* | mandarin | Mundubbera | -25.593 | 151.302 | Queensland | 1 | major |
| *Bactrocera aquilonis* | *Citrus reticulata* | mandarin | Murray Valley | -36.141 | 144.761 | Victoria | 1 | major |
| *Bactrocera aquilonis* | *Citrus reticulata* | mandarin | Riverland | -34.25 | 140.467 | South Australia | 1 | major |
| *Bactrocera aquilonis* | *Citrus × paradisi* | grapefruit | Murray Valley | -36.141 | 144.761 | Victoria | 1 | major |
| *Bactrocera aquilonis* | *Citrus × paradisi* | grapefruit | Riverina | -35 | 146 | New South Wales | 1 | major |
| *Bactrocera aquilonis* | *Citrus × paradisi* | grapefruit | Central Burnett | -24.767 | 152.4 | Queensland | 1 | major |
| *Bactrocera aquilonis* | *Citrus × paradisi* | grapefruit | Riverland region | -34.25 | 140.467 | South Australia | 1 | major |
| *Bactrocera aquilonis* | *Citrus × paradisi* | grapefruit | Perth region | -31.954 | 115.857 | Western Australia | 1 | major |
| *Bactrocera aquilonis* | *Malus domestica* | apple | Stanthorpe | -28.667 | 151.95 | Queensland | 1 | major |
| *Bactrocera aquilonis* | *Malus domestica* | apple | Batlow | -35.517 | 148.167 | New South Wales | 1 | major |
| *Bactrocera aquilonis* | *Malus domestica* | apple | Orange | -33.284 | 149.101 | New South Wales | 1 | major |
| *Bactrocera aquilonis* | *Malus domestica* | apple | Goulburn Valley | -37.034 | 145.125 | Victoria | 1 | major |
| *Bactrocera aquilonis* | *Malus domestica* | apple | Gippsland | -38.267 | 146.741 | Victoria | 1 | major |
| *Bactrocera aquilonis* | *Malus domestica* | apple | Yarra Valley | -37.657 | 145.447 | Victoria | 1 | major |
| *Bactrocera aquilonis* | *Malus domestica* | apple | Mornington Peninsula | -38.285 | 145.093 | Victoria | 1 | major |
| *Bactrocera aquilonis* | *Malus domestica* | apple | Huon Valley | -43.033 | 147.033 | Tasmania | 1 | major |
| *Bactrocera aquilonis* | *Malus domestica* | apple | Donnybrook | -33.577 | 115.821 | Western Australia | 1 | major |
| *Bactrocera aquilonis* | *Malus domestica* | apple | Manjimup | -34.241 | 116.146 | Western Australia | 1 | major |
| *Bactrocera aquilonis* | *Malus domestica* | apple | Adelaide Hills | -34.911 | 138.707 | South Australia | 1 | major |
| *Bactrocera aquilonis* | *Mangifera indica* | mango | Darwin | -12.463 | 130.842 | Northern Territory | 1 | major |
| *Bactrocera aquilonis* | *Mangifera indica* | mango | Katherine | -14.465 | 132.264 | Northern Territory | 1 | major |
| *Bactrocera aquilonis* | *Mangifera indica* | mango | Bowen | -20.014 | 148.248 | Queensland | 1 | major |
| *Bactrocera aquilonis* | *Mangifera indica* | mango | Bundaberg | -24.866 | 152.348 | Queensland | 1 | major |
| *Bactrocera aquilonis* | *Mangifera indica* | mango | Mareeba | -16.995 | 145.423 | Queensland | 1 | major |
| *Bactrocera aquilonis* | *Mangifera indica* | mango | Kununurra | -15.778 | 128.744 | Western Australia | 1 | major |
| *Bactrocera aquilonis* | *Prunus persica* | peach | Goulburn Valley | -37.034 | 145.125 | Victoria | 1 | major |
| *Bactrocera aquilonis* | *Prunus persica* | peach | Sunraysia | -34.204 | 142.135 | Victoria | 1 | major |
| *Bactrocera aquilonis* | *Prunus persica* | peach | Orange | -33.284 | 149.101 | New South Wales | 1 | major |
| *Bactrocera aquilonis* | *Prunus persica* | peach | Young | -34.314 | 148.298 | New South Wales | 1 | major |
| *Bactrocera bryoniae* | *Capsicum annuum* | chilli | Bowen | -20.014 | 148.248 | Queensland | 2 | major |
| *Bactrocera bryoniae* | *Capsicum annuum* | chilli | Bundaberg | -24.866 | 152.348 | Queensland | 2 | major |
| *Bactrocera bryoniae* | *Capsicum annuum* | chilli | Carnarvon | -24.881 | 113.659 | Western Australia | 2 | major |
| *Bactrocera bryoniae* | *Capsicum annuum* | chilli | Mildura | -34.207 | 142.137 | Victoria | 2 | major |
| *Bactrocera bryoniae* | *Solanum lycopersicum* | tomato | Bowen | -20.014 | 148.248 | Queensland | 2 | major |
| *Bactrocera bryoniae* | *Solanum lycopersicum* | tomato | Bundaberg | -24.866 | 152.348 | Queensland | 2 | major |
| *Bactrocera bryoniae* | *Solanum lycopersicum* | tomato | Lockyer Valley | -27.628 | 152.169 | Queensland | 2 | major |
| *Bactrocera bryoniae* | *Solanum lycopersicum* | tomato | Goulburn Valley | -37.034 | 145.125 | Victoria | 2 | major |
| *Bactrocera frauenfeldi* | *Mangifera indica* | mango | Darwin | -12.463 | 130.842 | Northern Territory | 3 | major |
| *Bactrocera frauenfeldi* | *Mangifera indica* | mango | Katherine | -14.465 | 132.264 | Northern Territory | 3 | major |
| *Bactrocera frauenfeldi* | *Mangifera indica* | mango | Bowen | -20.014 | 148.248 | Queensland | 3 | major |
| *Bactrocera frauenfeldi* | *Mangifera indica* | mango | Bundaberg | -24.866 | 152.348 | Queensland | 3 | major |
| *Bactrocera frauenfeldi* | *Mangifera indica* | mango | Mareeba | -16.995 | 145.423 | Queensland | 3 | major |
| *Bactrocera frauenfeldi* | *Mangifera indica* | mango | Kununurra | -15.778 | 128.744 | Western Australia | 3 | major |
| *Bactrocera frauenfeldi* | *Passiflora edulis* | passionfruit | Cooktown | -15.467 | 145.283 | Queensland | 3 | major |
| *Bactrocera frauenfeldi* | *Passiflora edulis* | passionfruit | Daintree | -16.25 | 145.317 | Queensland | 3 | major |
| *Bactrocera frauenfeldi* | *Passiflora edulis* | passionfruit | Mareeba | -16.995 | 145.423 | Queensland | 3 | major |
| *Bactrocera frauenfeldi* | *Passiflora edulis* | passionfruit | Sunshine Coast | -26.656 | 153.092 | Queensland | 3 | major |
| *Bactrocera frauenfeldi* | *Passiflora edulis* | passionfruit | Tweed Valley | -28.183 | 153.55 | New South Wales | 3 | major |
| *Bactrocera frauenfeldi* | *Musa × paradisiaca* | banana | Tully | -17.933 | 145.933 | Queensland | 3 | major |
| *Bactrocera frauenfeldi* | *Musa × paradisiaca* | banana | Innisfail | -17.522 | 146.031 | Queensland | 3 | major |
| *Bactrocera frauenfeldi* | *Musa × paradisiaca* | banana | Lakeland | -15.817 | 145 | Queensland | 3 | major |
| *Bactrocera frauenfeldi* | *Musa × paradisiaca* | banana | Bundaberg | -24.866 | 152.348 | Queensland | 3 | major |
| *Bactrocera frauenfeldi* | *Musa × paradisiaca* | banana | Darwin | -12.463 | 130.842 | Northern Territory | 3 | major |
| *Bactrocera frauenfeldi* | *Musa × paradisiaca* | banana | Coffs Harbour | -30.302 | 153.119 | New South Wales | 3 | major |
| *Bactrocera frauenfeldi* | *Musa × paradisiaca* | banana | Carnarvon region | -24.881 | 113.659 | Western Australia | 3 | major |
| *Bactrocera frauenfeldi* | *Citrus reticulata* | mandarin | Mareeba | -16.995 | 145.423 | Queensland | 3 | major |
| *Bactrocera frauenfeldi* | *Citrus reticulata* | mandarin | Emerald | -23.523 | 148.158 | Queensland | 3 | major |
| *Bactrocera frauenfeldi* | *Citrus reticulata* | mandarin | Mundubbera | -25.593 | 151.302 | Queensland | 3 | major |
| *Bactrocera frauenfeldi* | *Citrus reticulata* | mandarin | Murray Valley | -36.141 | 144.761 | Victoria | 3 | major |
| *Bactrocera frauenfeldi* | *Citrus sinensis* | orange | Riverina | -35 | 146 | New South Wales | 3 | major |
| *Bactrocera frauenfeldi* | *Citrus sinensis* | orange | Murray Valley | -36.141 | 144.761 | Victoria | 3 | major |
| *Bactrocera frauenfeldi* | *Citrus sinensis* | orange | Riverland | -34.25 | 140.467 | South Australia | 3 | major |
| *Bactrocera frauenfeldi* | *Capsicum annuum* | chilli | Bowen | -20.014 | 148.248 | Queensland | 3 | major |
| *Bactrocera frauenfeldi* | *Capsicum annuum* | chilli | Bundaberg | -24.866 | 152.348 | Queensland | 3 | major |
| *Bactrocera frauenfeldi* | *Capsicum annuum* | chilli | Carnarvon | -24.881 | 113.659 | Western Australia | 3 | major |
| *Bactrocera frauenfeldi* | *Capsicum annuum* | chilli | Mildura | -34.207 | 142.137 | Victoria | 3 | major |
| *Bactrocera halfordiae* | *Citrus × paradisi* | grapefruit | Murray Valley | -36.141 | 144.761 | Victoria | 2 | major |
| *Bactrocera halfordiae* | *Citrus × paradisi* | grapefruit | Riverina | -35 | 146 | New South Wales | 2 | major |
| *Bactrocera halfordiae* | *Citrus × paradisi* | grapefruit | Central Burnett | -24.767 | 152.4 | Queensland | 2 | major |
| *Bactrocera halfordiae* | *Citrus × paradisi* | grapefruit | Riverland region | -34.25 | 140.467 | South Australia | 2 | major |
| *Bactrocera halfordiae* | *Citrus × paradisi* | grapefruit | Perth region | -31.954 | 115.857 | Western Australia | 2 | major |
| *Bactrocera halfordiae* | *Citrus reticulata* | mandarin | Mareeba | -16.995 | 145.423 | Queensland | 2 | major |
| *Bactrocera halfordiae* | *Citrus reticulata* | mandarin | Emerald | -23.523 | 148.158 | Queensland | 2 | major |
| *Bactrocera halfordiae* | *Citrus reticulata* | mandarin | Mundubbera | -25.593 | 151.302 | Queensland | 2 | major |
| *Bactrocera halfordiae* | *Citrus reticulata* | mandarin | Murray Valley | -36.141 | 144.761 | Victoria | 2 | major |
| *Bactrocera halfordiae* | *Citrus reticulata* | mandarin | Riverland | -34.25 | 140.467 | South Australia | 2 | major |
| *Bactrocera halfordiae* | *Citrus reticulata* | mandarin | Murray Valley | -36.141 | 144.761 | Victoria | 2 | major |
| *Bactrocera halfordiae* | *Citrus sinensis* | orange | Riverina | -35 | 146 | New South Wales | 2 | major |
| *Bactrocera halfordiae* | *Citrus sinensis* | orange | Murray Valley | -36.141 | 144.761 | Victoria | 2 | major |
| *Bactrocera halfordiae* | *Citrus sinensis* | orange | Riverland | -34.25 | 140.467 | South Australia | 2 | major |
| *Bactrocera jarvisi* | *Mangifera indica* | mango | Darwin | -12.463 | 130.842 | Northern Territory | 2 | major |
| *Bactrocera jarvisi* | *Mangifera indica* | mango | Katherine | -14.465 | 132.264 | Northern Territory | 2 | major |
| *Bactrocera jarvisi* | *Mangifera indica* | mango | Bowen | -20.014 | 148.248 | Queensland | 2 | major |
| *Bactrocera jarvisi* | *Mangifera indica* | mango | Bundaberg | -24.866 | 152.348 | Queensland | 2 | major |
| *Bactrocera jarvisi* | *Mangifera indica* | mango | Mareeba | -16.995 | 145.423 | Queensland | 2 | major |
| *Bactrocera jarvisi* | *Mangifera indica* | mango | Kununurra | -15.778 | 128.744 | Western Australia | 2 | major |
| *Bactrocera jarvisi* | *Prunus persica* | peach | Goulburn Valley | -37.034 | 145.125 | Victoria | 2 | major |
| *Bactrocera jarvisi* | *Prunus persica* | peach | Sunraysia | -34.204 | 142.135 | Victoria | 2 | major |
| *Bactrocera jarvisi* | *Prunus persica* | peach | Orange | -33.284 | 149.101 | New South Wales | 2 | major |
| *Bactrocera jarvisi* | *Prunus persica* | peach | Young | -34.314 | 148.298 | New South Wales | 2 | major |
| *Bactrocera jarvisi* | *Musa × paradisiaca* | banana | Tully | -17.933 | 145.933 | Queensland | 2 | major |
| *Bactrocera jarvisi* | *Musa × paradisiaca* | banana | Innisfail | -17.522 | 146.031 | Queensland | 2 | major |
| *Bactrocera jarvisi* | *Musa × paradisiaca* | banana | Lakeland | -15.817 | 145 | Queensland | 2 | major |
| *Bactrocera jarvisi* | *Musa × paradisiaca* | banana | Bundaberg | -24.866 | 152.348 | Queensland | 2 | major |
| *Bactrocera jarvisi* | *Musa × paradisiaca* | banana | Darwin | -12.463 | 130.842 | Northern Territory | 2 | major |
| *Bactrocera jarvisi* | *Musa × paradisiaca* | banana | Coffs Harbour | -30.302 | 153.119 | New South Wales | 2 | major |
| *Bactrocera jarvisi* | *Musa × paradisiaca* | banana | Carnarvon region | -24.881 | 113.659 | Western Australia | 2 | major |
| *Bactrocera jarvisi* | *Malus domestica* | apple | Stanthorpe | -28.667 | 151.95 | Queensland | 2 | major |
| *Bactrocera jarvisi* | *Malus domestica* | apple | Batlow | -35.517 | 148.167 | New South Wales | 2 | major |
| *Bactrocera jarvisi* | *Malus domestica* | apple | Orange | -33.284 | 149.101 | New South Wales | 2 | major |
| *Bactrocera jarvisi* | *Malus domestica* | apple | Goulburn Valley | -37.034 | 145.125 | Victoria | 2 | major |
| *Bactrocera jarvisi* | *Malus domestica* | apple | Gippsland | -38.267 | 146.741 | Victoria | 2 | major |
| *Bactrocera jarvisi* | *Malus domestica* | apple | Yarra Valley | -37.657 | 145.447 | Victoria | 2 | major |
| *Bactrocera jarvisi* | *Malus domestica* | apple | Mornington Peninsula | -38.285 | 145.093 | Victoria | 2 | major |
| *Bactrocera jarvisi* | *Malus domestica* | apple | Huon Valley | -43.033 | 147.033 | Tasmania | 2 | major |
| *Bactrocera jarvisi* | *Malus domestica* | apple | Donnybrook | -33.577 | 115.821 | Western Australia | 2 | major |
| *Bactrocera jarvisi* | *Malus domestica* | apple | Manjimup | -34.241 | 116.146 | Western Australia | 2 | major |
| *Bactrocera jarvisi* | *Malus domestica* | apple | Adelaide Hills | -34.911 | 138.707 | South Australia | 2 | major |
| *Bactrocera jarvisi* | *Pyrus communis* | pear | Goulburn Valley | -37.034 | 145.125 | Victoria | 2 | major |
| *Bactrocera jarvisi* | *Pyrus communis* | pear | Yarra Valley | -37.733 | 145.683 | Victoria | 2 | major |
| *Bactrocera jarvisi* | *Pyrus communis* | pear | Gippsland | -37.584 | 147.767 | Victoria | 2 | major |
| *Bactrocera jarvisi* | *Pyrus communis* | pear | Stanthorpe | -28.667 | 151.95 | Queensland | 2 | major |
| *Bactrocera jarvisi* | *Pyrus communis* | pear | Batlow | -35.517 | 148.15 | New South Wales | 2 | major |
| *Bactrocera jarvisi* | *Pyrus communis* | pear | Huon Valley | -43.033 | 147.033 | Tasmania | 2 | major |
| *Bactrocera jarvisi* | *Pyrus communis* | pear | Adelaide Hills | -34.911 | 138.707 | South Australia | 2 | major |
| *Bactrocera jarvisi* | *Pyrus communis* | pear | Manijmup | -34.241 | 116.146 | Western Australia | 2 | major |
| *Bactrocera jarvisi* | *Carica papaya* | pawpaw | Mareeba | -16.995 | 145.423 | Queensland | 2 | major |
| *Bactrocera jarvisi* | *Carica papaya* | pawpaw | Tully | -17.933 | 145.933 | Queensland | 2 | major |
| *Bactrocera jarvisi* | *Diospyros kaki* | persimmon | Lockyer Valley | -27.628 | 152.169 | Queensland | 2 | major |
| *Bactrocera jarvisi* | *Diospyros kaki* | persimmon | Sydney Basin | -33.865 | 151.21 | New South Wales | 2 | major |
| *Bactrocera jarvisi* | *Diospyros kaki* | persimmon | Sunraysia | -34.204 | 142.135 | Victoria | 2 | major |
| *Bactrocera jarvisi* | *Diospyros kaki* | persimmon | Goulburn Valley | -37.034 | 145.125 | Victoria | 2 | major |
| *Bactrocera jarvisi* | *Diospyros kaki* | persimmon | Murray valley | -36.141 | 144.761 | Victoria | 2 | major |
| *Bactrocera jarvisi* | *Diospyros kaki* | persimmon | Riverland | -34.25 | 140.467 | South Australia | 2 | major |
| *Bactrocera kraussi* | *Citrus × paradisi* | grapefruit | Murray Valley | -36.141 | 144.761 | Victoria | 2 | major |
| *Bactrocera kraussi* | *Citrus × paradisi* | grapefruit | Riverina | -35 | 146 | New South Wales | 2,3 | major |
| *Bactrocera kraussi* | *Citrus × paradisi* | grapefruit | Central Burnett | -24.767 | 152.4 | Queensland | 2,3 | major |
| *Bactrocera kraussi* | *Citrus × paradisi* | grapefruit | Riverland region | -34.25 | 140.467 | South Australia | 2,3 | major |
| *Bactrocera kraussi* | *Citrus × paradisi* | grapefruit | Perth region | -31.954 | 115.857 | Western Australia | 2,3 | major |
| *Bactrocera kraussi* | *Citrus reticulata* | mandarin | Mareeba | -16.995 | 145.423 | Queensland | 2,3 | major |
| *Bactrocera kraussi* | *Citrus reticulata* | mandarin | Emerald | -23.523 | 148.158 | Queensland | 2,3 | major |
| *Bactrocera kraussi* | *Citrus reticulata* | mandarin | Mundubbera | -25.593 | 151.302 | Queensland | 2,3 | major |
| *Bactrocera kraussi* | *Citrus reticulata* | mandarin | Murray Valley | -36.141 | 144.761 | Victoria | 2,3 | major |
| *Bactrocera kraussi* | *Citrus reticulata* | mandarin | Riverland | -34.25 | 140.467 | SA | 2,3 | major |
| *Bactrocera kraussi* | *Citrus sinensis* | orange | Riverina | -35 | 146 | New South Wales | 2,3 | major |
| *Bactrocera kraussi* | *Citrus sinensis* | orange | Murray Valley | -36.141 | 144.761 | Victoria | 2,3 | major |
| *Bactrocera kraussi* | *Citrus sinensis* | orange | Riverland | -34.25 | 140.467 | South Australia | 2,3 | major |
| *Bactrocera kraussi* | *Mangifera indica* | mango | Darwin | -12.463 | 130.842 | Northern Territory | 2,3 | major |
| *Bactrocera kraussi* | *Mangifera indica* | mango | Katherine | -14.465 | 132.264 | Northern Territory | 2,3 | major |
| *Bactrocera kraussi* | *Mangifera indica* | mango | Bowen | -20.014 | 148.248 | Queensland | 2,3 | major |
| *Bactrocera kraussi* | *Mangifera indica* | mango | Bundaberg | -24.866 | 152.348 | Queensland | 2,3 | major |
| *Bactrocera kraussi* | *Mangifera indica* | mango | Mareeba | -16.995 | 145.423 | Queensland | 2,3 | major |
| *Bactrocera kraussi* | *Mangifera indica* | mango | Kununurra | -15.778 | 128.744 | Western Australia | 2,3 | major |
| *Bactrocera kraussi* | *Musa × paradisiaca* | banana | Tully | -17.933 | 145.933 | Queensland | 2,3 | major |
| *Bactrocera kraussi* | *Musa × paradisiaca* | banana | Innisfail | -17.522 | 146.031 | Queensland | 2,3 | major |
| *Bactrocera kraussi* | *Musa × paradisiaca* | banana | Lakeland | -15.817 | 145 | Queensland | 2,3 | major |
| *Bactrocera kraussi* | *Musa × paradisiaca* | banana | Bundaberg | -24.866 | 152.348 | Queensland | 2,3 | major |
| *Bactrocera kraussi* | *Musa × paradisiaca* | banana | Darwin | -12.463 | 130.842 | Northern Territory | 2,3 | major |
| *Bactrocera kraussi* | *Musa × paradisiaca* | banana | Coffs Harbour | -30.302 | 153.119 | New South Wales | 2,3 | major |
| *Bactrocera kraussi* | *Musa × paradisiaca* | banana | Carnarvon region | -24.881 | 113.659 | Western Australia | 2,3 | major |
| *Bactrocera kraussi* | *Prunus persica* | peach | Tweed Valley | -28.183 | 153.55 | New South Wales | 2,3 | major |
| *Bactrocera kraussi* | *Prunus persica* | peach | Sunraysia | -34.204 | 142.135 | Victoria | 2,3 | major |
| *Bactrocera kraussi* | *Prunus persica* | peach | Orange | -33.284 | 149.101 | New South Wales | 2,3 | major |
| *Bactrocera kraussi* | *Prunus persica* | peach | Young | -34.314 | 148.298 | New South Wales | 2,3 | major |
| *Bactrocera musae* | *Musa × paradisiaca* | banana | Tully | -17.933 | 145.933 | Queensland | 2,3 | major |
| *Bactrocera musae* | *Musa × paradisiaca* | banana | Innisfail | -17.522 | 146.031 | Queensland | 2,3 | major |
| *Bactrocera musae* | *Musa × paradisiaca* | banana | Lakeland | -15.817 | 145 | Queensland | 2,3 | major |
| *Bactrocera musae* | *Musa × paradisiaca* | banana | Bundaberg | -24.866 | 152.348 | Queensland | 2,3 | major |
| *Bactrocera musae* | *Musa × paradisiaca* | banana | Darwin | -12.463 | 130.842 | Northern Territory | 2,3 | major |
| *Bactrocera musae* | *Musa × paradisiaca* | banana | Coffs Harbour | -30.302 | 153.119 | New South Wales | 2,3 | major |
| *Bactrocera musae* | *Musa × paradisiaca* | banana | Carnarvon region | -24.881 | 113.659 | Western Australia | 2,3 | major |
| *Bactrocera neohumeralis* | *Mangifera indica* | mango | Darwin | -12.463 | 130.842 | Northern Territory | 2 | major |
| *Bactrocera neohumeralis* | *Mangifera indica* | mango | Katherine | -14.465 | 132.264 | Northern Territory | 2 | major |
| *Bactrocera neohumeralis* | *Mangifera indica* | mango | Bowen | -20.014 | 148.248 | Queensland | 2 | major |
| *Bactrocera neohumeralis* | *Mangifera indica* | mango | Bundaberg | -24.866 | 152.348 | Queensland | 2 | major |
| *Bactrocera neohumeralis* | *Mangifera indica* | mango | Mareeba | -16.995 | 145.423 | Queensland | 2 | major |
| *Bactrocera neohumeralis* | *Mangifera indica* | mango | Kununurra | -15.778 | 128.744 | Western Australia | 2 | major |
| *Bactrocera neohumeralis* | *Prunus persica* | peach | Tweed Valley | -28.183 | 153.55 | New South Wales | 2 | major |
| *Bactrocera neohumeralis* | *Prunus persica* | peach | Sunraysia | -34.204 | 142.135 | Victoria | 2 | major |
| *Bactrocera neohumeralis* | *Prunus persica* | peach | Orange | -33.284 | 149.101 | New South Wales | 2 | major |
| *Bactrocera neohumeralis* | *Prunus persica* | peach | Young | -34.314 | 148.298 | New South Wales | 2 | major |
| *Bactrocera neohumeralis* | *Musa × paradisiaca* | banana | Tully | -17.933 | 145.933 | Queensland | 2 | major |
| *Bactrocera neohumeralis* | *Musa × paradisiaca* | banana | Innisfail | -17.522 | 146.031 | Queensland | 2 | major |
| *Bactrocera neohumeralis* | *Musa × paradisiaca* | banana | Lakeland | -15.817 | 145 | Queensland | 2 | major |
| *Bactrocera neohumeralis* | *Musa × paradisiaca* | banana | Bundaberg | -24.866 | 152.348 | Queensland | 2 | major |
| *Bactrocera neohumeralis* | *Musa × paradisiaca* | banana | Darwin | -12.463 | 130.842 | Northern Territory | 2 | major |
| *Bactrocera neohumeralis* | *Musa × paradisiaca* | banana | Coffs Harbour | -30.302 | 153.119 | New South Wales | 2 | major |
| *Bactrocera neohumeralis* | *Musa × paradisiaca* | banana | Carnarvon region | -24.881 | 113.659 | Western Australia | 2 | major |
| *Bactrocera neohumeralis* | *Carica papaya* | pawpaw | Mareeba | -16.995 | 145.423 | Queensland | 2 | major |
| *Bactrocera neohumeralis* | *Carica papaya* | pawpaw | Tully | -17.933 | 145.933 | Queensland | 2 | major |
| *Bactrocera neohumeralis* | *Diospyros kaki* | persimmon | Lockyer Valley | -27.628 | 152.169 | Queensland | 2 | major |
| *Bactrocera neohumeralis* | *Diospyros kaki* | persimmon | Sydney Basin | -33.865 | 151.21 | New South Wales | 2 | major |
| *Bactrocera neohumeralis* | *Diospyros kaki* | persimmon | Sunraysia | -34.204 | 142.135 | Victoria | 2 | major |
| *Bactrocera neohumeralis* | *Diospyros kaki* | persimmon | Goulburn Valley | -37.034 | 145.125 | Victoria | 2 | major |
| *Bactrocera neohumeralis* | *Diospyros kaki* | persimmon | Murray valley | -36.141 | 144.761 | Victoria | 2 | major |
| *Bactrocera neohumeralis* | *Diospyros kaki* | persimmon | Riverland | -34.25 | 140.467 | South Australia | 2 | major |
| *Bactrocera neohumeralis* | *Solanum lycopersicum* | tomato | Bowen | -20.014 | 148.248 | Queensland | 2 | major |
| *Bactrocera neohumeralis* | *Solanum lycopersicum* | tomato | Bundaberg | -24.866 | 152.348 | Queensland | 2 | major |
| *Bactrocera neohumeralis* | *Solanum lycopersicum* | tomato | Lockyer Valley | -27.628 | 152.169 | Queensland | 2 | major |
| *Bactrocera neohumeralis* | *Solanum lycopersicum* | tomato | Goulburn Valley | -37.034 | 145.125 | Victoria | 2 | major |
| *Bactrocera neohumeralis* | *Malus domestica* | apple | Stanthorpe | -28.667 | 151.95 | Queensland | 2 | major |
| *Bactrocera neohumeralis* | *Malus domestica* | apple | Batlow | -35.517 | 148.167 | New South Wales | 2 | major |
| *Bactrocera neohumeralis* | *Malus domestica* | apple | Orange | -33.284 | 149.101 | New South Wales | 2 | major |
| *Bactrocera neohumeralis* | *Malus domestica* | apple | Goulburn Valley | -37.034 | 145.125 | Victoria | 2 | major |
| *Bactrocera neohumeralis* | *Malus domestica* | apple | Gippsland | -38.267 | 146.741 | Victoria | 2 | major |
| *Bactrocera neohumeralis* | *Malus domestica* | apple | Yarra Valley | -37.657 | 145.447 | Victoria | 2 | major |
| *Bactrocera neohumeralis* | *Malus domestica* | apple | Mornington Peninsula | -38.285 | 145.093 | Victoria | 2 | major |
| *Bactrocera neohumeralis* | *Malus domestica* | apple | Huon Valley | -43.033 | 147.033 | Tasmania | 2 | major |
| *Bactrocera neohumeralis* | *Malus domestica* | apple | Donnybrook | -33.577 | 115.821 | Western Australia | 2 | major |
| *Bactrocera neohumeralis* | *Malus domestica* | apple | Manjimup | -34.241 | 116.146 | Western Australia | 2 | major |
| *Bactrocera neohumeralis* | *Malus domestica* | apple | Adelaide Hills | -34.911 | 138.707 | South Australia | 2 | major |
| *Bactrocera neohumeralis* | *Passiflora edulis* | passionfruit | Tully | -17.933 | 145.933 | Queensland | 2 | major |
| *Bactrocera neohumeralis* | *Passiflora edulis* | passionfruit | Bundaberg | -24.866 | 152.348 | Queensland | 2 | major |
| *Bactrocera neohumeralis* | *Passiflora edulis* | passionfruit | Cooktown | -15.467 | 145.283 | Queensland | 2 | major |
| *Bactrocera neohumeralis* | *Passiflora edulis* | passionfruit | Daintree | -16.25 | 145.317 | Queensland | 2 | major |
| *Bactrocera neohumeralis* | *Passiflora edulis* | passionfruit | Mareeba | -16.995 | 145.423 | Queensland | 2 | major |
| *Bactrocera neohumeralis* | *Passiflora edulis* | passionfruit | Sunshine Coast | -26.656 | 153.092 | Queensland | 2 | major |
| *Bactrocera neohumeralis* | *Persea americana* | avocado | Atherton Tablelands | -17.371 | 145.403 | Queensland | 2 | major |
| *Bactrocera neohumeralis* | *Persea americana* | avocado | Bundaberg | -24.866 | 152.348 | Queensland | 2 | major |
| *Bactrocera neohumeralis* | *Persea americana* | avocado | Sunraysia | -34.204 | 142.135 | Victoria | 2 | major |
| *Bactrocera neohumeralis* | *Persea americana* | avocado | Manjimup | -34.241 | 116.146 | Western Australia | 2 | major |
| *Bactrocera neohumeralis* | *Prunus armeniaca* | apricot | Goulburn Valley | -37.034 | 145.125 | Victoria | 2 | major |
| *Bactrocera neohumeralis* | *Prunus armeniaca* | apricot | Swan Hill | -35.333 | 143.549 | South Australia | 2 | major |
| *Bactrocera neohumeralis* | *Prunus armeniaca* | apricot | Renmark | -34.17 | 140.75 | South Australia | 2 | major |
| *Bactrocera neohumeralis* | *Prunus armeniaca* | apricot | Perth | -31.954 | 115.857 | Western Australia | 2 | major |
| *Bactrocera neohumeralis* | *Prunus subg. Prunus* | plum | Goulburn Valley | -37.034 | 145.125 | Victoria | 2 | major |
| *Bactrocera neohumeralis* | *Prunus subg. Prunus* | plum | Young | -34.314 | 148.298 | New South Wales | 2 | major |
| *Bactrocera neohumeralis* | *Prunus subg. Prunus* | plum | Orange | -33.284 | 149.101 | New South Wales | 2 | major |
| *Bactrocera neohumeralis* | *Prunus subg. Prunus* | plum | Perth | -31.954 | 115.857 | Western Australia | 2 | major |
| *Bactrocera neohumeralis* | *Citrus × paradisi* | grapefruit | Murray Valley | -36.141 | 144.761 | Victoria | 2 | major |
| *Bactrocera neohumeralis* | *Citrus × paradisi* | grapefruit | Riverina | -35 | 146 | New South Wales | 2 | major |
| *Bactrocera neohumeralis* | *Citrus × paradisi* | grapefruit | Central Burnett | -24.767 | 152.4 | Queensland | 2 | major |
| *Bactrocera neohumeralis* | *Citrus × paradisi* | grapefruit | Riverland region | -34.25 | 140.467 | South Australia | 2 | major |
| *Bactrocera neohumeralis* | *Citrus × paradisi* | grapefruit | Perth region | -31.954 | 115.857 | Western Australia | 2 | major |
| *Bactrocera neohumeralis* | *Citrus reticulata* | mandarin | Mareeba | -16.995 | 145.423 | Queensland | 2 | major |
| *Bactrocera neohumeralis* | *Citrus reticulata* | mandarin | Emerald | -23.523 | 148.158 | Queensland | 2 | major |
| *Bactrocera neohumeralis* | *Citrus reticulata* | mandarin | Mundubbera | -25.593 | 151.302 | Queensland | 2 | major |
| *Bactrocera neohumeralis* | *Citrus reticulata* | mandarin | Murray Valley | -36.141 | 144.761 | Victoria | 2 | major |
| *Bactrocera neohumeralis* | *Citrus reticulata* | mandarin | Riverland | -34.25 | 140.467 | SA | 2 | major |
| *Bactrocera neohumeralis* | *Citrus sinensis* | orange | Riverina | -35 | 146 | New South Wales | 2 | major |
| *Bactrocera neohumeralis* | *Citrus sinensis* | orange | Murray Valley | -36.141 | 144.761 | Victoria | 2 | major |
| *Bactrocera neohumeralis* | *Citrus sinensis* | orange | Riverland | -34.25 | 140.467 | South Australia | 2 | major |
| *Bactrocera neohumeralis* | *Solanum lycopersicum* | tomato | Bowen | -20.014 | 148.248 | Queensland | 2 | major |
| *Bactrocera neohumeralis* | *Solanum lycopersicum* | tomato | Bundaberg | -24.866 | 152.348 | Queensland | 2 | major |
| *Bactrocera neohumeralis* | *Solanum lycopersicum* | tomato | Lockyer Valley | -27.628 | 152.169 | Queensland | 2 | major |
| *Bactrocera neohumeralis* | *Solanum lycopersicum* | tomato | Goulburn Valley | -37.034 | 145.125 | Victoria | 2 | major |
| *Bactrocera neohumeralis* | *Capsicum annuum* | capsicum | Bowen | -20.014 | 148.248 | Queensland | 2 | major |
| *Bactrocera neohumeralis* | *Capsicum annuum* | capsicum | Bundaberg | -24.866 | 152.348 | Queensland | 2 | major |
| *Bactrocera neohumeralis* | *Capsicum annuum* | capsicum | Carnarvon | -24.881 | 113.659 | Western Australia | 2 | major |
| *Bactrocera neohumeralis* | *Capsicum annuum* | chilli | Bowen | -20.014 | 148.248 | Queensland | 2 | major |
| *Bactrocera neohumeralis* | *Capsicum annuum* | chilli | Bundaberg | -24.866 | 152.348 | Queensland | 2 | major |
| *Bactrocera neohumeralis* | *Capsicum annuum* | chilli | Carnarvon | -24.881 | 113.659 | Western Australia | 2 | major |
| *Bactrocera neohumeralis* | *Capsicum annuum* | chilli | Mildura | -34.207 | 142.137 | Victoria | 2 | major |
| *Bactrocera tryoni* | *Carica papaya* | pawpaw | Mareeba | -16.995 | 145.423 | Queensland | 2,5 | major |
| *Bactrocera tryoni* | *Carica papaya* | pawpaw | Tully | -17.933 | 145.933 | Queensland | 2,5 | major |
| *Bactrocera tryoni* | *Persea americana* | avocado | Atherton Tablelands | -17.371 | 145.403 | Queensland | 2,5 | major |
| *Bactrocera tryoni* | *Persea americana* | avocado | Bundaberg | -24.866 | 152.348 | Queensland | 2,5 | major |
| *Bactrocera tryoni* | *Persea americana* | avocado | Sunraysia | -34.204 | 142.135 | Victoria | 2,5 | major |
| *Bactrocera tryoni* | *Persea americana* | avocado | Manjimup | -34.241 | 116.146 | Western Australia | 2,5 | major |
| *Bactrocera tryoni* | *Musa × paradisiaca* | banana | Tully | -17.933 | 145.933 | Queensland | 2,5 | major |
| *Bactrocera tryoni* | *Musa × paradisiaca* | banana | Innisfail | -17.522 | 146.031 | Queensland | 2,5 | major |
| *Bactrocera tryoni* | *Musa × paradisiaca* | banana | Lakeland | -15.817 | 145 | Queensland | 2,5 | major |
| *Bactrocera tryoni* | *Musa × paradisiaca* | banana | Bundaberg | -24.866 | 152.348 | Queensland | 2,5 | major |
| *Bactrocera tryoni* | *Musa × paradisiaca* | banana | Darwin | -12.463 | 130.842 | Northern Territory | 2,5 | major |
| *Bactrocera tryoni* | *Musa × paradisiaca* | banana | Coffs Harbour | -30.302 | 153.119 | New South Wales | 2,5 | major |
| *Bactrocera tryoni* | *Musa × paradisiaca* | banana | Carnarvon region | -24.881 | 113.659 | Western Australia | 2,5 | major |
| *Bactrocera tryoni* | *Fragaria × ananassa* | strawberry | Yarra Valley | -37.733 | 145.683 | Victoria | 2,5 | major |
| *Bactrocera tryoni* | *Fragaria × ananassa* | strawberry | Beerwah | -26.899 | 152.883 | Queensland | 2,5 | major |
| *Bactrocera tryoni* | *Fragaria × ananassa* | strawberry | Camden | -34.054 | 150.695 | New South Wales | 2,5 | major |
| *Bactrocera tryoni* | *Fragaria × ananassa* | strawberry | Adelaide Hills | -34.911 | 138.707 | South Australia | 2,5 | major |
| *Bactrocera tryoni* | *Fragaria × ananassa* | strawberry | Wanneroo | -31.746 | 115.823 | Western Australia | 2,5 | major |
| *Bactrocera tryoni* | *Fragaria × ananassa* | strawberry | Bullsbrook | -31.663 | 116.029 | Western Australia | 2,5 | major |
| *Bactrocera tryoni* | *Fragaria × ananassa* | strawberry | Albany | -35.027 | 117.884 | Western Australia | 2,5 | major |
| *Bactrocera tryoni* | *Pyrus communis* | pear | Goulburn Valley | -37.034 | 145.125 | Victoria | 2,5 | major |
| *Bactrocera tryoni* | *Pyrus communis* | pear | Yarra Valley | -37.733 | 145.683 | Victoria | 2,5 | major |
| *Bactrocera tryoni* | *Pyrus communis* | pear | Gippsland | -37.584 | 147.767 | Victoria | 2,5 | major |
| *Bactrocera tryoni* | *Pyrus communis* | pear | Stanthorpe | -28.667 | 151.95 | Queensland | 2,5 | major |
| *Bactrocera tryoni* | *Pyrus communis* | pear | Batlow | -35.517 | 148.15 | New South Wales | 2,5 | major |
| *Bactrocera tryoni* | *Pyrus communis* | pear | Huon Valley | -43.033 | 147.033 | Tasmania | 2,5 | major |
| *Bactrocera tryoni* | *Pyrus communis* | pear | Adelaide Hills | -34.911 | 138.707 | South Australia | 2,5 | major |
| *Bactrocera tryoni* | *Pyrus communis* | pear | Manijmup | -34.241 | 116.146 | Western Australia | 2,5 | major |
| *Bactrocera tryoni* | *Diospyros kaki* | persimmon | Lockyer Valley | -27.628 | 152.169 | Queensland | 2,5 | major |
| *Bactrocera tryoni* | *Diospyros kaki* | persimmon | Sydney Basin | -33.865 | 151.21 | New South Wales | 2,5 | major |
| *Bactrocera tryoni* | *Diospyros kaki* | persimmon | Sunraysia | -34.204 | 142.135 | Victoria | 2,5 | major |
| *Bactrocera tryoni* | *Diospyros kaki* | persimmon | Goulburn Valley | -37.034 | 145.125 | Victoria | 2,5 | major |
| *Bactrocera tryoni* | *Diospyros kaki* | persimmon | Murray valley | -36.141 | 144.761 | Victoria | 2,5 | major |
| *Bactrocera tryoni* | *Diospyros kaki* | persimmon | Riverland | -34.25 | 140.467 | South Australia | 2,5 | major |
| *Bactrocera tryoni* | *Solanum lycopersicum* | tomato | Bowen | -20.014 | 148.248 | Queensland | 2,5 | major |
| *Bactrocera tryoni* | *Solanum lycopersicum* | tomato | Bundaberg | -24.866 | 152.348 | Queensland | 2,5 | major |
| *Bactrocera tryoni* | *Solanum lycopersicum* | tomato | Lockyer Valley | -27.628 | 152.169 | Queensland | 2,5 | major |
| *Bactrocera tryoni* | *Solanum lycopersicum* | tomato | Goulburn Valley | -37.034 | 145.125 | Victoria | 2,5 | major |
| *Bactrocera tryoni* | *Capsicum annuum* | capsicum | Bowen | -20.014 | 148.248 | Queensland | 2,5 | major |
| *Bactrocera tryoni* | *Capsicum annuum* | capsicum | Bundaberg | -24.866 | 152.348 | Queensland | 2,5 | major |
| *Bactrocera tryoni* | *Capsicum annuum* | capsicum | Carnarvon | -24.881 | 113.659 | Western Australia | 2,5 | major |
| *Bactrocera tryoni* | *Capsicum annuum* | chilli | Bowen | -20.014 | 148.248 | Queensland | 2,5 | major |
| *Bactrocera tryoni* | *Capsicum annuum* | chilli | Bundaberg | -24.866 | 152.348 | Queensland | 2,5 | major |
| *Bactrocera tryoni* | *Capsicum annuum* | chilli | Carnarvon | -24.881 | 113.659 | Western Australia | 2,5 | major |
| *Bactrocera tryoni* | *Capsicum annuum* | chilli | Mildura | -34.207 | 142.137 | Victoria | 2,5 | major |
| *Bactrocera tryoni* | *Malus domestica* | apple | Stanthorpe | -28.667 | 151.95 | Queensland | 2,5 | major |
| *Bactrocera tryoni* | *Malus domestica* | apple | Batlow | -35.517 | 148.167 | New South Wales | 2,5 | major |
| *Bactrocera tryoni* | *Malus domestica* | apple | Orange | -33.284 | 149.101 | New South Wales | 2,5 | major |
| *Bactrocera tryoni* | *Malus domestica* | apple | Goulburn Valley | -37.034 | 145.125 | Victoria | 2,5 | major |
| *Bactrocera tryoni* | *Malus domestica* | apple | Gippsland | -38.267 | 146.741 | Victoria | 2,5 | major |
| *Bactrocera tryoni* | *Malus domestica* | apple | Yarra Valley | -37.657 | 145.447 | Victoria | 2,5 | major |
| *Bactrocera tryoni* | *Malus domestica* | apple | Mornington Peninsula | -38.285 | 145.093 | Victoria | 2,5 | major |
| *Bactrocera tryoni* | *Malus domestica* | apple | Huon Valley | -43.033 | 147.033 | Tasmania | 2,5 | major |
| *Bactrocera tryoni* | *Malus domestica* | apple | Donnybrook | -33.577 | 115.821 | Western Australia | 2,5 | major |
| *Bactrocera tryoni* | *Malus domestica* | apple | Manjimup | -34.241 | 116.146 | Western Australia | 2,5 | major |
| *Bactrocera tryoni* | *Malus domestica* | apple | Adelaide Hills | -34.911 | 138.707 | South Australia | 2,5 | major |
| *Bactrocera tryoni* | *Mangifera indica* | mango | Darwin | -12.463 | 130.842 | Northern Territory | 2,5 | major |
| *Bactrocera tryoni* | *Mangifera indica* | mango | Katherine | -14.465 | 132.264 | Northern Territory | 2,5 | major |
| *Bactrocera tryoni* | *Mangifera indica* | mango | Bowen | -20.014 | 148.248 | Queensland | 2,5 | major |
| *Bactrocera tryoni* | *Mangifera indica* | mango | Bundaberg | -24.866 | 152.348 | Queensland | 2,5 | major |
| *Bactrocera tryoni* | *Mangifera indica* | mango | Mareeba | -16.995 | 145.423 | Queensland | 2,5 | major |
| *Bactrocera tryoni* | *Mangifera indica* | mango | Kununurra | -15.778 | 128.744 | Western Australia | 2,5 | major |
| *Bactrocera tryoni* | *Passiflora edulis* | passionfruit | Tully | -17.933 | 145.933 | Queensland | 2,5 | major |
| *Bactrocera tryoni* | *Passiflora edulis* | passionfruit | Bundaberg | -24.866 | 152.348 | Queensland | 2,5 | major |
| *Bactrocera tryoni* | *Passiflora edulis* | passionfruit | Cooktown | -15.467 | 145.283 | Queensland | 2,5 | major |
| *Bactrocera tryoni* | *Passiflora edulis* | passionfruit | Daintree | -16.25 | 145.317 | Queensland | 2,5 | major |
| *Bactrocera tryoni* | *Passiflora edulis* | passionfruit | Mareeba | -16.995 | 145.423 | Queensland | 2,5 | major |
| *Bactrocera tryoni* | *Passiflora edulis* | passionfruit | Sunshine Coast | -26.656 | 153.092 | Queensland | 2,5 | major |
| *Bactrocera tryoni* | *Prunus persica* | peach | Tweed Valley | -28.183 | 153.55 | New South Wales | 2,5 | major |
| *Bactrocera tryoni* | *Prunus persica* | peach | Sunraysia | -34.204 | 142.135 | Victoria | 2,5 | major |
| *Bactrocera tryoni* | *Prunus persica* | peach | Orange | -33.284 | 149.101 | New South Wales | 2,5 | major |
| *Bactrocera tryoni* | *Prunus persica* | peach | Young | -34.314 | 148.298 | New South Wales | 2,5 | major |
| *Bactrocera tryoni* | *Citrus × paradisi* | grapefruit | Murray Valley | -36.141 | 144.761 | Victoria | 2,5 | major |
| *Bactrocera tryoni* | *Citrus × paradisi* | grapefruit | Riverina | -35 | 146 | New South Wales | 2,5 | major |
| *Bactrocera tryoni* | *Citrus × paradisi* | grapefruit | Central Burnett | -24.767 | 152.4 | Queensland | 2,5 | major |
| *Bactrocera tryoni* | *Citrus × paradisi* | grapefruit | Riverland region | -34.25 | 140.467 | South Australia | 2,5 | major |
| *Bactrocera tryoni* | *Citrus × paradisi* | grapefruit | Perth region | -31.954 | 115.857 | Western Australia | 2,5 | major |
| *Bactrocera tryoni* | *Solanum melongena* | eggplant | Bowen | -20.014 | 148.248 | Queensland | 2,5 | major |
| *Bactrocera tryoni* | *Solanum melongena* | eggplant | Bundaberg | -24.866 | 152.348 | Queensland | 2,5 | major |
| *Bactrocera tryoni* | *Solanum melongena* | eggplant | Sydney region | -33.865 | 151.21 | New South Wales | 2,5 | major |
| *Bactrocera tryoni* | *Solanum melongena* | eggplant | Goulburn Valley | -37.034 | 145.125 | Victoria | 2,5 | major |
| *Bactrocera tryoni* | *Prunus persica var. nucipersica* | nectarine | Stanthorpe | -28.667 | 151.95 | Queensland | 2,5 | major |
| *Bactrocera tryoni* | *Prunus persica var. nucipersica* | nectarine | Sunraysia | -34.204 | 142.135 | Victoria | 2,5 | major |
| *Bactrocera tryoni* | *Prunus persica var. nucipersica* | nectarine | Orange | -33.284 | 149.101 | New South Wales | 2,5 | major |
| *Bactrocera tryoni* | *Prunus persica var. nucipersica* | nectarine | Young | -34.314 | 148.298 | New South Wales | 2,5 | major |
| *Ceratitis capitata* | *Mangifera indica* | mango | Darwin | -12.463 | 130.842 | Northern Territory | 2,4,5 | major |
| *Ceratitis capitata* | *Mangifera indica* | mango | Katherine | -14.465 | 132.264 | Northern Territory | 2,4,5 | major |
| *Ceratitis capitata* | *Mangifera indica* | mango | Bowen | -20.014 | 148.248 | Queensland | 2,4,5 | major |
| *Ceratitis capitata* | *Mangifera indica* | mango | Bundaberg | -24.866 | 152.348 | Queensland | 2,4,5 | major |
| *Ceratitis capitata* | *Mangifera indica* | mango | Mareeba | -16.995 | 145.423 | Queensland | 2,4,5 | major |
| *Ceratitis capitata* | *Mangifera indica* | mango | Kununurra | -15.778 | 128.744 | Western Australia | 2,4,5 | major |
| *Ceratitis capitata* | *Malus domestica* | apple | Stanthorpe | -28.667 | 151.95 | Queensland | 2,4,5 | major |
| *Ceratitis capitata* | *Malus domestica* | apple | Batlow | -35.517 | 148.167 | New South Wales | 2,4,5 | major |
| *Ceratitis capitata* | *Malus domestica* | apple | Orange | -33.284 | 149.101 | New South Wales | 2,4,5 | major |
| *Ceratitis capitata* | *Malus domestica* | apple | Goulburn Valley | -37.034 | 145.125 | Victoria | 2,4,5 | major |
| *Ceratitis capitata* | *Malus domestica* | apple | Gippsland | -38.267 | 146.741 | Victoria | 2,4,5 | major |
| *Ceratitis capitata* | *Malus domestica* | apple | Yarra Valley | -37.657 | 145.447 | Victoria | 2,4,5 | major |
| *Ceratitis capitata* | *Malus domestica* | apple | Mornington Peninsula | -38.285 | 145.093 | Victoria | 2,4,5 | major |
| *Ceratitis capitata* | *Malus domestica* | apple | Huon Valley | -43.033 | 147.033 | Tasmania | 2,4,5 | major |
| *Ceratitis capitata* | *Malus domestica* | apple | Donnybrook | -33.577 | 115.821 | Western Australia | 2,4,5 | major |
| *Ceratitis capitata* | *Malus domestica* | apple | Manjimup | -34.241 | 116.146 | Western Australia | 2,4,5 | major |
| *Ceratitis capitata* | *Malus domestica* | apple | Adelaide Hills | -34.911 | 138.707 | South Australia | 2,4,5 | major |
| *Ceratitis capitata* | *Carica papaya* | pawpaw | Mareeba | -16.995 | 145.423 | Queensland | 2,4,5 | major |
| *Ceratitis capitata* | *Carica papaya* | pawpaw | Tully | -17.933 | 145.933 | Queensland | 2,4,5 | major |
| *Ceratitis capitata* | *Prunus persica* | peach | Tweed Valley | -28.183 | 153.55 | New South Wales | 2,4,5 | major |
| *Ceratitis capitata* | *Prunus persica* | peach | Sunraysia | -34.204 | 142.135 | Victoria | 2,4,5 | major |
| *Ceratitis capitata* | *Prunus persica* | peach | Orange | -33.284 | 149.101 | New South Wales | 2,4,5 | major |
| *Ceratitis capitata* | *Prunus persica* | peach | Young | -34.314 | 148.298 | New South Wales | 2,4,5 | major |
| *Ceratitis capitata* | *Pyrus communis* | pear | Goulburn Valley | -37.034 | 145.125 | Victoria | 2,4,5 | major |
| *Ceratitis capitata* | *Pyrus communis* | pear | Yarra Valley | -37.733 | 145.683 | Victoria | 2,4,5 | major |
| *Ceratitis capitata* | *Pyrus communis* | pear | Gippsland | -37.584 | 147.767 | Victoria | 2,4,5 | major |
| *Ceratitis capitata* | *Pyrus communis* | pear | Stanthorpe | -28.667 | 151.95 | Queensland | 2,4,5 | major |
| *Ceratitis capitata* | *Pyrus communis* | pear | Batlow | -35.517 | 148.15 | New South Wales | 2,4,5 | major |
| *Ceratitis capitata* | *Pyrus communis* | pear | Huon Valley | -43.033 | 147.033 | Tasmania | 2,4,5 | major |
| *Ceratitis capitata* | *Pyrus communis* | pear | Adelaide Hills | -34.911 | 138.707 | South Australia | 2,4,5 | major |
| *Ceratitis capitata* | *Pyrus communis* | pear | Manijmup | -34.241 | 116.146 | Western Australia | 2,4,5 | major |
| *Ceratitis capitata* | *Citrus × paradisi* | grapefruit | Murray Valley | -36.141 | 144.761 | Victoria | 2,4,5 | major |
| *Ceratitis capitata* | *Citrus × paradisi* | grapefruit | Riverina | -35 | 146 | New South Wales | 2,4,5 | major |
| *Ceratitis capitata* | *Citrus × paradisi* | grapefruit | Central Burnett | -24.767 | 152.4 | Queensland | 2,4,5 | major |
| *Ceratitis capitata* | *Citrus × paradisi* | grapefruit | Riverland region | -34.25 | 140.467 | South Australia | 2,4,5 | major |
| *Ceratitis capitata* | *Citrus × paradisi* | grapefruit | Perth region | -31.954 | 115.857 | Western Australia | 2,4,5 | major |
| *Ceratitis capitata* | *Citrus reticulata* | mandarin | Mareeba | -16.995 | 145.423 | Queensland | 2,4,5 | major |
| *Ceratitis capitata* | *Citrus reticulata* | mandarin | Emerald | -23.523 | 148.158 | Queensland | 2,4,5 | major |
| *Ceratitis capitata* | *Citrus reticulata* | mandarin | Mundubbera | -25.593 | 151.302 | Queensland | 2,4,5 | major |
| *Ceratitis capitata* | *Citrus reticulata* | mandarin | Murray Valley | -36.141 | 144.761 | Victoria | 2,4,5 | major |
| *Ceratitis capitata* | *Citrus reticulata* | mandarin | Riverland | -34.25 | 140.467 | SA | 2,4,5 | major |
| *Ceratitis capitata* | *Citrus sinensis* | orange | Riverina | -35 | 146 | New South Wales | 2,4,5 | major |
| *Ceratitis capitata* | *Citrus sinensis* | orange | Murray Valley | -36.141 | 144.761 | Victoria | 2,4,5 | major |
| *Ceratitis capitata* | *Citrus sinensis* | orange | Riverland | -34.25 | 140.467 | South Australia | 2,4,5 | major |
| *Zeugodacus cucumis* | *Carica papaya* | pawpaw | Mareeba | -16.995 | 145.423 | Queensland | 2 | major |
| *Zeugodacus cucumis* | *Carica papaya* | pawpaw | Tully | -17.933 | 145.933 | Queensland | 2 | major |
| *Zeugodacus cucumis* | *Cucumis sativus* | cucumber | Bowen | -20.014 | 148.248 | Queensland | 2 | major |
| *Zeugodacus cucumis* | *Cucumis sativus* | cucumber | Bundaberg | -24.866 | 152.348 | Queensland | 2 | major |
| *Zeugodacus cucumis* | *Cucumis sativus* | cucumber | Riverland region | -34.25 | 140.467 | South Australia | 2 | major |
| *Zeugodacus cucumis* | *Cucurbita moschata* | pumpkin | Murrumbidgee region | -34.8 | 145.883 | New South Wales | 2 | major |
| *Zeugodacus cucumis* | *Cucurbita moschata* | pumpkin | Bundaberg | -24.866 | 152.348 | Queensland | 2 | major |
| *Zeugodacus cucumis* | *Cucurbita moschata* | pumpkin | Darling Downs region | -27.5 | 151.265 | Queensland | 2 | major |
| *Zeugodacus cucumis* | *Cucurbita pepo* | zucchini | Atherton Tablelands | -17.371 | 145.403 | Queensland | 2 | major |
| *Zeugodacus cucumis* | *Cucurbita pepo* | zucchini | Bowen | -20.014 | 148.248 | Queensland | 2 | major |
| *Zeugodacus cucumis* | *Cucurbita pepo* | zucchini | Bundaberg | -24.866 | 152.348 | Queensland | 2 | major |
| *Zeugodacus cucumis* | *Cucurbita pepo* | zucchini | Bathurst | -33.417 | 149.581 | New South Wales | 2 | major |
| *Zeugodacus cucumis* | *Cucurbita pepo* | zucchini | Sunraysia region | -34.204 | 142.135 | Victoria | 2 | major |
| *Zeugodacus cucumis* | *Cucurbita pepo* | zucchini | Perth | -31.954 | 115.857 | Western Australia | 2 | major |
| *Zeugodacus cucumis* | *Passiflora edulis* | passionfruit | Cooktown | -15.467 | 145.283 | Queensland | 2 | major |
| *Zeugodacus cucumis* | *Passiflora edulis* | passionfruit | Daintree | -16.25 | 145.317 | Queensland | 2 | major |
| *Zeugodacus cucumis* | *Passiflora edulis* | passionfruit | Mareeba | -16.995 | 145.423 | Queensland | 2 | major |
| *Zeugodacus cucumis* | *Passiflora edulis* | passionfruit | Sunshine Coast | -26.656 | 153.092 | Queensland | 2 | major |
| *Zeugodacus cucumis* | *Passiflora edulis* | passionfruit | Tweed Valley | -28.183 | 153.55 | New South Wales | 2 | major |
| *Zeugodacus cucumis* | *Solanum lycopersicum* | tomato | Bowen | -20.014 | 148.248 | Queensland | 2 | major |
| *Zeugodacus cucumis* | *Solanum lycopersicum* | tomato | Bundaberg | -24.866 | 152.348 | Queensland | 2 | major |
| *Zeugodacus cucumis* | *Solanum lycopersicum* | tomato | Lockyer Valley | -27.628 | 152.169 | Queensland | 2 | major |
| *Zeugodacus cucumis* | *Solanum lycopersicum* | tomato | Goulburn Valley | -37.034 | 145.125 | Victoria | 2 | major |
| *Zeugodacus cucumis* | *Cucurbita pepo* | squash/zucchini | Atherton Tablelands | -17.371 | 145.403 | Queensland | 2 | major |
| *Zeugodacus cucumis* | *Cucurbita pepo* | squash/zucchini | Bowen | -20.014 | 148.248 | Queensland | 2 | major |
| *Zeugodacus cucumis* | *Cucurbita pepo* | squash/zucchini | Bundaberg | -24.866 | 152.348 | Queensland | 2 | major |
| *Zeugodacus cucumis* | *Cucurbita pepo* | squash/zucchini | Bathurst | -33.417 | 149.581 | New South Wales | 2 | major |
| *Zeugodacus cucumis* | *Cucurbita pepo* | squash/zucchini | Sunraysia region | -34.204 | 142.135 | Victoria | 2 | major |
|  |  |  |  |  |  |  |  |  |

**References:**

1. Plant Health Australia. *The Australian Handbook for the Identification of Fruit Flies. Version 3.1.*, (Plant Health Australia. Canberra, ACT., 2018).
2. Hancock, D. L., Hamacek, E. L., Lloyd, A. C. & Elson-Harris, M. M. *The distribution and host plants of fruit flies (Diptera: Tephritidae) in Australia*. (Department of Primary Industries, Queensland, 2000).
3. Royer, J. E. & Hancock, D. L. New distribution and lure records of Dacinae (Diptera: Tephritidae) from Queensland, Australia, and description of a new species of *Dacus* Fabricius. *Australian Journal of Entomology* **51**, 239-247 (2012).
4. Dominiak, B. C. & Daniels, D. Review of the past and present distribution of Mediterranean fruit fly (*Ceratitis capitata* Wiedemann) and Queensland fruit fly (*Bactrocera tryoni* Froggatt) in Australia. *Australian Journal of Entomology* **51**, 104-115 (2012).
5. May, A. W. S. An investigation of fruit flies (Trypertidae: Diptera) in Queensland 1. Introduction, species, pest status and distribution. *Queensland Journal of Agricultural Science* **20**, 1-82 (1963).

**S5 Table. DOIs of the occurrence data for 11 tephritid fruit fly species downloaded from the GBIF database**

| 1.    DOI: <http://doi.org/10.15468/dl.kvaecc> |
| --- |
| Creation Date: Thursday, June 29, 2017 4:59:20 AM CEST        Filter used: TaxonKey: *Bactrocera bryoniae* (Tryon, 1927) |
|  |
| 2.    DOI: <http://doi.org/10.15468/dl.7ll9me>         Creation Date: Monday, July 3, 2017 12:22:56 PM CEST        Filter used: TaxonKey: *Bactrocera kraussi* (Hardy, 1951) |
|  |
| 3.    DOI: <http://doi.org/10.15468/dl.tyaphx> |
| Creation Date: Wednesday, June 28, 2017 4:55:59 PM CEST        Filter used: TaxonKey: *Bactrocera aquilonis* (May, 1965) |
|  |
| 4.    DOI: <http://doi.org/10.15468/dl.q4mv6v>         Creation Date: Wednesday, June 28, 2017 5:59:09 PM CEST        Filter used: TaxonKey: *Bactrocera cucumis* (French, 1907) |
|  |
| 5.    DOI: <http://doi.org/10.15468/dl.re8do1> |
| Creation Date: Thursday, June 29, 2017 4:31:49 AM CEST        Filter used: TaxonKey: *Bactrocera frauenfeldi* (Schiner, 1868) or *Bactrocera* |
| *cucumis* (French, 1907) or *Bactrocera halfordiae* (Tryon, 1927) or *Bactrocera* |
| *jarvisi* (Tryon, 1927) |
|  |
| 6.     DOI: <http://doi.org/10.15468/dl.tyll13>          Creation Date: Thursday, June 29, 2017 4:43:53 AM CEST         Filter used: TaxonKey: *Bactrocera jarvisi* (Tryon, 1927) |
|  |
| 7.     DOI: <http://doi.org/10.15468/dl.vvaofg> |
| Creation Date: Tuesday, July 4, 2017 3:07:55 AM CEST         Filter used: TaxonKey: *Bactrocera halfordiae* (Tryon, 1927) |
|  |
| 8.     DOI: <http://doi.org/10.15468/dl.iqifxg>          Creation Date: Tuesday, July 4, 2017 3:23:43 AM CEST         Filter used: TaxonKey: *Bactrocera musae* (Tryon, 1927) |
|  |
| 9.     DOI: <http://doi.org/10.15468/dl.drca1h>          Creation Date: Tuesday, July 4, 2017 3:33:43 AM CEST         Filter used: TaxonKey: *Bactrocera neohumeralis* (Hardy, 1951) |
|  |
| 10.   DOI: <http://doi.org/10.15468/dl.cuhilk>         Creation Date: Friday, July 14, 2017 6:45:42 AM CEST         Filter used: TaxonKey: *Ceratitis capitata* (Wiedemann, 1824) |
|  |
